# Implementation of an Ellipsoidal-Cavity Field Correction for Computed Molecular Oscillator Strengths in Solution: A(nother) Benchmark Study

**DOI:** 10.1021/acs.jctc.5c00070

**Published:** 2025-03-17

**Authors:** Jorge C. Garcia-Alvarez, Samer Gozem

**Affiliations:** Department of Chemistry, Georgia State University, Atlanta, Georgia 30302, United States

## Abstract

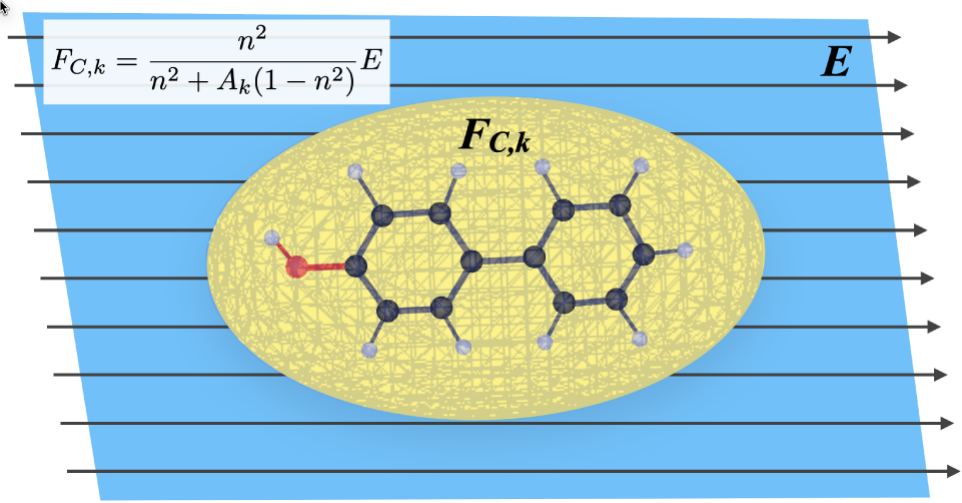

We recently compared oscillator strengths (OS) obtained from electronic structure calculations (*f*_comp_) to OSs derived from experimental spectra (*f*_exp_) multiplied by the refractive index (*n*) of the solution in which the spectra were measured. The choice of *nf*_exp_ instead of *f*_exp_ as a reference accounts for the macroscopic flux of energy in a dielectric (the experimental solvent). Here, we apply an approximate correction to *f*_comp_ values that accounts for the local electromagnetic field driving the absorption transition (which is generally different from the macroscopic field). We refer to these modified OSs as *f*_comp_^S^. The correction is obtained by assuming that each molecule occupies an ellipsoidal cavity, fitted to its van der Waals surface, surrounded by a continuum dielectric model representing the solvent. Sets ranging from 33 to 85 experimental transitions are used for the benchmark. For LR-CCSD and EOM-CCSD, we find that *f*_comp_^S^ generally gives a better agreement with experimental strengths than *f*_comp_. For LR-CCSD in the length gauge, for instance, there is a 1 to 1 scaling of the (*nf*_exp_, *f*_comp_^S^) pairs. Instead, the results for TD-DFT depend on the amount of HF exchange used in the functional: pure functionals typically also have a 1 to 1 scaling of the (*nf*_exp_, *f*_comp_^S^) pairs, while for hybrid functionals *f*_comp_^S^ overestimates *nf*_exp_ to a degree that appears proportional to the amount of HF exchange present in the functional.

## Introduction

Recently, we attempted to compare UV–vis absorption intensities computed with electronic structure methods to those derived from the experimental spectra of small organic molecules in solution.^1,2^ Such a comparison must account for the description of electromagnetic radiation in a dielectric medium. It is convenient to think about the experimental (apparent) absorption intensity in terms of the absorption cross-section—defined as *energy absorbed per unit time, per molecule*/*energy flux of the radiation field*. In a dielectric medium, the energy flux of a monochromatic wave is proportional to the square of the macroscopic electric field multiplied by the refractive index of the medium (*n* |**E**(ω)|^2^). On the other hand, the probability of absorption is proportional to the square of the field’s intensity along the direction of the transition dipole moment (TDM). While *in vacuo* these contributions of |**E**(ω)|^2^ cancel out, in the condensed phase the field driving the transition is not equal to the macroscopic field. This is discussed in more detail, for example, in ref (3).

In ref (2) we found optimal scaling factors *C* to minimize the difference between the pairs (*f*_comp_, *C*·*f*_exp_), where, for a given transition, *f*_comp_ is an oscillator strength (OS) computed with an electronic structure method while *f*_exp_ is the OS derived in ref (1) from experimental spectra. The *C* values were in general greater than 1 so that comparing *f*_comp_ to *nf*_exp_ resulted in smaller errors than comparing to *f*_exp_ or IUPAC’s recommended^4^*f*_exp_/*n*. The same observation has been made in a more recent study employing a different set of experimental data.^5^ Comparing *f*_comp_ to *nf*_exp_ is consistent with the factor *n* appearing in the energy flux of electromagnetic radiation, but assumes that the field driving the transition **F** is equal to the macroscopic field **E**.

In ref (2), we also tested several corrections found in the literature^6,7^ that, in addition to accounting for the energy flux, described the field acting on the absorbing solute molecule. The corrections employed either Lorentz’s^8^ expression for the local field  or Onsager’s^9^ expression for the cavity field . These local/cavity field expressions are obtained considering a spherical cavity around the solute molecule, outside which the solvent is described as a continuum dielectric. None of the tested expressions significantly improved the agreement between the computed and experimental OSs beyond the simple *f*_comp_ to *n f*_exp_ comparison.

Our benchmark set largely consists of π → π * transitions with TDMs along the longer dimension of conjugated systems. For different orientations of the TDM relative to the molecular dimensions, the same *f*_comp_ to *n f*_exp_ agreement should not be expected (see for example ref (10)).

One relatively simple improvement to the description of the field driving the transition is to consider an ellipsoidal instead of a spherical cavity. An analytical expression for the cavity field inside an ellipsoidal cavity in a continuum dielectric is given by^11,12^
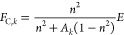
1where *F*_C,*k*_ is the cavity field along semiaxis *k* of the ellipsoid, *n* is the refractive index of the dielectric medium, *A*_*k*_ is a parameter depending on the relative length of axis *k* compared to the other two axes of the ellipsoid, and *E* is the macroscopic field in the dielectric. This expression is discussed in more detail in the *Methods* (*Interpolated ellipsoid* section). For the local (cavity + reaction) field along semiaxis *k*, the expression:
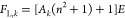
2was derived by Shibuya,^10^ who used it to explain the opposite changes in the apparent OSs of the π → π* transition in β-carotene, which decreases when absorption experiments are carried out in a solvent of higher refractive index, and the *n* → π* transition in pyrazine, that increases with higher *n* solvents.

Expression 2 for the local field accounts for both the cavity and reaction fields. It would be suitable to consider it for computations not accounting for the reaction field, but it is not appropriate to apply it to calculations in which the reaction field was already accounted for, such as the ones we carried out in ref (2) using PCM—that would result in a double-counting of the reaction field. In the present manuscript, we employ expression 1 to obtain a correction to the computed OSs and compare them to our experimental reference (*nf*_exp_). *A*_*k*_ values are obtained geometrically, by interpolating an ellipsoid from the PCM surface points of each molecular calculation. We recognize that the application of this continuous model at the scale of molecular van der Waals surfaces is a rough approximation. We also refer the readers to refs (1) and (2) for discussions on experimental and computational sources of error associated with oscillator strengths. Here, our goal is to better understand the sources of systematic errors in computed oscillator strengths after the application of an ellipsoidal cavity field model, which is an improvement on the field corrections derived from spherical cavities that we tested previously. At larger scales, the ellipsoidal cavity field model is expected to be more valid. Recently, it has been successfully applied to describe the nonlinear optical properties of cellulose nanocrystals.^13^

The theoretical background of our comparison as well as a summary of oscillator strength benchmark studies in the literature are presented in ref (2) and in the references therein, so we avoid repeating these topics here. A nonexhaustive list of benchmarks on oscillator strengths by others include refs (14−21).

Following this introduction, the Methods section is divided into three subsections that discuss i) computing OSs from electronic structure calculations, ii) fitting an ellipsoid to the surface of each molecule and correcting the computed OSs, and iii) the statistics employed for the comparison. This is followed by Results and Conclusions sections.

## Methods

### Computed Absorption Transitions

In this work, we apply the ellipsoidal cavity field corrections to some of our previous OS computations carried out in ref (2). There we started from geometries optimized with B3LYP/6-311++G**^22−24^ followed by single-point calculations with different wave function and time-dependent density functional theory (TD-DFT) methods. The wave function methods included configuration interaction singles (CIS),^25^ time-dependent Hartree–Fock (TD-HF),^26^ and equation of motion coupled cluster with singles and doubles (EOM-CCSD)^27,28^ as well as OSs from linear response transition densities (LR-CCSD).^29−31^ The nine functionals were SVWN,^32,33^ the hybrids B3P86,^22,34^ O3LYP,^23,35^ mPW1PW91,^36^ M05,^37^ and B3LYP^22,23^ and the long-range-corrected hybrids CAM-B3LYP,^38^ LC-ωHPBE,^39,40^ and ωB97X-D.^41^ The calculations with B3LYP under the Tamm-Dancoff approximation (TDA)^42^ are also revisited.

Here, we also expanded the benchmark to include the additional functionals: SVWN5,^32,33^ OLYP,^23,43^ BLYP,^23,44^ PBE,^45^ SOGGA11,^46^ N12,^47^ TPSS,^48^ PBE0,^49^ SOGGA11-X,^50^ and BHandHLYP.^51^ Two factors guided the selection of the new functionals: i) their performance predicting molecular dipole moments and polarizabilities^52^ and ii) the fact that among the original functionals, the pure SVWN and the hybrid O3LYP that has a small HF exchange yielded results differing from the other hybrid functionals.

The 6-311++G** basis set was used for all calculations, except for EOM and LR-CCSD for which aug-cc-pVDZ^53,54^ was used. Additional calculations with aug-cc-pVTZ and aug-pcseg-2^55^ were carried out with some functionals. These two basis sets have been found to perform significantly better than 6-311++G** for dipoles and polarizabilities.^56^ The aug-pcseg-2 basis set was downloaded from the Basis Set Exchange Web site.^57−59^ In a few of the larger molecules, calculations with aug-pcseg-2 failed to converge and so those molecules are left out of the corresponding analysis.

The effect of solvent on wave functions and energies was described through a polarizable continuum model (PCM) using the integral equation formalism (IEFPCM).^60^ More specifically, we used the nonequilibrium linear response (LR) solvation for TD-DFT calculations,^61,62^ while for EOM-CCSD and LR-CCSD calculations, we employed an approximation that uses solvent-polarized molecular orbitals but does not include the EOM-CCSD electronic response of the solvent.^63^ Other solvation models are available but have not been tested here.^64−69^ OSs are obtained from TDMs computed with the length and velocity operators as well as a mixture of these two that cancels the OSs’ explicit dependence on energy. The computed OSs are labeled length, momentum, or mixed gauge accordingly.

All calculations were performed with Gaussian 16 version C.01.^70^

### Interpolated Ellipsoid

To correct the computed OSs, we assume a simple model consisting of a continuum dielectric medium that represents the solvent and is characterized by the dielectric constant ϵ_1_. Inside it, an ellipsoidal cavity characterized by the dielectric constant ϵ_2_ represents the space occupied by the light-absorbing molecule. To geometrically describe such a cavity, we fit an ellipsoid to van der Waals surface points obtained from single-point PCM calculations. Wolfram Mathematica 14.0^71^ is used for the interpolation. The interpolated ellipsoid is given by the general quadric surface equation in Cartesian coordinates:
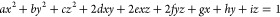
3where *x*, *y*, and *z* are the coordinates measured from a system of reference **O** that matches the one used by the electronic structure software. We assessed the quality of the fit resulting from the coefficients *a*, *b*, *c*, ..., *g*, *h*, *i* by looking at the residuals (see Supporting Information for more details). The interpolated ellipsoid can also be expressed using the canonical equation:
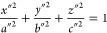
4where *x*″, *y*″, and *z*″ are now the coordinates measured from a system of reference **O″** concentric with the ellipsoid and with axes aligned with the ellipsoid major axes. *a*″, *b*″, and *c*″ are the lengths of the ellipsoid semiaxes, along the directions , , and , respectively. The system of reference **O″** can be obtained from **O** by a rotation (resulting in a system **O’**) followed by a translation. These operations, as well as the values of *a*″, *b*″, and *c*″, are determined by the matrix formed by the coefficients of eq 3.^72^

In the presence of a homogeneous macroscopic field **E** in medium 1, aligned with axis *k* of the ellipsoidal cavity (medium 2), the cavity field inside the ellipsoid is parallel to the external field **E**, and its magnitude is given by^11,12^
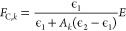
5with *k* = 1, 2, or 3 and
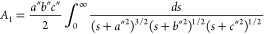
6corresponding to a field **E** polarized along the  direction,
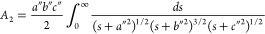
7for a field in the  direction, and
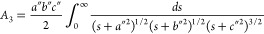
8for a field in the  direction.

The parameter *A*_1_ is always between 0 and 1. It is closer to 0 when *a*″ ≫ (*b*″ + *c*″), while it is closer to 1 when *a*″ ≪ (*b*″ + *c*″). Also, *A*_1_ + *A*_2_ + *A*_3_ = 1. Correspondingly, whenever ϵ_1_ > ϵ_2_ we have that *F*_C,1_/*E* ≥ 1 with the equality holding when *A*_1_ = 0. The same is true for *F*_C,2_ and *F*_C,3_.

For time-varying fields in typical solvents, it is justified to use^73^ ϵ(ω) = *n*(ω)^2^ where *n* is the refractive index of the medium. It is also justified to take the value of *n* at the sodium D line *n*_D_ as an approximation of the slowly varying, off-resonance component of *n*.^74,75^ To evaluate (5) we use ϵ_2_ = 1 since the absorbing molecule is treated explicitly in the electronic structure calculation and ϵ_1_ =  = *n*^2^ for the solvent. The use of ϵ_1_ = *n*^2^ for the cavity field expressions is consistent with nonequilibrium solvation models.

Since the probability of absorption is proportional to the field along the TDM direction, to correct the computed OS we need the cavity field along the direction of the TDM. In the Gaussian^70^ outputs considered in this manuscript, the TDMs are expressed in terms of their  components (system of reference **O**), and therefore we obtain the corrections along those directions (Corr_*x*_, Corr_*y*_, Corr_*z*_) as discussed below.

An arbitrarily oriented field **E** can be expressed in the **O″** system of reference as
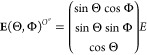
9where Θ is the polar angle relative to axis , Φ is the azimuthal angle in the plane defined by the  and  axes of the **O″** system of reference, and
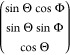
10is a unitary vector with components sin Θ cos Φ along the  axis, sin Θ sin Φ along the  axis, and cos Θ along the  axis so that its orientation is uniquely defined by Θ and Φ in the range (0 ≤ Θ ≤ π, 0 ≤ Φ < 2π).

Inside of the ellipsoid, the cavity field resulting from an external **E**(Θ,Φ) is given by
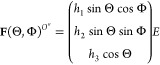
11where , *k* = 1, 2, 3.

By denoting  the vector  expressed in the  basis associated with the **O″** system of reference, we can obtain the correction factor along the  direction as an average of all possible orientations of **E** relative to the cavity:
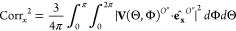
12where . The factor of 3 in eq 12 comes from the fact that computed OSs already contain a factor of 3 in the denominator to account for all possible orientations of **E**.^76^ The corrections along  and , Corr_*y*_ and Corr_*z*_, are equivalently obtained from (12) substituting  by  or  respectively.

We applied these corrections to the components of the TDM, which are reported along the , , and  directions in the electronic structure outputs. From the corrected components of the TDM, we recomputed the OS values for each transition. In this way, from each of the computed oscillator strengths *f*_*i*-0_ corresponding to the 0 → *i* transitions, the corrected values  are obtained. The superscript S stands for Scholte, the author of ref (12) from where we obtained expression (5).

**Figure 1:**
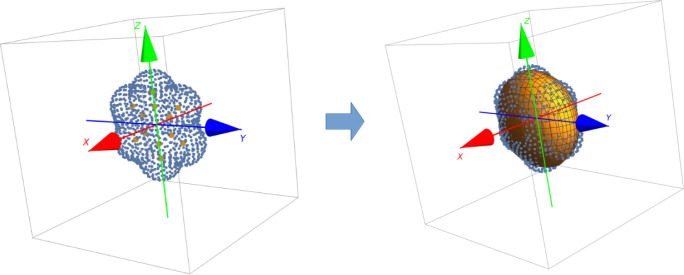
Diagram representing the interpolated ellipsoid for molecule 088 (Pyridinium) from the PCM surface points (blue dots) of the electronic structure calculations. Orange points (left figure) represent the coordinates of the molecule’s nuclei.

Table 1 reports the *A*_*k*_, *h*_*k*_, and Corr values for molecule 088, which is shown in Figure 1. The high symmetry of molecule 088 further simplifies the example since it is possible to choose axes  colinear with ,  colinear with , and  colinear with  (with the unprimed axes shown in Figure 1). Such colinearity also simplifies the values of Corr_*x*_^2^ to *h*_1_^2^, Corr_*y*_^2^ = *h*_2_^2^, and Corr_*z*_^2^ = *h*_3_^2^.

**Table 1:** Parameters Obtained from the Ellipsoid Interpolated for Molecule 088

Direction	k	Semiaxis length [Å]	*A*_*k*_	*h*_*k*_	Corr^2^
	1	2.169	0.485	1.269	1.610
	2	3.773	0.254	1.125	1.266
	3	3.694	0.261	1.129	1.274

Notice how a longer semiaxis (along ) is associated with a smaller *A*_*k*_ value and a correction Corr closer to 1 while the opposite happens for the shorter semiaxis. For the special case of a sphere then *A*_*k*_ = 1/3, and , matching Onsager’s expression for the cavity field.

The example above explains why we observed a reasonable agreement between *f*_comp_ and *nf*_exp_ in previous work^1,2^ even when no cavity field correction was employed, since most transitions involved π → π* character having TDMs along the long axis of the molecule. It also explains why applying a cavity field correction derived from a spherical cavity usually worsened the agreement with the experiments.^2^

### Statistical Analysis

The statistical analysis in this work follows the same approach employed in our earlier work (ref (2)) to quantify the agreement between computed and experimental OSs. A summary is provided below. A more detailed discussion can be found in ref (2).

For a given molecule, experimental OSs, *f*_exp, *k*_, were obtained in ref (1) using expression:^4,73^
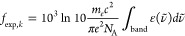
13where ε(ν̃) is the decadic molar extinction coefficient (also called absorption or attenuation coefficient) expressed in M^–1^ cm^–1^ and ν̃ is the wavenumber in cm^–1^. In other words, *f*_exp, *k*_ is obtained by fitting and integrating absorption bands over the range of wavenumbers spanning the band. The experimental spectra originally were obtained from ref (77).

Equation 13 is suitable for gas-phase experiments. Since the experimental data is obtained in solution, we multiply *f*_exp,*k*_ by *n* to account for the flux of energy of the macroscopic field in the dielectric (solvent),^2^ while the cavity field driving the transition is described via the corrections introduced above on *f*_comp,*k*_.

**Figure 2:**
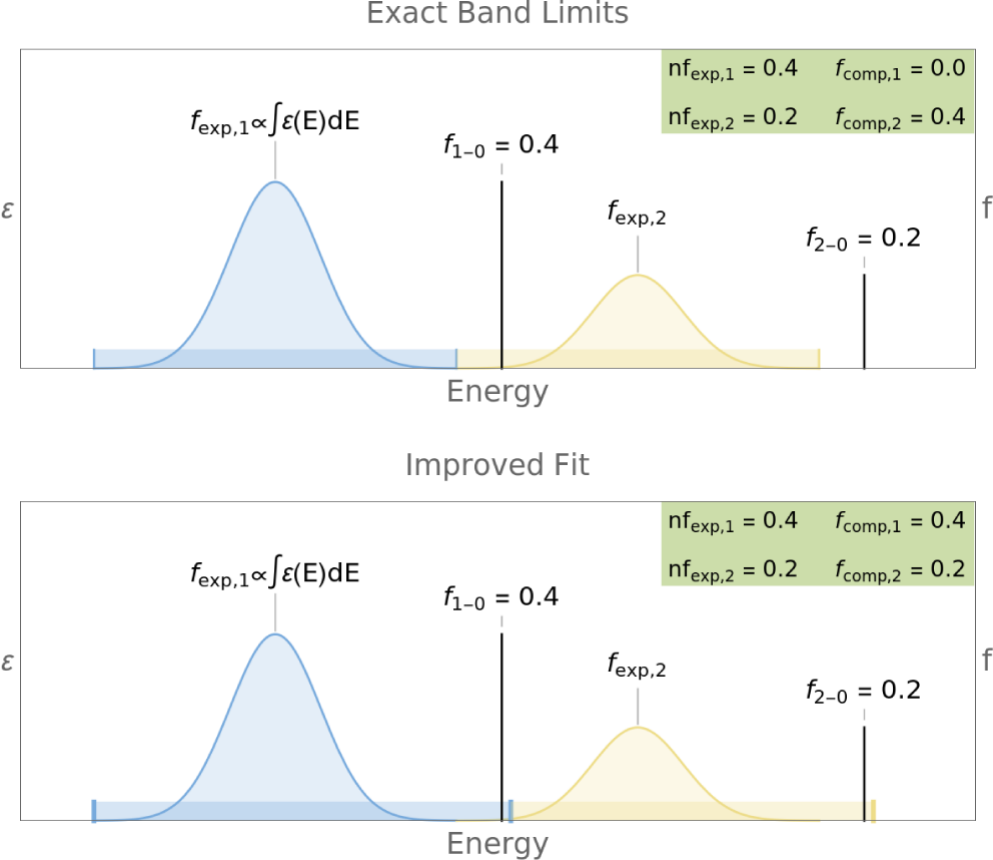
Illustration of the “Exact Band Limits” and “Improved Fit” modes of comparison. A simple and favorable scenario is presented.

The corresponding computed OS values are assigned to an experimental band based on the computed energy relative to the band. Initially, a computed *f*_*i*-0_ value is assigned to band *k* if the energy of the 0 → *i* transition lies within the limits of band *k*. Such *f*_*i*-0_ contributes additively to a *f*_comp,*k*_ value that is compared to *n f*_exp,*k*_. We label this comparison mode *Exact Band Limits* (EBL). The EBL mode is a true reflection of a method’s ability to reproduce both the absorption energy and strength of a band but often leads to misassignment of bands when energies are not accurate. Therefore, in a second instance, band *k*’s energy limits are shifted in such a way that the set of *f*_*i*-0_ contributing to *f*_comp, *k*_ results in the smallest difference between *f*_comp, *k*_ and *n f*_exp, *k*_. We label this comparison mode *Improved Fit* (IF). This mode is biased toward a favorable agreement, but is a useful indicator of an upper limit to the accuracy of a method’s computed OSs. Figure 2 illustrates both modes.

To quantify the agreement between *f*_comp, *k*_ and *n f*_exp, *k*_ the mean absolute error, MAE, is calculated as
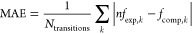
14where *N*_transitions_ is the total number of transitions. Linear regression analysis of the (*nf*_exp_, *f*_comp_) pairs is conducted and the resulting slope, intercept, and *R*^2^ are reported.

The energetic agreement between computed and experimental transitions is also monitored. An average experimental transition energy for a band *k* is obtained as
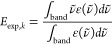
15using the ε(ν̃) data from ref (77) and digitized in ref (1). A reference computed energy for band *k* is obtained as the OS-weighted average:
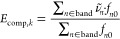
16

From (15) and (16) three metrics are reported to assess the energetic performance of a given method: (i) the mean absolute energy error:
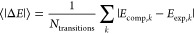
17ii) the mean energy error:
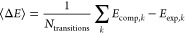
18and (iii) the mean ratio of computed to experimental energies:
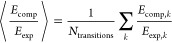
19

Monitoring the energies predicted by a given method is relevant to our OS comparison since individual *f*_*i*-0_ explicitly dependent on energy in the position and momentum gauges, but the quantities obtained from (17–19), also help understand the different transition assignments in the EBL and IF modes.

Finally, we summarize the agreement between computed and experimental OS by computing a scaling factor *C* that minimizes the MAE between the pairs (*Cnf*_exp,*k*_, *f*_comp,*k*_). This factor may be thought of as the slope of a linear regression through the origin in a scatter plot of the pairs (*nf*_exp,*k*_, *f*_comp,*k*_). However, obtaining *C* involves iterative calls to the Improved Fit Algorithm. Consider the algorithm below:
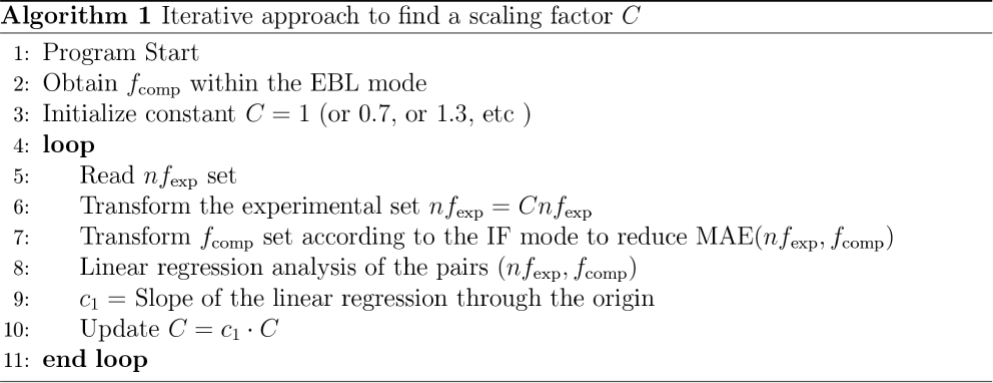


In analogy to how *f*_comp,*k*_ values are obtained from the computed *f*_*i*-0_, from the cavity-field-modified  values we obtain  either within the EBL or within the IF modes. (*f*_*i*-0_ and  correspond to the same transition energy). All the statistics presented in this subsection are equivalently obtained for the cavity-field-corrected OSs by substituting *f*_comp,*k*_ for .

When Algorithm 1 is applied to the  set, we label the resulting constant *C*^S^, while *C* is obtained for the *f*_comp_ set. If we assume that the simple cavity field correction implemented in this manuscript does not introduce a systematic error, then values of *C*^S^ ≈ 1 and *C* < 1 (because the cavity field correction is always greater than 1) would mean that an electronic structure method does not systematically overestimate nor underestimate the experimental OSs.

## Results

**Figure 3:**
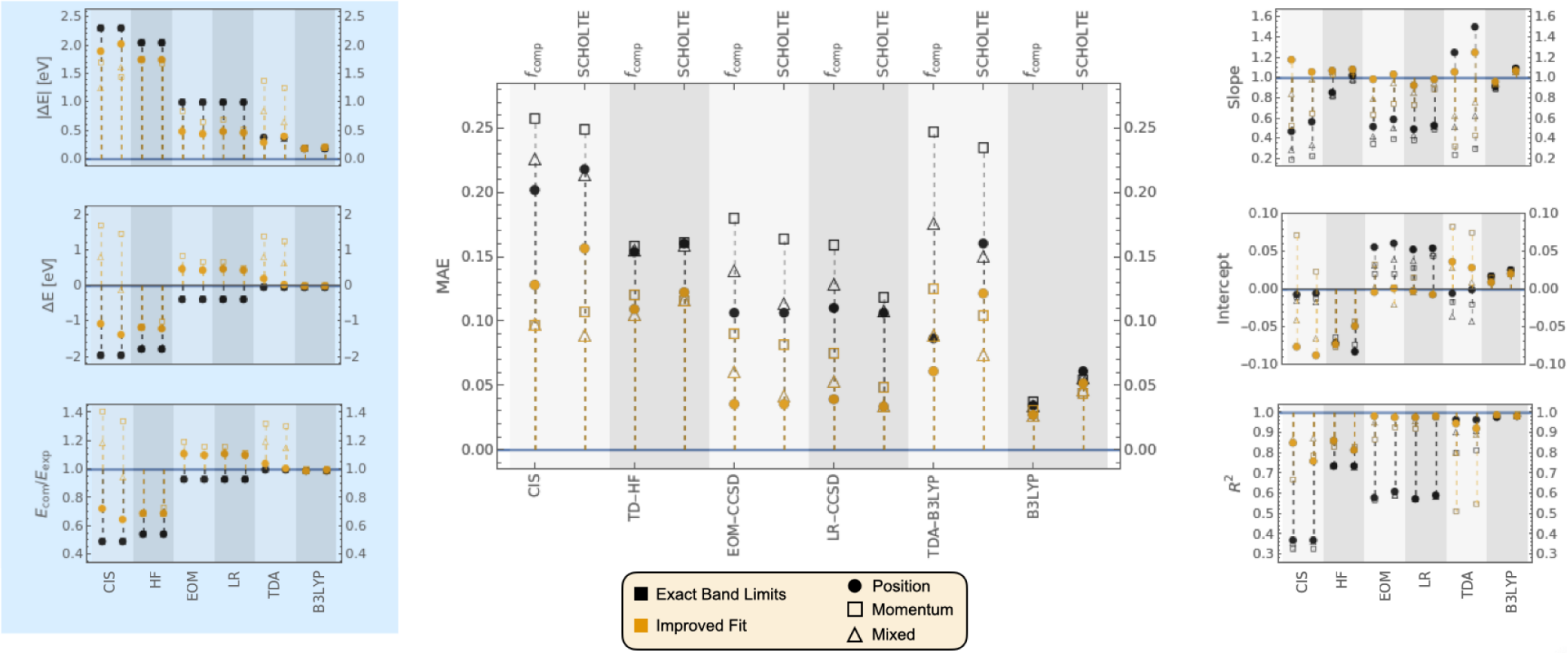
Comparison metrics for the OSs computed using CIS, TD-HF, EOM-CCSD, LR-CCSD, and B3LYP. A subset of 35 experimental transitions from the VHHM set was considered. For each method, *f*_comp_ values (left) and  values corrected with Sholte’s expression (right), are compared to *n* · *f*_exp_, as indicated by the labels on top of the central plot. Markers in black correspond to transitions assigned using the EBL mode, while markers in yellow correspond to the IF mode. A full circle corresponds to length-gauge OS values, an empty square to the velocity gauge OSs, and an empty triangle to the mixed gauge. For reference, the average OS for the set of 35 VHHM experimental transitions is ⟨*n* · *f*_exp_⟩ = 0.298506. The data displayed can be found in Tables S2–S13.

Figure 3 compares the OSs computed with the wave function methods CIS, TD-HF, EOM-CCSD, and LR-CCSD to a subset of 35 experimental transitions that stem from 26 molecules. The IDs of the transitions and the molecules they correspond to are listed in Table S1. The criteria for their selection were (i) the reliability of the experimental OS values (these molecules belong either to the medium, high, or very high confidence data in ref (1), and hence are referred to as VHHM) and (ii) the small size of the molecules to make EOM and LR-CCSD calculations feasible. The electronic structure calculations were originally reported in ref (2). Here, the cavity field correction described above is applied to the computed OSs. For comparison, B3LYP within the Tamm-Dancoff approximation (TDA) and TD-B3LYP calculations on the same set of molecules are also included.

Our CIS/6-311++G**, EOM-CCSD/aug-cc-pVDZ, LR-CCSD/aug-cc-pVDZ, and TDA-B3LYP/6–311++G** calculations in ref (2) yielded length-gauge OSs significantly larger than the velocity-gauge’s OSs, while the OSs from the mixed-gauge were in between these two. In general, the velocity-gauge’s *f*_comp_ underestimated the *f*_exp_ values and the length-gauge *f*_comp_ overestimated *f*_exp_ (see Table 2 in ref (2), or Table 3 below). The cavity field corrections applied in this manuscript are always greater than 1, since we always have that *F*_C, *k*_/*E* ≥ 1. Accordingly, the *f*_comp_ values from the velocity and mixed gauges tend to improve their agreement to *nf*_exp_ after the correction (the reduction in MAE is particularly noticeable for LR-CCSD).

**Figure 4:**
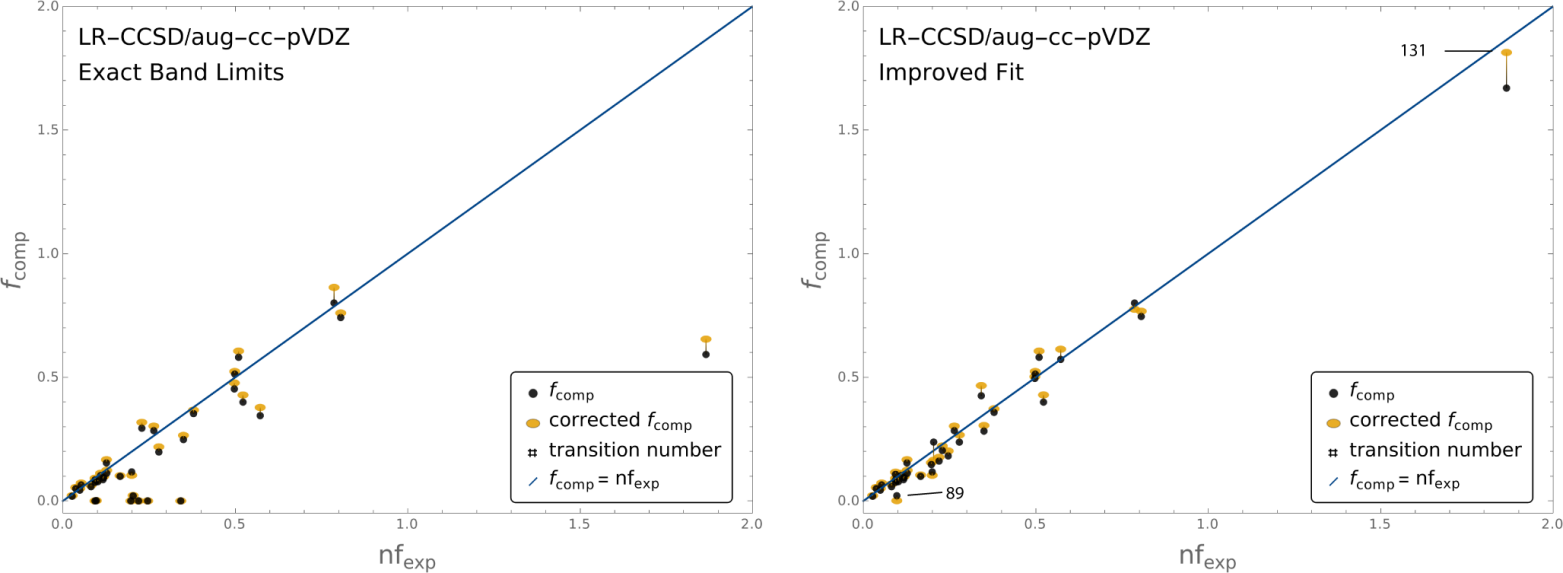
Scatter plot of the (*nf*_exp_, *f*_comp_) and (*nf*_exp_, ) pairs for the LR-CCSD/aug-cc-pVDZ calculations. Computed OS values come from the length-gauge TDMs. Black dots correspond to *f*_comp_ values without the cavity field correction. Yellow ellipses show the corresponding  values after the correction is applied. The line *f*_comp_ = *nf*_exp_ + 0 is shown in blue as a reference. Slopes, intercepts, and *R*^2^ values for the linear regression can be found in Figure 3. The left panel displays *f*_comp_ and  values obtained within the EBL mode, where the experimental band limits are strictly enforced. The right panel displays the corresponding values within the IF mode, with flexible band energy limits.

For the length-gauge, overall, there is only a slight improvement of the MAE for EOM-CCSD and LR-CCSD while for CIS and TDA-B3LYP the cavity field correction results in significantly poorer agreement. For TD-HF and TD-B3LYP, which have negligible gauge dependence, the agreement also worsens. For the length-gauge of each method, the number of individual transitions for which the cavity field correction improves agreement is reported in Table 2. In the case of LR-CCSD this effect can be seen explicitly in Figure 4 that presents scatter plots of the (*nf*_exp,*k*_, *f*_comp,*k*_) and the (*n f*_exp,*k*_, ) pairs obtained before and after applying the cavity field correction. Figure 4 presents the values obtained both in the EBL (left) in the IF (right) modes. Equivalent scatter plots for all methods, but with all the transitions labeled, are provided in the Supporting Information. Schemes of the molecular structures and the transitions from each molecule are also provided in the Supporting Information to serve as a quick reference for the labeled transitions. The Supporting Information of ref (1) contains further details for each transition, such as solvent data and energy ranges.

**Table 2:** Number of Transitions That Improve Their Agreement to Experiment with the Cavity Field Correction (i.e., Transitions for which |*f*_comp,*k*_^S^ – *nf*_exp, *k*_| is Smaller than |*f*_comp,*k*_ – *nf*_exp, *k*_|)^a^

Correction	CIS	HF	EOM-CCSD	LR-CCSD	TDA-B3LYP	TD-B3LYP
Improves	9	10	19	22	4	11
Does Not	26	25	16	13	31	24

a The length-gauge OSs obtained within the IF mode were considered.

Table 3 provides the scaling factors *C* and *C*^S^ for EOM-CCSD and LR-CCSD. These constants are obtained following Algorithm 1 (Statistical Analysis subsection). This analysis excludes transitions 89 and 131 to prevent these two from severely weighting on the results. From the IF scatter plot, it can be seen that transition 89 presents a large relative error, while transition 131 has an OS significantly larger than all other transitions (the experimental band corresponding to transition 131 groups several electronic transitions into a single band). An equivalent analysis across all 35 transitions yields very similar results (see Table S14).

**Table 3:** Scaling Factors for EOM-CCSD and LR-CCSD Excluding Transitions 89 and 131^a^

Method	*C*^S^	*C*
EOM-CCSD length gauge	1.113	1.002
EOM-CCSD momentum gauge	0.653	0.584
EOM-CCSD mixed gauge	0.902	0.762
EOM-CCSD length–momentum	0.460	0.419
LR-CCSD length gauge	0.990	0.939
LR-CCSD momentum gauge	0.884	0.664
LR-CCSD mixed gauge	0.955	0.779
LR-CCSD length–momentum	0.106	0.275

a The analysis was carried out considering 33 of the 35 transitions in the 35 VHHM set. Transition 89 was excluded since it significantly deviates from the others, while 131 was excluded to prevent its high value from biasing the analysis.

In Table 3, we see a strong dependence of the EOM-CCSD scaling factors *C* and *C*^S^ on the gauge used. As discussed in ref (2) this difference is reduced but not completely eliminated with LR-CCSD. Such a gauge dependence has been also observed by Sarkar et al.,^78^ although the gauge dependence we find here is a little larger. This could be due to our use of a smaller (double-ζ) basis set, or the different nature of our molecules (which are generally larger and more conjugated).

Importantly, we find that in all three gauges for LR-CCSD, the Scholte correction leads to an improvement in the scaling factor, with *C*^S^ ≈ 1 in the length gauge. This result, together with the number of improved molecules in Table 2 and the reduced MAE and improved linear regression metrics in Figure 3, indicate that an ellipsoidal cavity field correction systematically improves the agreement between experimental oscillator strengths and those computed with LR-CCSD.

While the results of EOM-CCSD and LR-CCSD are encouraging for the use of an ellipsoidal cavity field correction, we find that the agreement instead is worse for TD-B3LYP. This is despite the fact that TD-B3LYP was found to give one of the smallest MAEs in our previous benchmark study when compared against *n f*_exp_. This previously observed agreement may stem from some cancellation of errors. Next, we revisit the effect of the cavity field correction for several TD-DFT functionals.

Due to the lower computational cost of TD-DFT, it is possible to carry out the calculations for a larger subset of molecules. In addition to the 26 molecules considered for wave functions methods, another 43 molecules are included in the benchmark for a total of 69 molecules and 85 transitions. These represent all the OSs considered of *medium* to *very high* confidence (VHHM) when obtained in ref (1). The IDs of the transitions and the corresponding molecules can be found in Table S1.

**Figure 5:**
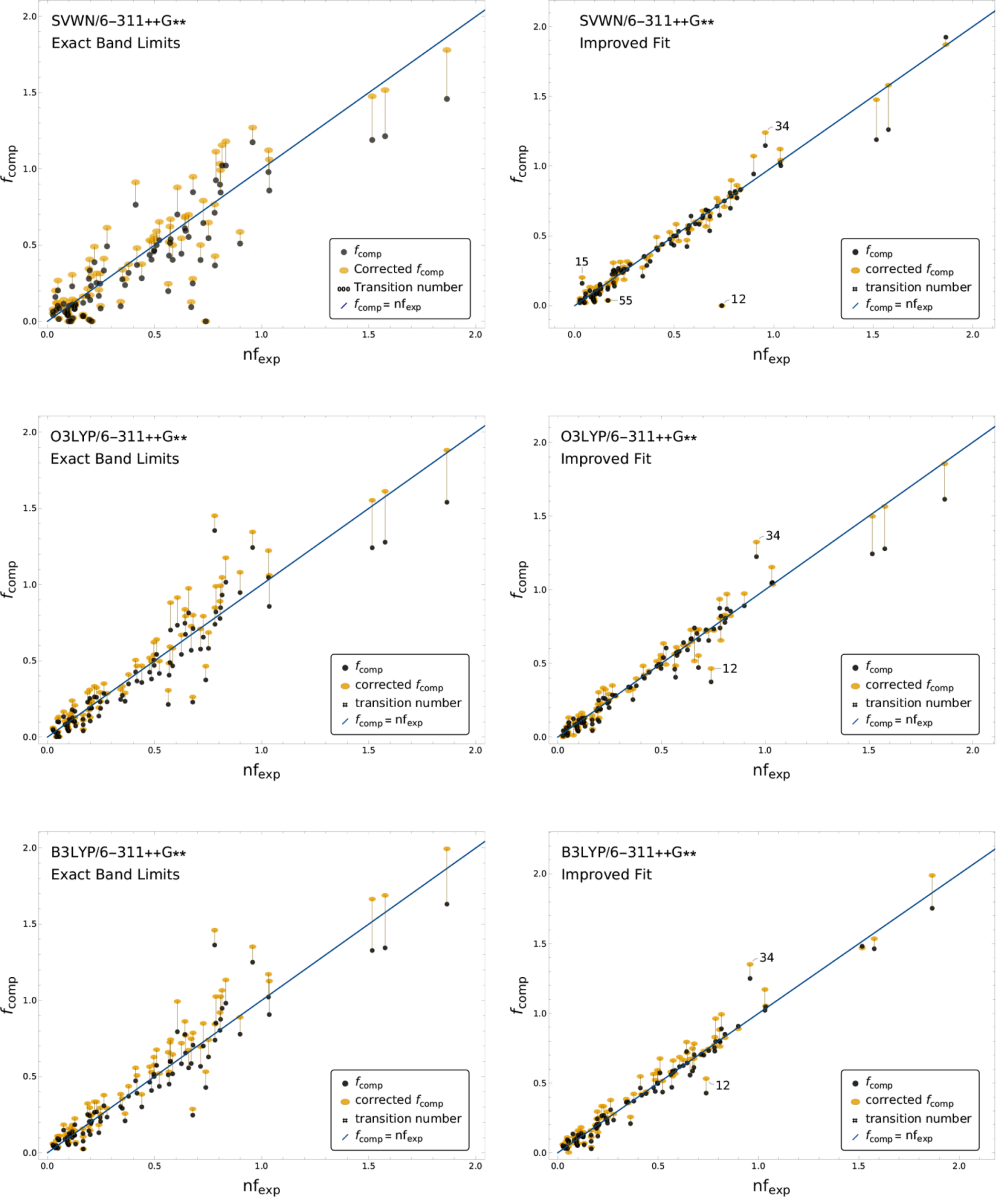
Scatter plot of the (*nf*_exp_, *f*_comp_) and (*nf*_exp_, ) pairs for SVWN, O3LYP, and B3LYP/6-311++G**. The computed values correspond to the length-gauge TDMs. Markers in black correspond to *f*_comp_ values without the cavity field correction. Markers in yellow show the corresponding  values after the correction is applied. The line *f*_comp_ = *nf*_exp_ + 0 is shown in blue as a reference. The computed values shown in the left panels were obtained within the EBL mode, where the experimental band limits are strictly enforced. The right panel displays the corresponding values within the IF mode, with flexible band energy limits.

Figure 5 presents (*nf*_exp_, *f*_comp_) and (*nf*_exp_, ) scatter plots for SVWN, O3LYP, and B3LYP. Equivalent plots for all computations in this manuscript are provided in the Supporting Information. All transitions are labeled in the Supporting Information figures.

The pure functional SVWN predicts worse energies than the hybrids O3LYP and B3LYP. Accordingly, SVWN presents a larger reorganization of the transitions when going from the EBL to the IF mode. It can also be appreciated from the scatter plots that a few transitions significantly deviate from the overall trend. For example, for SVWN in the IF mode, the two outlying transitions: 12 and 34, from molecules 011 and 027 respectively, significantly affect the overall MAE. Transition 15 from molecule 013, and 55 from 024 are other notable outliers with significant relative errors, though given their smaller OSs, these transitions contribute less to the MAE. In the scatter plots provided in the Supporting Information, it can be seen that all functionals poorly capture transition 34, while transition 12 is only captured correctly by SOGGA11-X, BHandHLYP, and the long-range corrected functionals.

Table 4 summarizes how the OSs computed with a given method scale compared to the experimental OSs. Algorithm 1 described in the Statistical Analysis subsection was used to obtain the scaling factors *C* and *C*^S^. For each method, certain transitions were identified as outliers and omitted from the analysis. These outliers are listed in Table 4. Only the length-gauge OSs were considered.

**Table 4:** Scaling Factors for All Functional Considered in This Manuscript^a^

Method	Outlier transitions	*C*^S^	*C*
BLYP	12, 15, 34, 122	0.993	0.819
N12	12, 15, 34, 35, 60, 62, 122	0.974	0.821
OLYP	12, 15, 34, 35, 60, 62, 122	0.989	0.776
PBE	12, 15, 34	0.971	0.822
SOGGA11	12, 34, 35, 60, 62, 122, 123	0.954	0.788
SVWN	12, 15, 34	0.971	0.826
SVWN5	12, 34, 122	1.010	0.837
TPSS	12, 15, 34	1.045	0.876
B3LYP	12, 34, 55	1.152	0.953
B3P86	12, 34, 55	1.125	0.957
BHandHLYP	34, 36, 55	1.299	1.108
M05	12, 34, 36, 55	1.153	0.981
mPW1PW91	12, 34, 36	1.175	0.991
O3LYP	12, 34	1.070	0.886
PBE0	12, 34, 36, 55	1.184	0.993
SOGGA11-X	34, 36	1.269	1.077
CAM-B3LYP	34, 36, 55	1.244	1.054
LC-ωHPBE	33, 34, 36, 39	1.253	1.068
ωB97X-D	34, 36, 55, 71	1.257	1.086

a The listed outlier transitions were excluded from the analysis.

**Figure 6:**
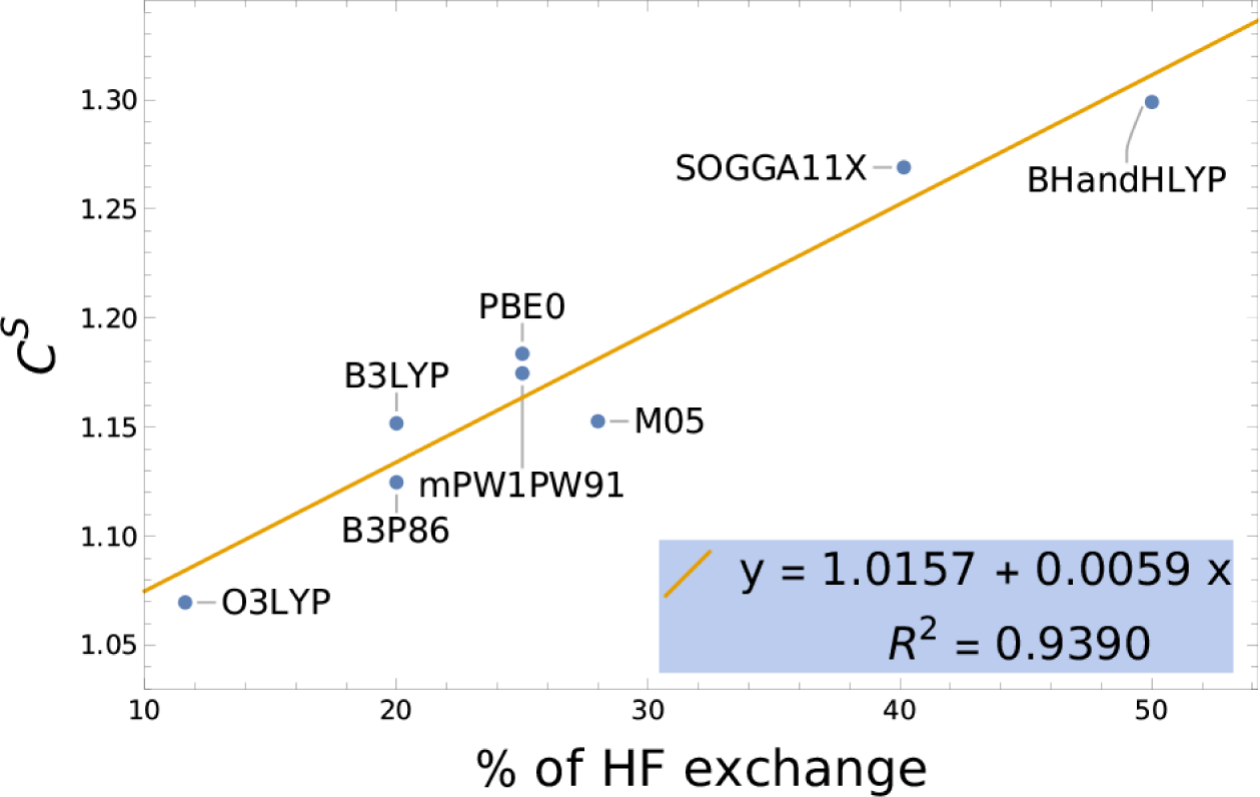
Increase of the computed OSs with the % of HF exchange in a functional.

If we neglect the limitations of the cavity model implemented in this manuscript, then a method that scales correctly with experimental OS would result in a *C*^S^ close to 1 when comparing *f*_comp_^S^ to *C*^S^*n f*_exp_, while it would result in a smaller *C* value when comparing *f*_comp_^S^ to *C n f*_exp_. The data for the pure functionals follows this trend. On the other hand, hybrid functionals present larger scaling factors, indicating that the computed OSs systematically overestimate the experimental OSs. Furthermore, the overestimation increases with the percent of HF exchange included in the functional. This is illustrated in Figure 6 where the *C*^S^ values are plotted vs the % of HF exchange in the functionals. Here, we have not studied further the effect of the range-separation parameter on oscillator strength, but we note that this has been explored to some extent in a recent study.^79^

In analogy to Table 2 for the wave function methods, Tables S63–S65 present the number of transitions that improve their agreement to *n f*_exp_ after applying the cavity field correction to pure, hybrid, and long-range corrected functionals, respectively. For the pure functionals we find that, on average, 60% of transitions benefit from the cavity field correction. For the hybrids the scenario is inverted and, on average, only 34% of transitions benefit from the correction (24% for long-range hybrids). This is aligned with the *C* ≈ 1 and *C*^S^ > 1 scaling factors found for hybrid methods. The only hybrid functional that gives similar results with and without the cavity field correction is O3LYP, which has the lowest HF exchange.

The stats for the comparison to *n f*_exp_ corresponding to the pure, hybrid, and long-range-corrected hybrid functionals are presented in Figures S24, S25, and S26, respectively. For the pure functionals,  results in consistently smaller MAEs and higher *R*^2^ values relative to *f*_comp_, though the improvement is modest. For the hybrid and long-range corrected hybrids, the stats are influenced by these method’s systematic OS overestimation. In a crude attempt to assess their performance removing the systematic overestimation, we also present stats for . The corresponding MAEs are again consistently smaller and the *R*^2^ values higher than those from *f*_comp_, but very modestly so.

All the results presented thus far for TD-DFT correspond to computations employing the 6-311++G** basis set. The calculations with aug-cc-pVTZ and aug-pcseg-2 follow the same trends, as can be seen from Table 5.

**Table 5:** Scaling Factors *C*^S^ to Minimize MAE (*C*^S^*n f*_exp_, *f*_comp_^S^) for the Pure Functional PBE and the Hybrid Functionals B3LYP, PBE0, and SOGGA11-X^a^

Method	*C*^S^/ 6–311++G**	*C*^S^/ aug-cc-pVTZ	*C*^S^/ aug-pcseg-2
PBE	1.033	0.991	0.999
B3LYP	1.158	1.127	1.113
PBE0	1.182	1.165	1.150
SOGGA11-X	1.245	1.229	1.191

a The calculations employed the 6-311++G**, aug-cc-pVTZ, and aug-pcseg-2 basis sets. The analysis was carried out across a common subset of 63 transitions. Transitions 12, 15, 25, 27, 32, 33, 34, 35, 36, 38, 39, 41, 47, 51, 55, 61, 62, 119, 121, 122, 123, and 127 were excluded from the VHHM set since they are outliers for a given method/basis set or because their calculations did not converge with aug-pcseg-2.

## Conclusions

A comparison of computed versus experimental light-absorption probability for molecules in solution needs to account for the effect of the solvent on the electromagnetic field driving the absorption. In this manuscript, we applied a model for the cavity field in which the solvent is described by a continuous dielectric inside of which an ellipsoidal cavity contains the absorbing molecule. We corrected the computed OSs accounting for the difference between the cavity field and the macroscopic field. Other approaches to account for cavity fields to OSs have been reported, for instance, in refs (3) and (80). The ellipsoidal approach used here, while simple, is relatively easy to implement and automate from readily available outputs of electronic structure calculations and should improve the agreement with the experimental OS compared to using a spherical cavity or no cavity field correction.

When the ellipsoidal cavity correction is applied to EOM-CCSD and LR-CCSD, which are the highest levels of electronic structure theory tested in this work, we find on average an improved agreement between the computed and experimental OSs by almost all metrics. However, such an agreement was not initially observed for DFT (hybrid) functionals. Upon further benchmarking of a wider range of functionals, including pure functionals (0% HF exchange) and functionals with high HF exchange (40–50%), we find that the magnitude of the computed OS is proportional to the percent of HF exchange. We also find that pure functionals, in general, give the best agreement with experimental OSs when applying the cavity field correction. Hybrid functionals generally lead to an overestimation of oscillator strengths, with BHandHLYP (50% HF exchange) overestimating experiments by (∼30%). In future OS benchmarking efforts, it is important to note the correlation between HF exchange and the OS for TD-DFT.

TD-DFT OS results reported here are in contrast to what is usually considered more accurate for the calculation of energies and other molecular properties such as molecular dipole moments and polarizabilities^52,56^ (i.e., using hybrid rather than pure functionals and moving up Jacob’s ladder). Of course, we recognize that the discovery of additional sources of computational and experimental errors may alter the conclusion that pure functionals give more reliable oscillator strengths than hybrid ones.

## Supporting Information Available

Tables with molecular structures, transition IDs, and residuals from the ellipsoidal fitting; subset of transition IDs including the VHHM sets and subset used in this work. Scatter plots for *f*_comp_ and  versus *nf*_exp_ for all methods; tables with statistics (MAE, linear regression metrics, and energy error metrics) for wave function and TD-DFT methods; figures similar to Figure 3 displaying statistics for pure, hybrid, and long-range corrected TD-DFT functionals (PDF)
A template Mathematica notebook for fitting an ellipsoid to the van der Waals surface of molecule 088 and to compute the cavity field corrections, as well as two input files containing the molecular coordinates and van der Waals surface points (ZIP)
