# Supplementary material for: Implementation of an Ellipsoidal-Cavity Field Correction for Computed Molecular Oscillator Strengths in Solution: A(nother) Benchmark Study
Source: J Chem Theory Comput. 2025 Mar 17;21(6):3120–31. doi: 10.1021/acs.jctc.5c00070 (PMC11948336; doi:10.1021/acs.jctc.5c00070)
Supplement: Supplementary file 1 — ct5c00070_si_001.pdf [file ct5c00070_si_001.pdf]

Supporting Information for manuscript: Implementation of an ellipsoidal-cavity field correction for computed molecular oscillator strengths in solution: A(nother) benchmark study

Jorge C. Garcia-Alvarez* and Samer Gozem*

Department of Chemistry, Georgia State University, Atlanta, Georgia 30302, United States

E-mail: jgarciaalvarez1@student.gsu.edu; sgozem@gsu.edu

Contents

S1 Structures and transitions **S4**

S1.0.1 Molecules: 000 - 009, Transitions: 1 - 10 . . . . S4

S1.0.2 Molecules: 010 - 019, Transitions: 11 - 24 . . . . S5

S1.0.3 Molecules: 020 - 029, Transitions: 25 - 38 . . . . S6

S1.0.4 Molecules: 030 - 039, Transitions: 39 - 51 . . . . S7

S1.0.5 Molecules: 040 - 049, Transitions: 52 - 70 . . . . S8

S1.0.6 Molecules: 050 - 059, Transitions: 71 - 92 . . . . S9

S1.0.7 Molecules: 060 - 069, Transitions: 93 - 114 . . . . S10

S1.0.8 Molecules: 070 - 079, Transitions: 115 - 129 . . . . S11

S1.0.9 Molecules: 080 - 089, Transitions: 130 - 148 . . . . S12
S1.0.10 Molecules: 090 - 099, Transitions: 149 - 164 . . . . S13

S2 Subsets of transitions to be analyzed S14

S3 $(nf_{\mathbf{exp}}, f_{\mathbf{comp}})$ and $(nf_{\mathbf{exp}}, f^{\mathbf{S}}_{\mathbf{comp}})$ scatter plots S15

S3.1 Wave-function methods S15
S3.1.1 CIS S15
S3.1.2 TD-HF S16
S3.1.3 EOM-CCSD S17
S3.1.4 LR-CCSD S18
S3.2 TD-DFT/6-311++G**, pure functionals S19
S3.2.1 BLYP S19
S3.2.2 N12 . . . . S20
S3.2.3 OLYP S21
S3.2.4 PBE S22
S3.2.5 SOGGA11 . . . . S23
S3.2.6 SVWN S24
S3.2.7 SVWN5 . . . . S25
S3.2.8 TPSS S26
S3.3 TD-DFT/6-311++G**, hybrid functionals S27
S3.3.1 B3LYP S27
S3.3.2 B3P86 . . . . S28
S3.3.3 BHandHLYP S29
S3.3.4 M05 . . . . S30
S3.3.5 mPW1PW91 . . . . S31
S3.3.6 O3LYP S32
S3.3.7 PBE0 . . . . S33

S3.3.8 SOGGA11-X S34
S3.4 TD-DFT/6-311++G**, long-range corrected hybrid functionals S35
S3.4.1 CAM-B3LYP S35
S3.4.2 LC-ωHPBE S36
S3.4.3 ωB97X-D S37

S4 Stats: $f_{\mathbf{comp}}$ and $f^{\mathbf{S}}_{\mathbf{comp}}$ compared to $n(\tilde{\nu})f_{\mathbf{exp}}$ S38
S4.1 35 VHHM for wavefunction methods S38
S4.1.1 35 VHHM for wavefunction methods: f_{comp} vs. $n(\tilde{\nu})f_{\mathrm{exp}}$ S38
S4.1.2 35 VHHM for wavefunction methods: $f^{\mathrm{S}}_{\mathrm{comp}}$ vs. $n(\tilde{\nu})f_{\mathrm{exp}}$ S39
S4.2 Scaling factors for EOM-CCSD and LR-CCSD across the full 35 VHHM set of transitions S40
S4.3 Pure functionals / 6-311++G** S41
S4.3.1 Pure functionals / 6-311++G**: f_{comp} vs $n(\tilde{\nu})f_{\mathrm{exp}}$ S41
S4.3.2 Pure functionals / 6-311++G**: $f^{\mathrm{S}}_{\mathrm{comp}}$ vs $n(\tilde{\nu})f_{\mathrm{exp}}$ S42
S4.4 Hybrid functionals / 6-311++G** S43
S4.4.1 Hybrid functionals / 6-311++G**: f_{comp} vs $n(\tilde{\nu})f_{\mathrm{exp}}$ S43
S4.4.2 Hybrid functionals / 6-311++G**: $f^{\mathrm{S}}_{\mathrm{comp}}$ vs $n(\tilde{\nu})f_{\mathrm{exp}}$ S45
S4.5 Long-range corrected functionals / 6-311++G** S46
S4.5.1 Long-range corrected functionals / 6-311++G**: f_{comp} vs $n(\tilde{\nu})f_{\mathrm{exp}}$. S46
S4.5.2 Long-range corrected functionals / 6-311++G**: $f^{\mathrm{S}}_{\mathrm{comp}}$ vs $n(\tilde{\nu})f_{\mathrm{exp}}$. S47
S4.6 Dividing the computed OSs/C^S: hybrid and long-range corrected functionals S49
S4.6.1 Hybrid functionals / 6-311++G**: $f^{\mathrm{S}}_{\mathrm{comp}}/C^S$ vs $n(\tilde{\nu})f_{\mathrm{exp}}$ S49
S4.6.2 Long-range corrected functionals / 6-311++G**: $f^{\mathrm{S}}_{\mathrm{comp}}/C^S$ vs $n(\tilde{\nu})f_{\mathrm{exp}}$ S50

S5 Plots of the stats for pure, hybrids, and long-range corrected hybrid functionals S52

S1 Structures and transitions

S1.0.1 Molecules: 000 - 009, Transitions: 1 - 10

000 R = .19<br>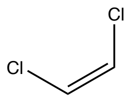<br>cis -1, 2-Dichloroethylene<br>T: 1	005 R = .26<br>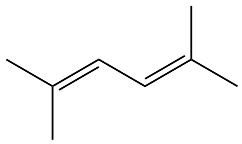<br>2,5-dimethylhexa-2,4-diene<br>T: 6
001 R = .17<br>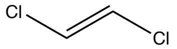<br>trans-1,2-Dichloroethylene<br>T: 2 (M)	006 R = .20<br>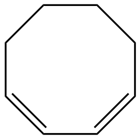<br>Cyclo-octa-1,3-diene<br>T: 7
002 R = .21<br>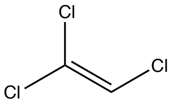<br>Trichloroethylene<br>T: 3	007 R = .25<br>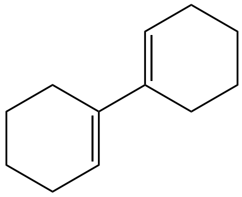<br>T: 8<br>Bi-cyclohex-1-enyl
003 R = .21<br>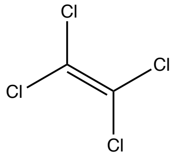<br>Tetrachloroethylene<br>T:4 (M)	008 R = .29<br>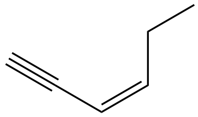<br>cis-Hex-3-en-1-yne<br>T: 9
004 R = .20<br>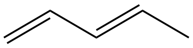<br>Penta-1,3-diene<br>T: 5	009 R = .26<br>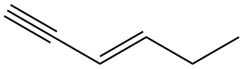<br>trans-Hex-3-en-1-yne<br>T: 10

Legend:

→ molecule ID

T: ## → transition ID

R = .## → mean absolute residual [m^{-10}] of the ellipsoid fit to the PCM surface points

S1.0.2 Molecules: 010 - 019, Transitions: 11 - 24

010 R = .22<br>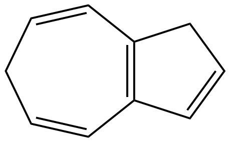<br>1,6-Dihydroazulene<br>T: 11	015 R = .18<br>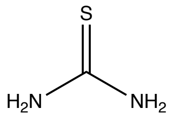<br><br>Thiourea<br>T: 19, 20
011 R = .15<br>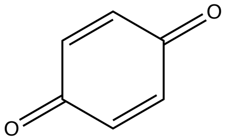<br><br>p-Benzoquinone<br>T: 12 (M)	016 R = .24<br>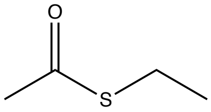<br><br>S-ethyl thioacetate<br>T: 21 (M)
012 R = .15<br>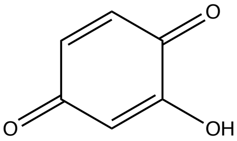<br><br>Hydroxy-p-benzoquinone<br>T: 13 (H), 14	017 R = .21<br>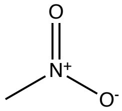<br><br>Nitromethane<br>T: 22
013 R = .21<br>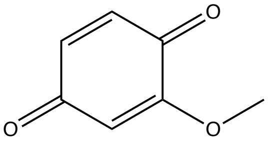<br><br>Methoxy-p-benzoquinone<br>T: 15 (H), 16 (M)	018 R = .28<br>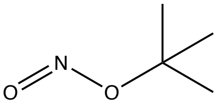<br><br>t-Butyl nitrite<br>T: 23
014 R = .21<br>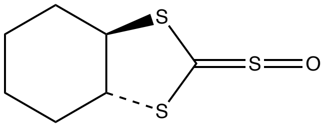<br><br>2-Sulfinylhexahydro-1,3-benzodithiole<br>T: 17, 18	019 R = .28<br>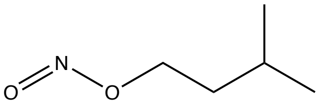<br><br>Isopentyl nitrite<br>T: 24

Legend:

→ molecule ID

T: ## → transition ID

R = .## → mean absolute residual [m^{-10}] of the ellipsoid fit to the PCM surface points

S1.0.3 Molecules: 020 - 029, Transitions: 25 - 38

020 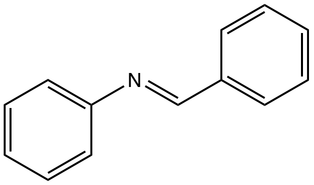 R = .28 N-Benzylideneaniline T: 25 (H)	025 R = .37 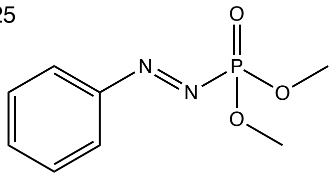 Dimethyl [(E)-phenyldiazenyl]phosphonate T: 31
021 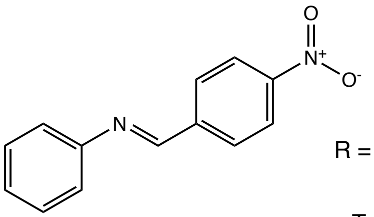 R = .27 4- Nitro-N-benzylideneaniline T: 26	026 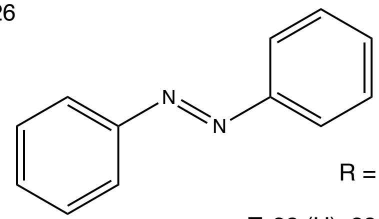 R = .21 trans-Azobenzene T: 32 (H), 33 (M)
022 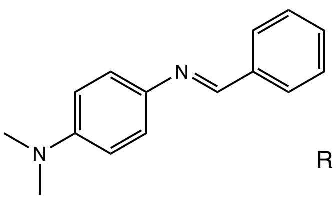 R = .27 N-Benzylidene-4-dimethylaminoaniline T: 27 H, 28	027 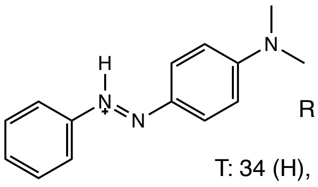 R = .23 T: 34 (H), 35 (M) 4-(Dimethylamino)azobenzene (cation)
023 R = .26 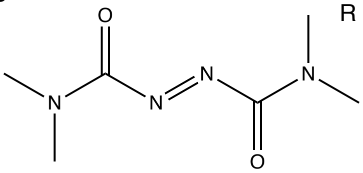 Tetramethylazodicarboxamide T: 29 (M)	028 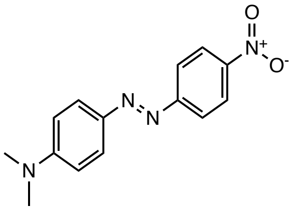 R = .23 4-Dimethylamino-4'-nitroazobenzene T: 36 (H)
024 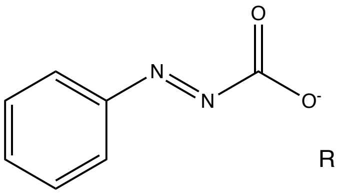 R = .28 Phenylazoformate (anion) T: 30 (M)	029 T: 37, 38 (M) R = .30 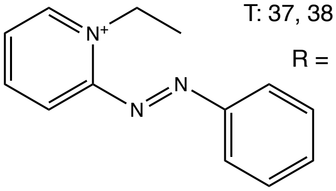 1-Ethyl-2-phenylazopyridinium (cation)

Legend:

→ molecule ID

T: ## → transition ID

R = .## → mean absolute residual [m^{-10}] of the ellipsoid fit to the PCM surface points

S1.0.4 Molecules: 030 - 039, Transitions: 39 - 51

030 1-Phenyl-2-(phenylthio)diazene<br>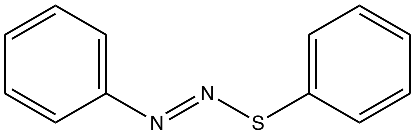<br>R = .33 T: 39 (M), 40	035 cyclohexanone semicarbazone<br>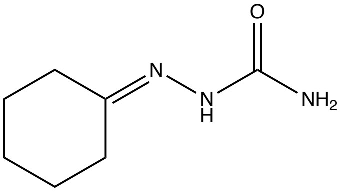<br>R = .28<br>T: 46
031<br>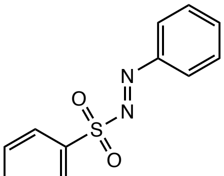<br>R = .38<br>T: 41 (M)<br>1-Phenyl-2-(phenylsulfonyl)diazene	036 1-Isopropylidene-2-p-nitrophenyl-hydrazine<br>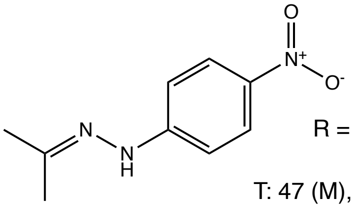<br>R = .23<br>T: 47 (M), 48
032<br>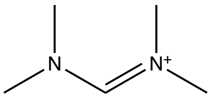<br>R = .24<br>N,N,N′,N′-Tetramethylformamidinium (cation)<br>T: 42 (M)	037<br>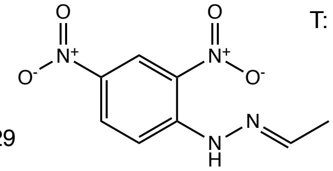<br>T: 49<br>R = .29<br>1-Ethylidene-2-(2,4-dinitrophenyl)-hydrazine
033<br>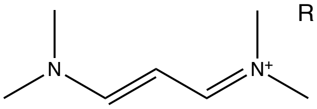<br>R = .25<br>N-[3-(Dimethylamino)allylidene]-N-methylmethanaminium (cation)<br>T: 43 (VH), 44 (M)	038<br>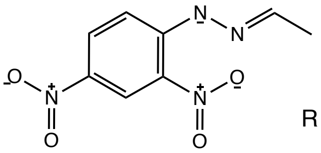<br>T: 50<br>R = .29<br>1-Ethylidene-2-(2,4-dinitrophenyl)-hydrazone (anion)
034<br>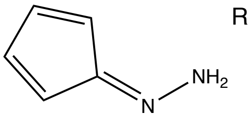<br>R = .16<br>2,4-Cyclopentadien-1-ylidenehydrazine<br>T: 45 (VH)	039<br>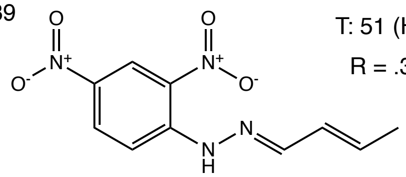<br>T: 51 (H)<br>R = .31<br>Crotonaldehyde 2,4-dinitrophenylhydrazone

Legend:

→ molecule ID

T: ## → transition ID

R = .## → mean absolute residual [m^{-10}] of the ellipsoid fit to the PCM surface points

S1.0.5 Molecules: 040 - 049, Transitions: 52 - 70

040 N,N'-Di(2-hydroxybenzylidene)hydrazine 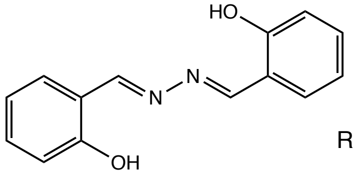 R = .22 T: 52, 53	045 T: 61 (M), 62 (M) 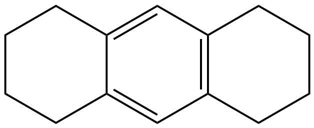 1,2,3,4,5,6,7,8-octahydroanthracene R = .24
041 R = .19 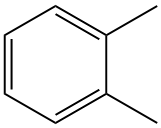 o-Xylene T: 54	046 R = .14 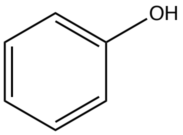 Phenol T: 63 (H), 64
042 R = .22 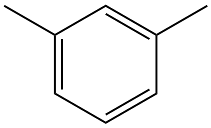 m-Xylene T: 55 (M), 56	047 R = .15 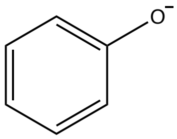 Phenolate (anion) T: 65 (H), 66
043 R = .19 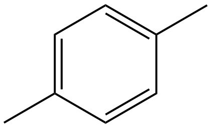 p-Xylene T: 57, 58	048 T: 67, 68 (M), 69 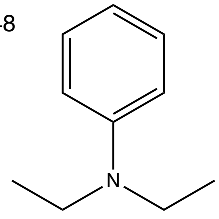 R = .27 *N*,*N*-diethylaniline
044 T: 59 (H), 60 (M) 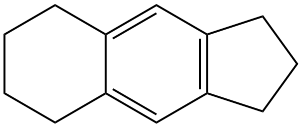 R = .24 2,3,5,6,7,8-hexahydro-1H-benz(f)indene	049 R = .15 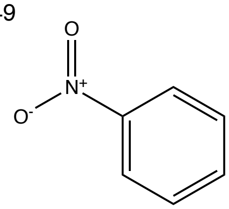 Nitrobenzene T: 70 (VH)

Legend:

→ molecule ID

T: ## → transition ID

R = .## → mean absolute residual [m^{-10}] of the ellipsoid fit to the PCM surface points

S1.0.6 Molecules: 050 - 059, Transitions: 71 - 92

050 R = .18 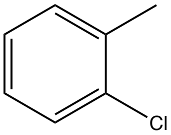 o-Chlorotoluene T: 71 (M), 72	055 T: 84 (H), 85 R = .14 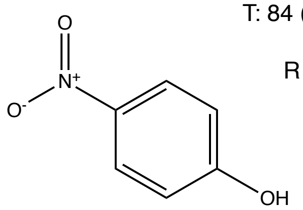 p-Nitrophenol
051 R = .18 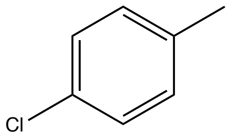 p-Chlorotoluene T: 73, 74	056 T: 86 (H) R = .14 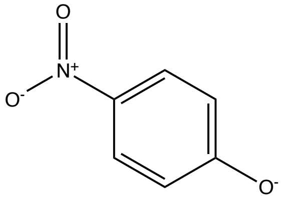 p-Nitrophenolate (anion)
052 R = .17 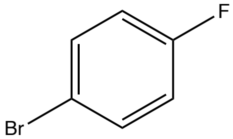 p-Bromofluorobenzene T: 75, 76, 77	057 T: 87 (VH), 88(H) 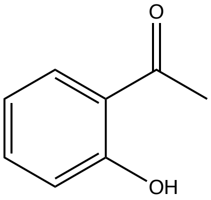 R = .18 o-Acetylphenol
053 T: 78 (M), 79 (H) 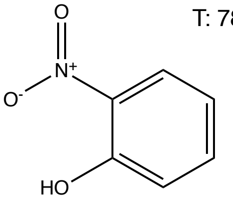 R = .15 o-Nitrophenol	058 T: 89 (M), 90 (H) 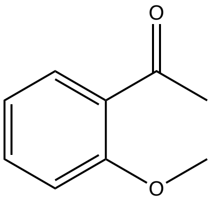 R = .25 o-Methoxyacetophenone
054 T: 80, 81 (M), 82, 83 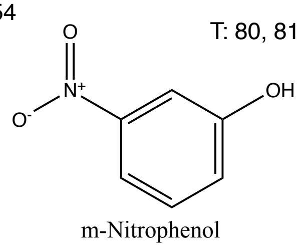 R = .16 m-Nitrophenol	059 T: 91 (VH), 92 (H) 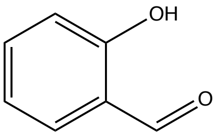 R = .15 o-Hydroxybenzaldehyde

Legend:

→ molecule ID

T: ## → transition ID

R = .## → mean absolute residual [m^{-10}] of the ellipsoid fit to the PCM surface points

S1.0.7 Molecules: 060 - 069, Transitions: 93 - 114

060 T: 93 (H), 94 (H) 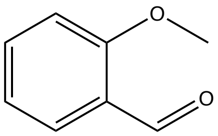 o-Methoxybenzaldehyde R = .22	065 T: 104, 105 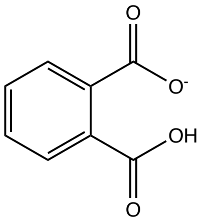 R = .20 Hydrogen phthalate (anion)
061 T: 95 (VH), 96, 97 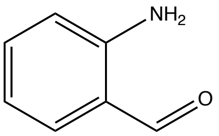 o-Aminobenzaldehyde R = .15	066 T: 106 (VH), 107 R = .17 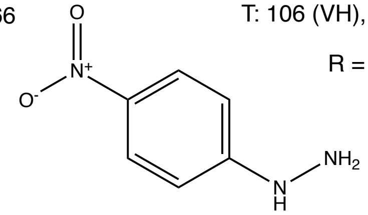 p-Nitrophenylhydrazine
062 T: 98 (VH), 99 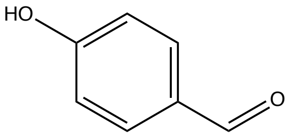 p-Hydroxybenzaldehyde R = .14	067 T: 108, 109 (M), 110 R = .19 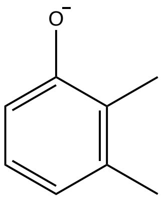 2,3-Dimethylphenolate (anion)
063 T: 100 (H), 101 (M) R = .20 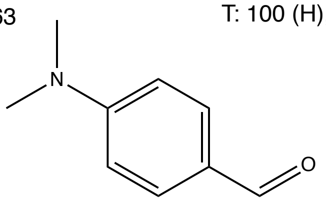 p-Dimethylaminobenzaldehyde	068 T: 111 (H), 112, 113 (M) 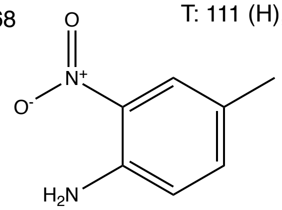 R = .20 4-Methyl-2-nitroaniline
064 T: 102, 103 (M) R = .16 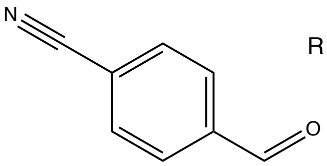 p-Cyanobenzaldehyde	069 T: 114 (VH) R = .19 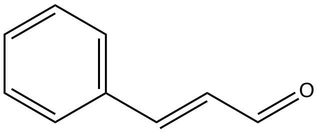 Cinnamaldehyde

Legend:

→ molecule ID

T: ## → transition ID

R = .## → mean absolute residual $[m^{-10}]$ of the ellipsoid fit to the PCM surface points

S1.0.8 Molecules: 070 - 079, Transitions: 115 - 129

070 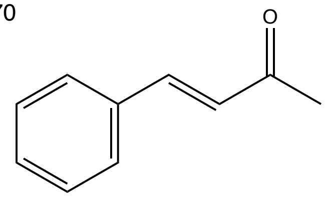 Benzylideneacetone; R = .21; T: 115 (VH), 116	075 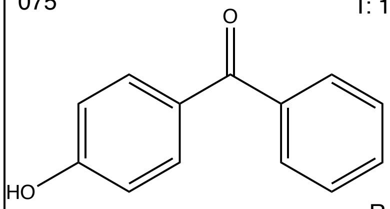 4-Hydroxybenzophenone; T: 123 (H); R = .28
071 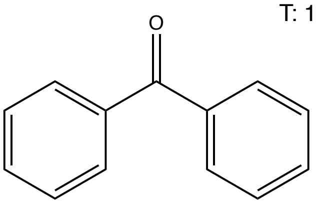 Benzophenone; T: 117 (M); R = .29	076 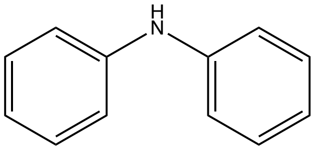 Diphenylamine; T: 124 (M); R = .26
072 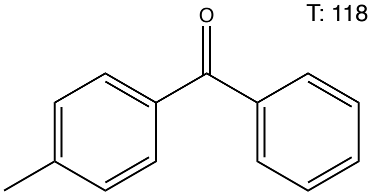 4-Methylbenzophenone; T: 118 (M); R = .29	077 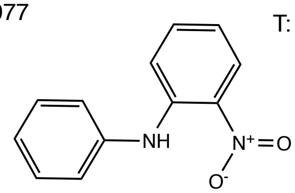 2-Nitrodiphenylamine; T: 125 (H), 126; R = .32
073 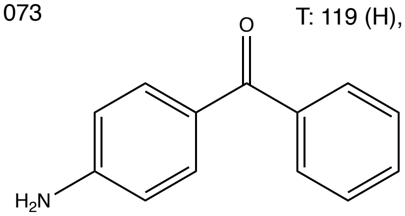 4-Aminobenzophenone; T: 119 (H), 120; R = .29	078 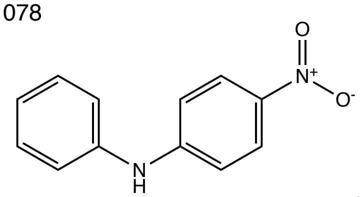 4-Nitrodiphenylamine; R = .28; T: 127 (M), 128
074 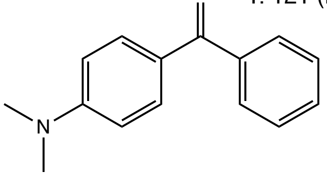 4-(N,N-Dimethylamino)-benzophenone; T: 121 (H), 122 (M); R = .31	079 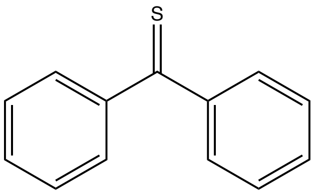 Thiobenzophenone; R = .31; T: 129

Legend:

→ molecule ID

T: ## → transition ID

R = .## → mean absolute residual [m^{-10}] of the ellipsoid fit to the PCM surface points

S1.0.9 Molecules: 080 - 089, Transitions: 130 - 148

080 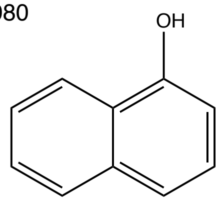 1-Naphthol<br>R = .15<br>T: 130 (H), 131 (M)	085 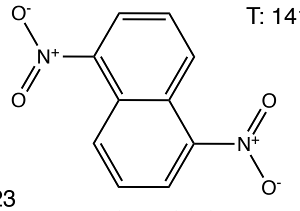 1,5-Dinitronaphthalene<br>T: 141, 142<br>R = .23
081 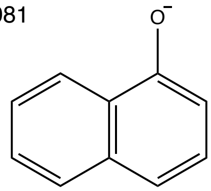 1-Naphtholate (anion)<br>R = .15<br>T: 132, 133	086 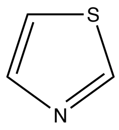 Thiazole<br>R = .14<br>T: 143
082 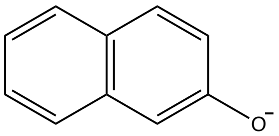 2-Naphtholate (anion)<br>T: 134 (M), 135, 136<br>R = .14	087 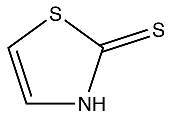 2-Mercaptothiazole<br>T: 144 (VH)<br>R = .18
083 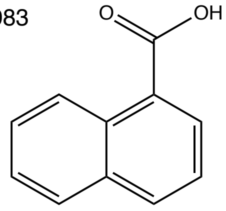 1-Naphthoic acid<br>R = .23<br>T: 137 (H), 138	088 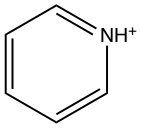 Pyridinium (cation)<br>T: 145 (VH), 146<br>R = .14
084 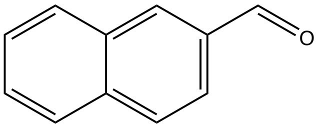 2-Naphthaldehyde<br>R = .16<br>T: 139, 140	089 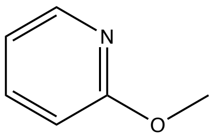 2-Methoxypyridine<br>T: 147 (VH), 148<br>R = .20

Legend:

→ molecule ID

T: ## → transition ID

R = .## → mean absolute residual [m^{-10}] of the ellipsoid fit to the PCM surface points

S1.0.10 Molecules: 090 - 099, Transitions: 149 - 164

090 T: 149 (VH), 150 (M) R = .27 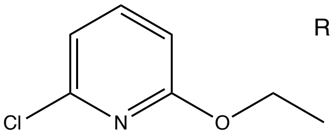 2-Chloro-6-ethoxypyridine	095 T: 156, 157 R = .16 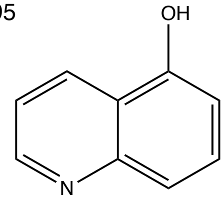 5-Hydroxyquinoline
091 T: 151(M) R = .21 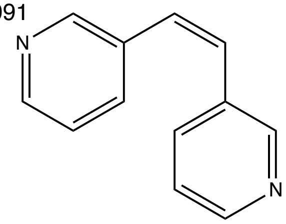 cis-1,2-Di-3'-pyridylethylene	096 T: 158 (M) R = .16 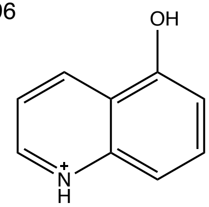 5-Hydroxyquinolynium (cation)
092 T: 152 (H) R = .15 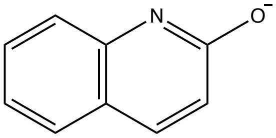 Deprotonated 2-Hydroxyquinoline (anion)	097 T: 159 (M), 160 R = .15 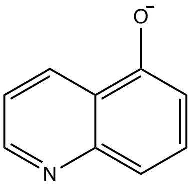 Deprotonated 5-Hydroxyquinoline (anion)
093 T: 153 (H), 154 R = .15 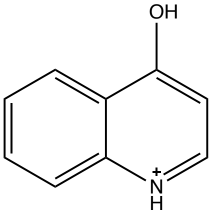 4-Hydroxyquinolynium (cation)	098 R = .16 T: 161 (M), 162 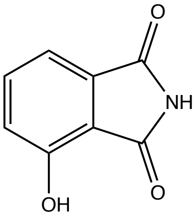 3-Hydroxyphthalamide
094 T: 155 (M) R = .15 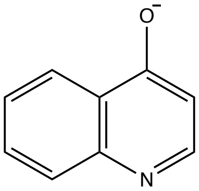 Deprotonated 4-Hydroxyquinoline (anion)	099 T: 163, 164 R = .19 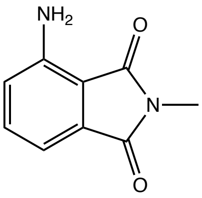 3-Amino-1-methylphthalamide

Legend:

→ molecule ID

T: ## → transition ID

R = .## → mean absolute residual $[m^{-10}]$ of the ellipsoid fit to the PCM surface points

S2 Subsets of transitions to be analyzed

Table S1: Subset of experimental transition ID numbers and molecule ID numbers used in this work. The full set and corresponding ID numbers can be found in Tarleton et al., J. Phys. Chem. A 2022, 126, 3, 435–443.

Category	Molecule IDs	Transition IDs
VHHM	2, 11, 12, 13, 16, 20, 22, 23, 24, 26, 27, 28, 29, 30, 31, 32, 33, 34, 36, 37, 39, 4, 42, 44, 45, 46, 47, 48, 49, 50, 53, 54, 55, 56, 57, 58, 59, 60, 61, 62, 63, 64, 66, 67, 68, 69, 70, 71, 72, 73, 74, 75, 76, 77, 78, 80, 82, 83, 87, 88, 89, 90, 91, 92, 93, 94, 96, 97, 98 (N = 69)	3, 5, 12, 13, 15, 16, 21, 25, 27, 29, 30, 32, 33, 34, 35, 36, 38, 39, 41, 42, 43, 44, 45, 47, 49, 51, 55, 59, 60, 61, 62, 63, 65, 68, 70, 71, 78, 79, 81, 84, 86, 87, 88, 89, 90, 91, 92, 93, 94, 95, 98, 100, 101, 103, 106, 109, 111, 113, 114, 115, 117, 118, 119, 121, 122, 123, 124, 125, 127, 130, 131, 134, 137, 144, 145, 147, 149, 150, 151, 152, 153, 155, 158, 159, 161 (N = 85)
Subset used to benchmark wavefunction methods	12, 13, 34, 46, 47, 49, 53, 55, 56, 57, 58, 59, 60, 61, 62, 66, 68, 69, 70, 80, 87, 88, 89, 90, 92, 93 (N = 26)	13, 15, 16, 45, 63, 65, 70, 78, 79, 84, 86, 87, 88, 89, 90, 91, 92, 93, 94, 95, 98, 106, 111, 113, 114, 115, 130, 131, 144, 145, 147, 149, 150, 152, 153 (N = 35)

S3 $(nf_{\mathbf{exp}}, f_{\mathbf{comp}})$ and $(nf_{\mathbf{exp}}, f^{\mathrm{S}}_{\mathbf{comp}})$ scatter plots

S3.1 Wave-function methods

S3.1.1 CIS

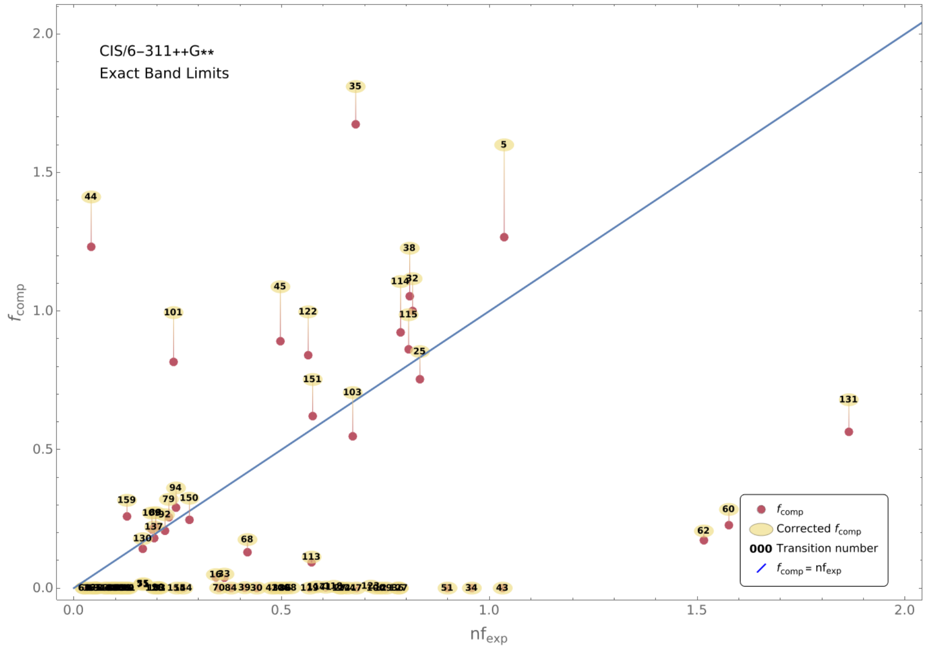

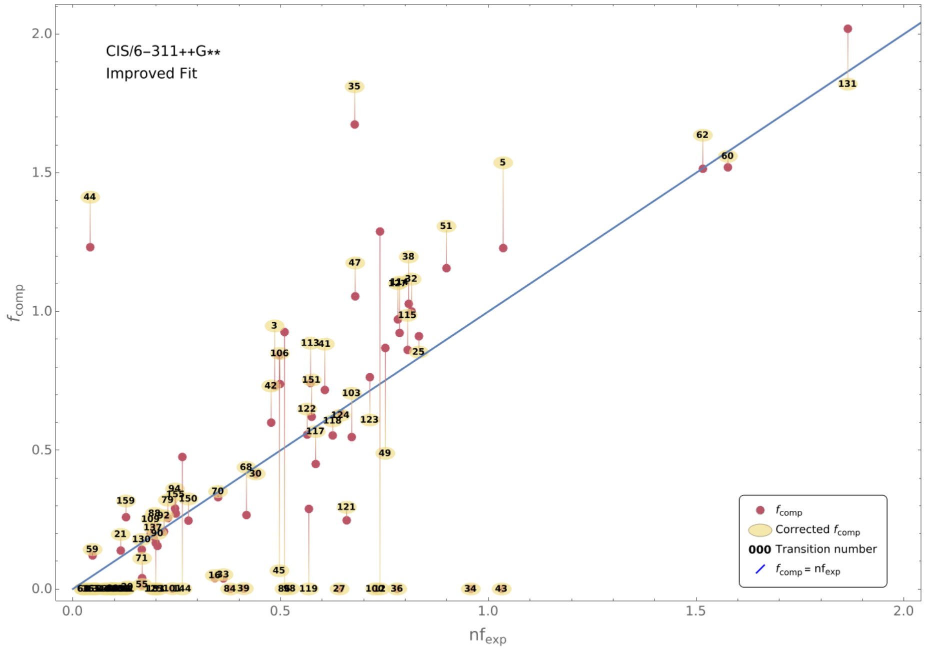

S3.1.2 TD-HF

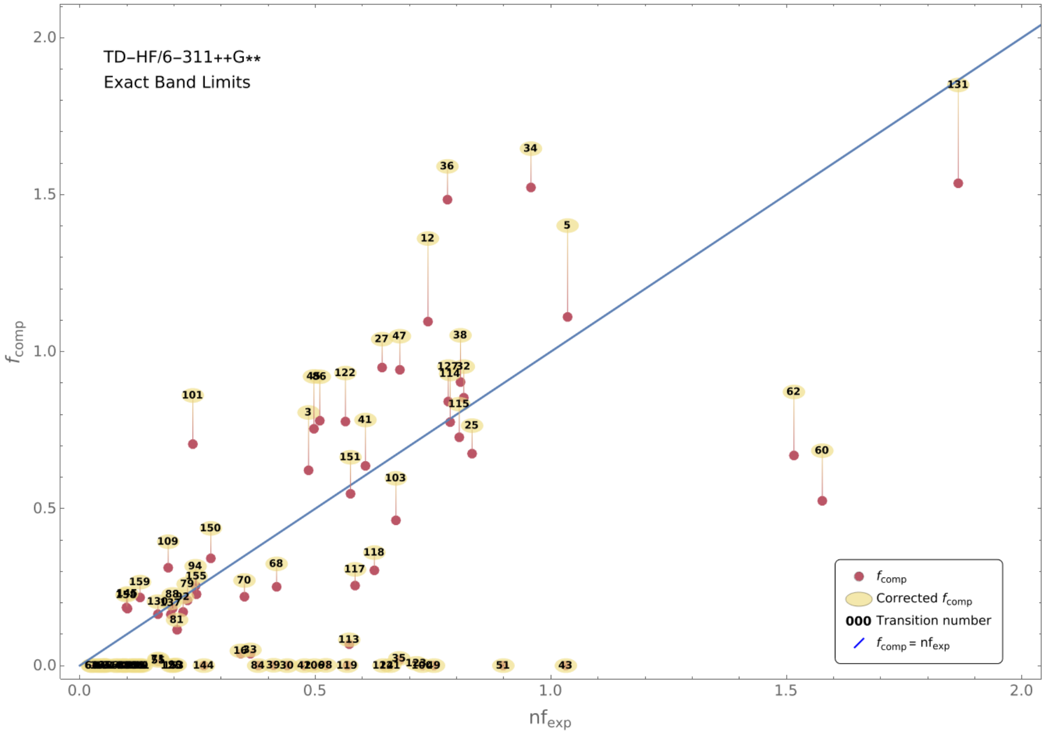
TD–HF/6–311++G**
Exact Band Limits
f_{comp}
nf_{exp}
0.0
0.5
1.0
1.5
2.0
f_{comp}
Corrected f_{comp}
000 Transition number
$f_{comp} = nf_{exp}$

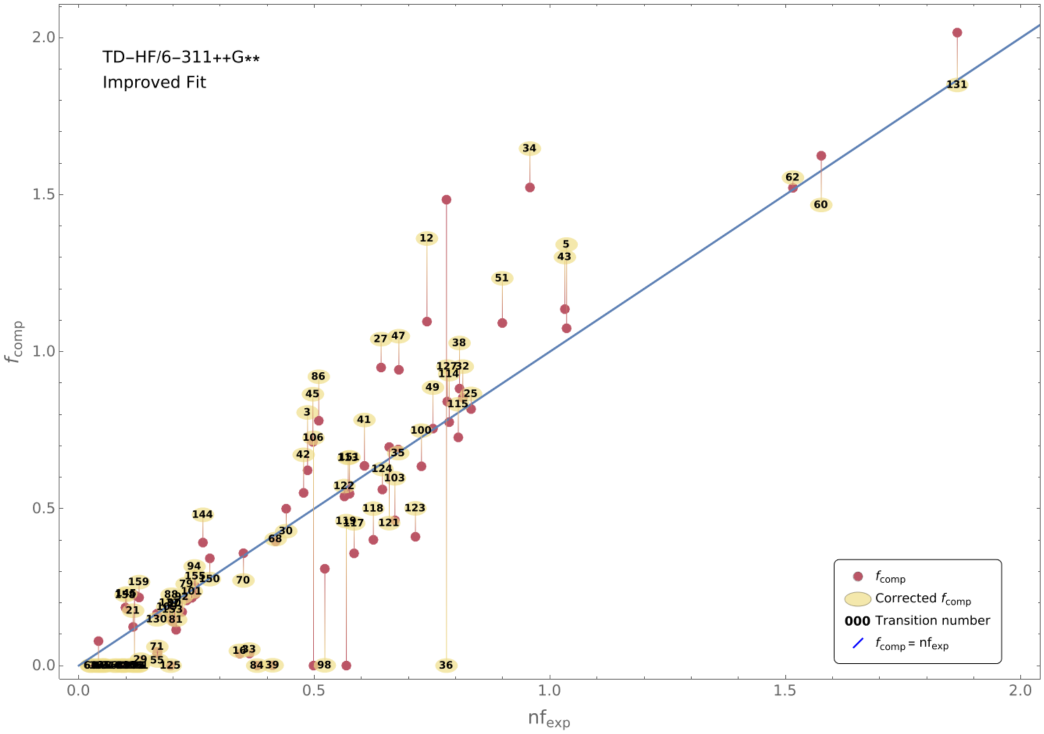
TD–HF/6–311++G**
Improved Fit
f_{comp}
nf_{exp}
0.0
0.5
1.0
1.5
2.0
f_{comp}
Corrected f_{comp}
000 Transition number
$f_{comp} = nf_{exp}$

S3.1.3 EOM-CCSD

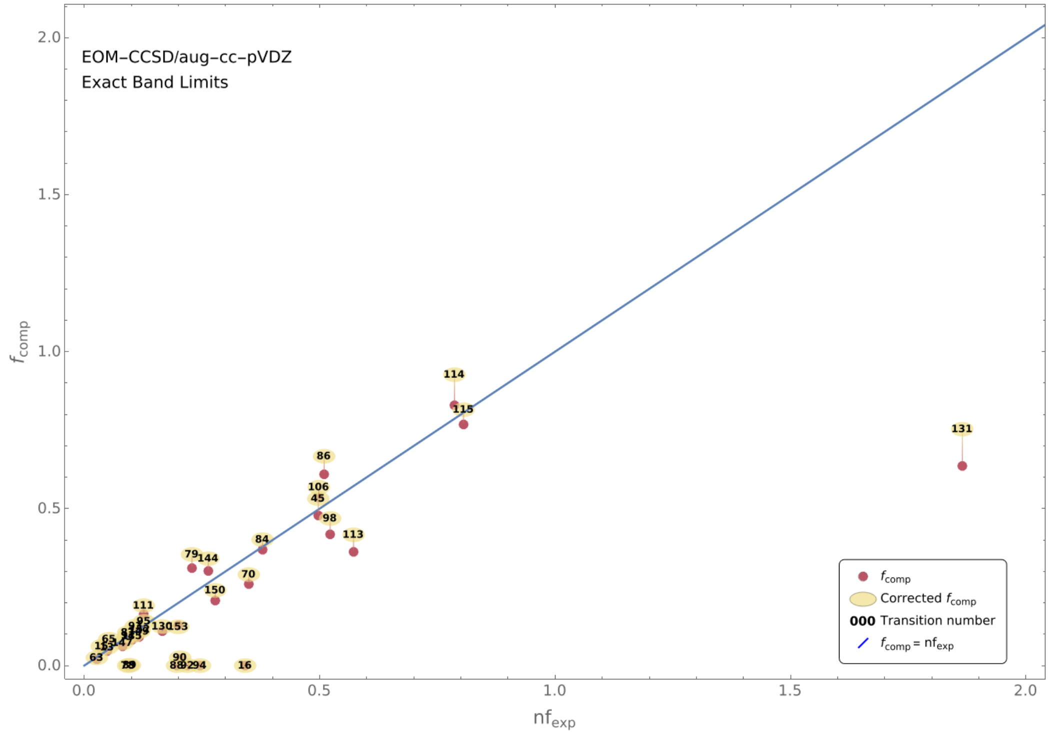

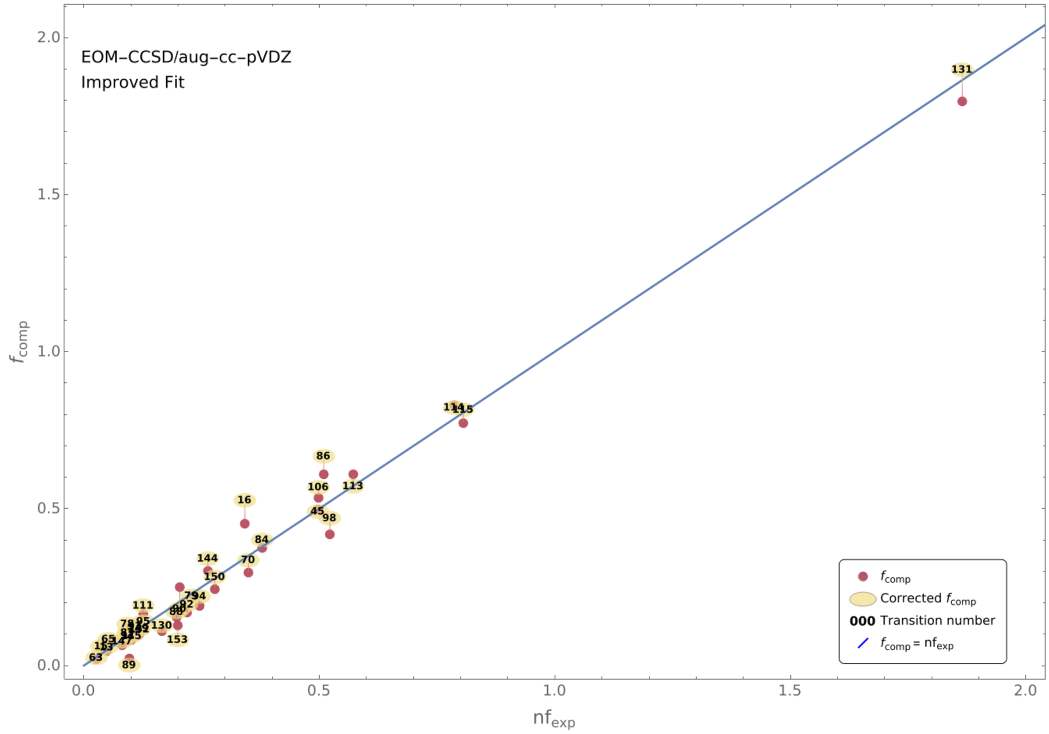

S3.1.4 LR-CCSD

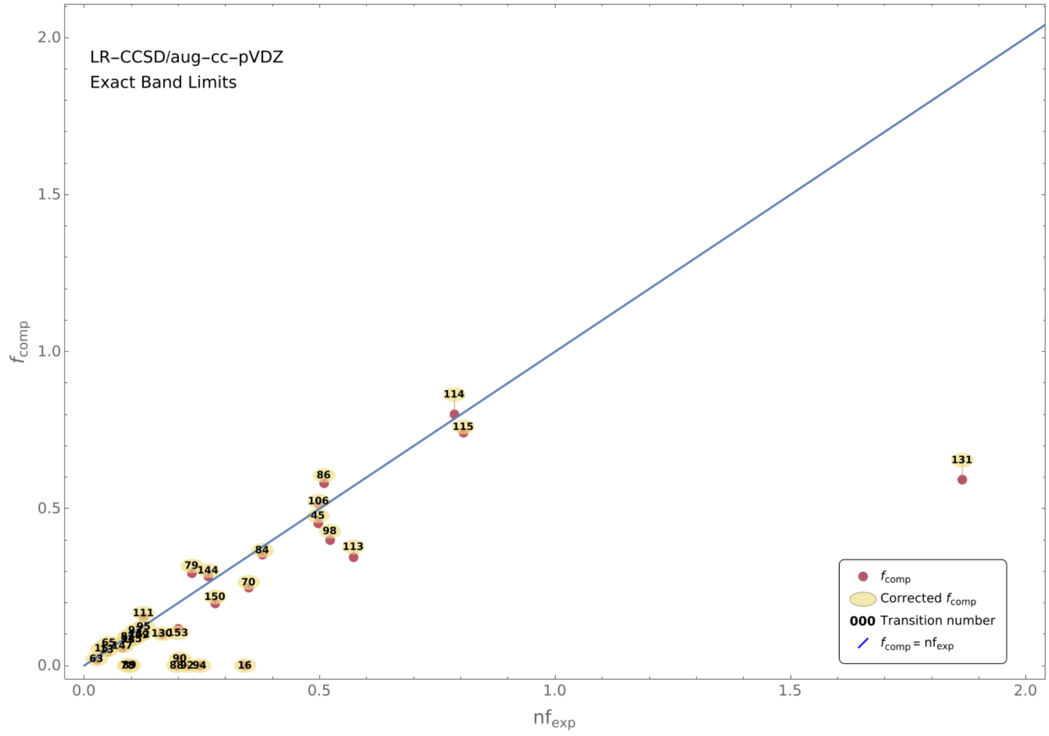
LR–CCSD/aug–cc–pVDZ
Exact Band Limits
f_{comp}
nf_{exp}
f_{comp}
Corrected f_{comp}
000 Transition number
$f_{comp} = nf_{exp}$

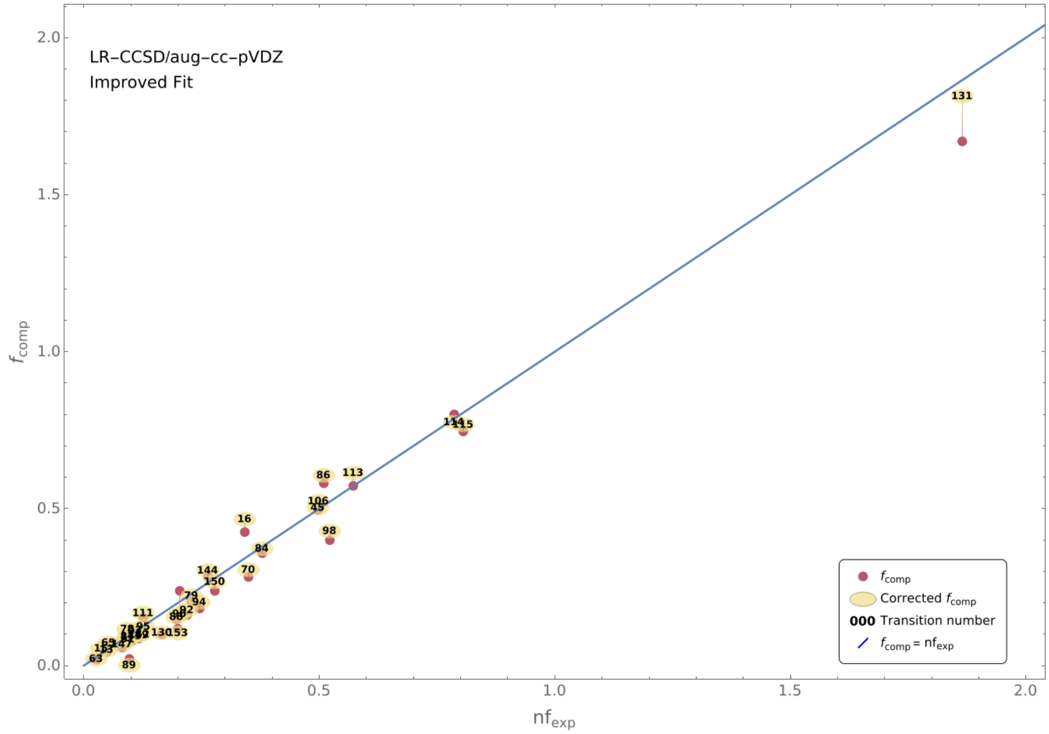
LR–CCSD/aug–cc–pVDZ
Improved Fit
f_{comp}
nf_{exp}
f_{comp}
Corrected f_{comp}
000 Transition number
$f_{comp} = nf_{exp}$

S3.2 TD-DFT/6-311++G**, pure functionals

S3.2.1 BLYP

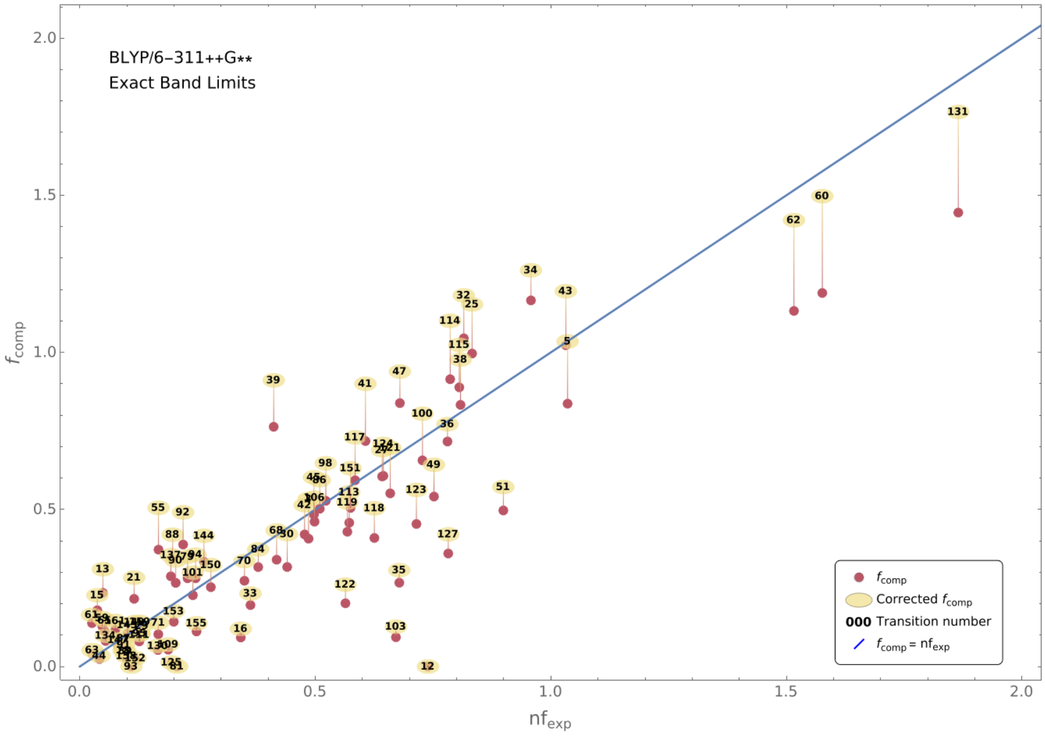

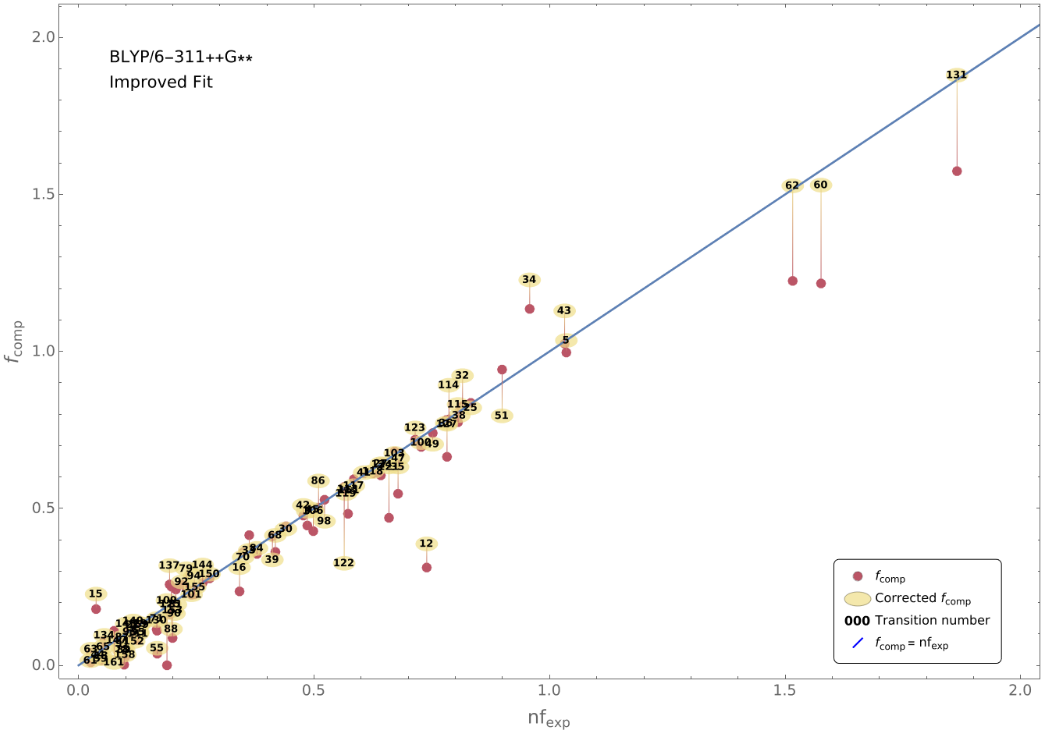

S3.2.2 N12

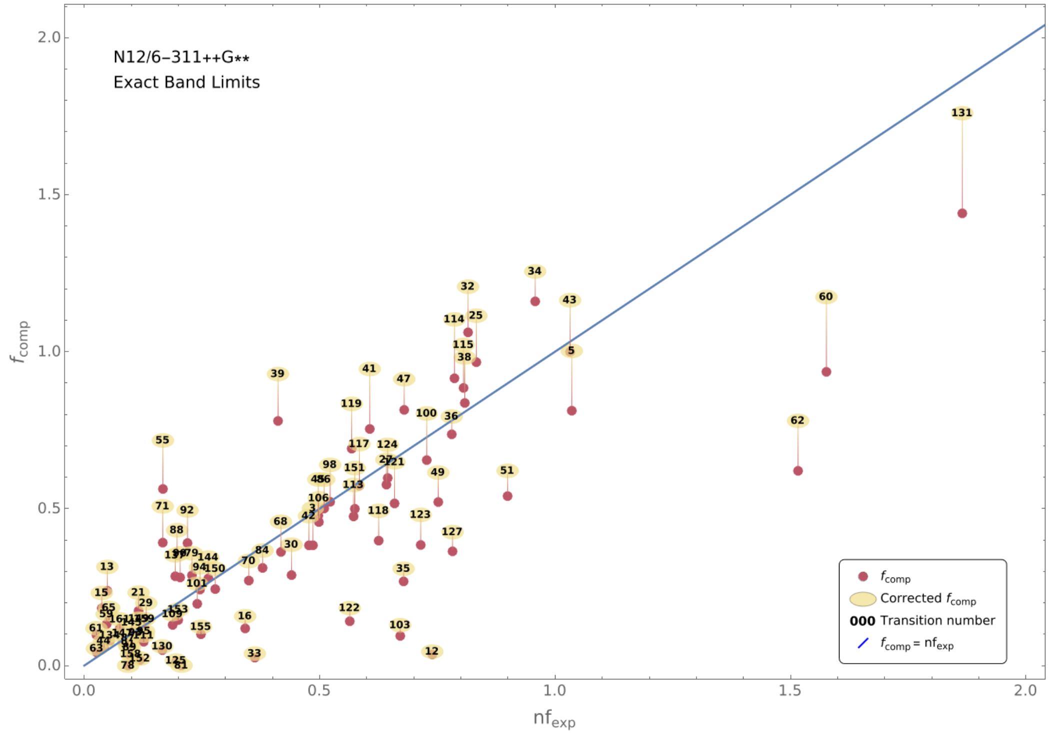
N12/6–311++G**
Exact Band Limits
f_{comp}
nf_{exp}
f_{comp}
Corrected f_{comp}
000 Transition number
$f_{comp} = nf_{exp}$

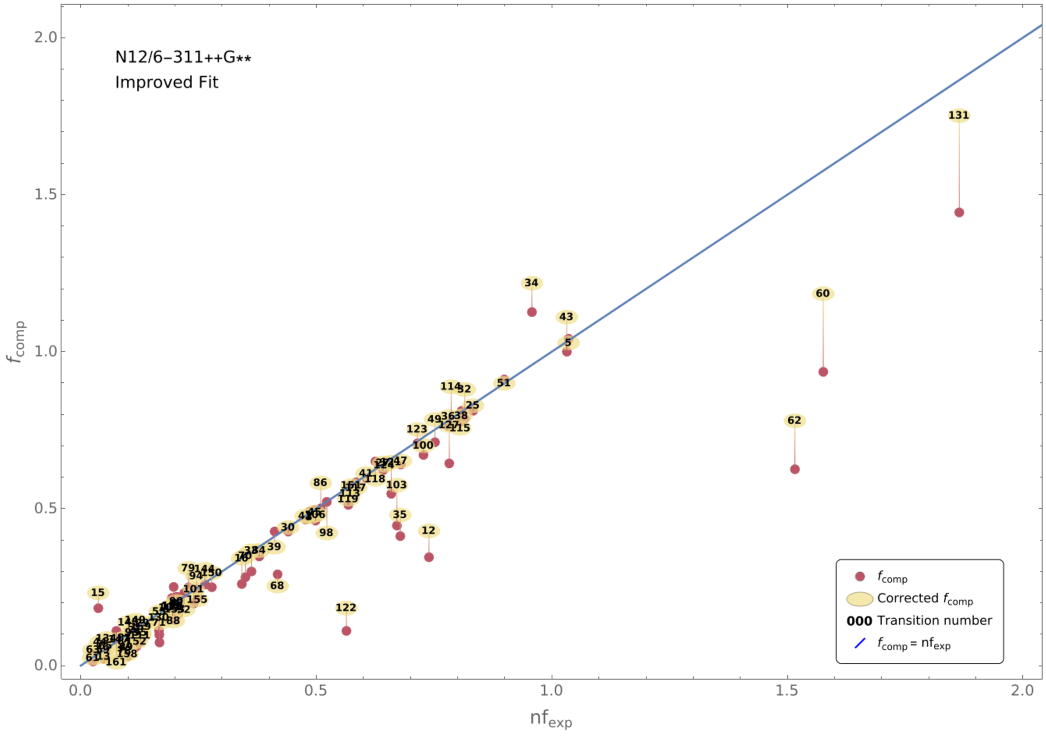
N12/6–311++G**
Improved Fit
f_{comp}
nf_{exp}
f_{comp}
Corrected f_{comp}
000 Transition number
$f_{comp} = nf_{exp}$

S3.2.3 OLYP

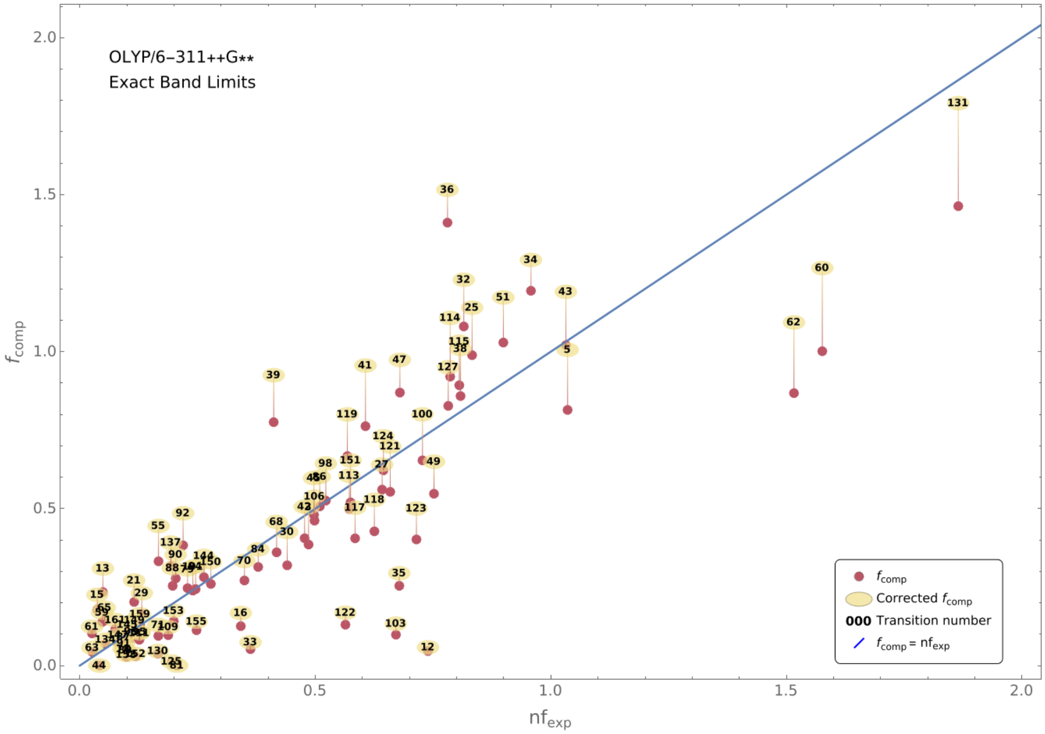

OLYP/6–311++G**
Exact Band Limits
f_{comp}
nf_{exp}
0.0
0.5
1.0
1.5
2.0
f_{comp}
Corrected f_{comp}
000 Transition number
$f_{comp} = nf_{exp}$

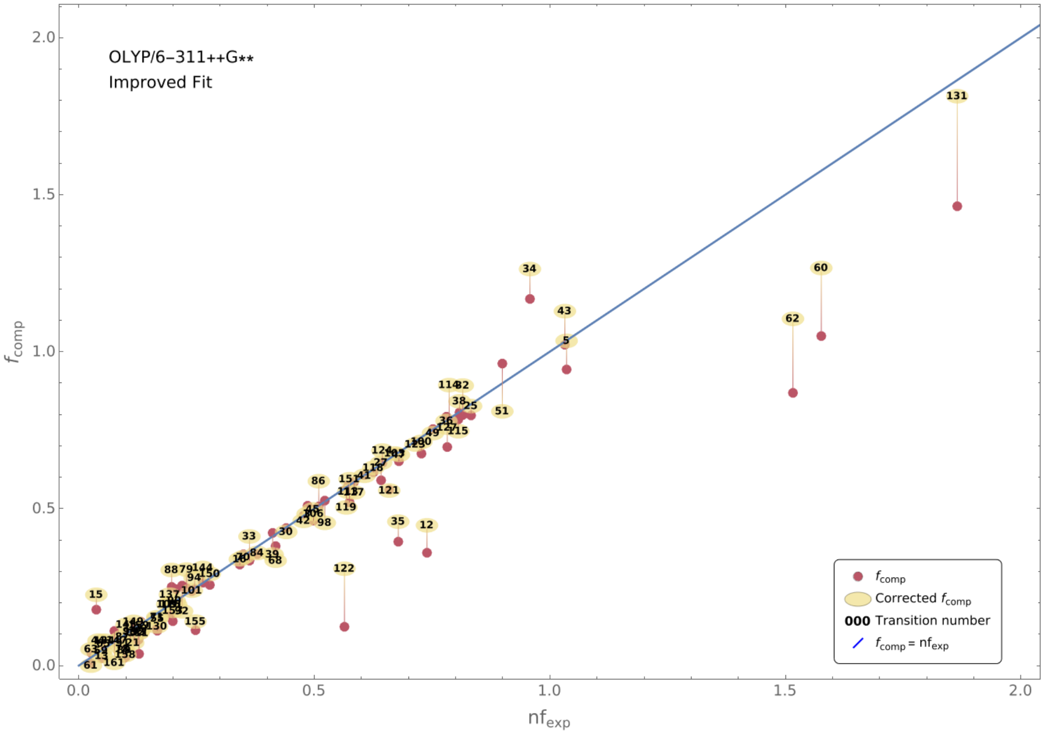

OLYP/6–311++G**
Improved Fit
f_{comp}
nf_{exp}
0.0
0.5
1.0
1.5
2.0
f_{comp}
Corrected f_{comp}
000 Transition number
$f_{comp} = nf_{exp}$

S3.2.4 PBE

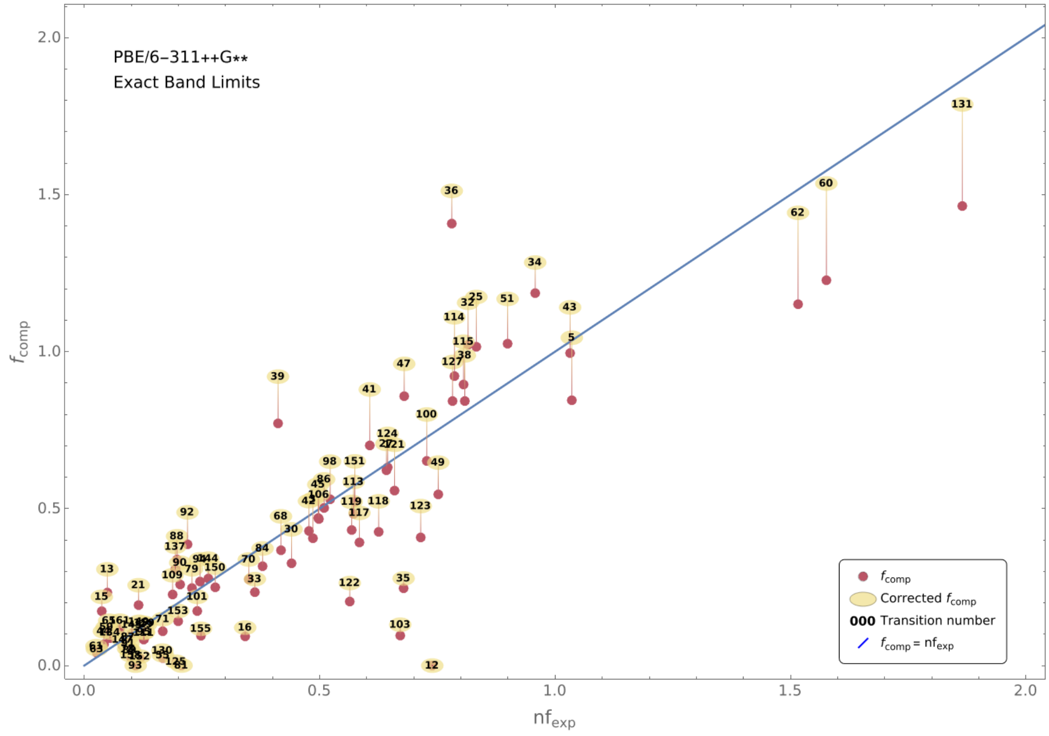

PBE/6–311++G**
Exact Band Limits
f_{comp}
Corrected f_{comp}
000 Transition number
$f_{comp} = nf_{exp}$
nf_{exp}

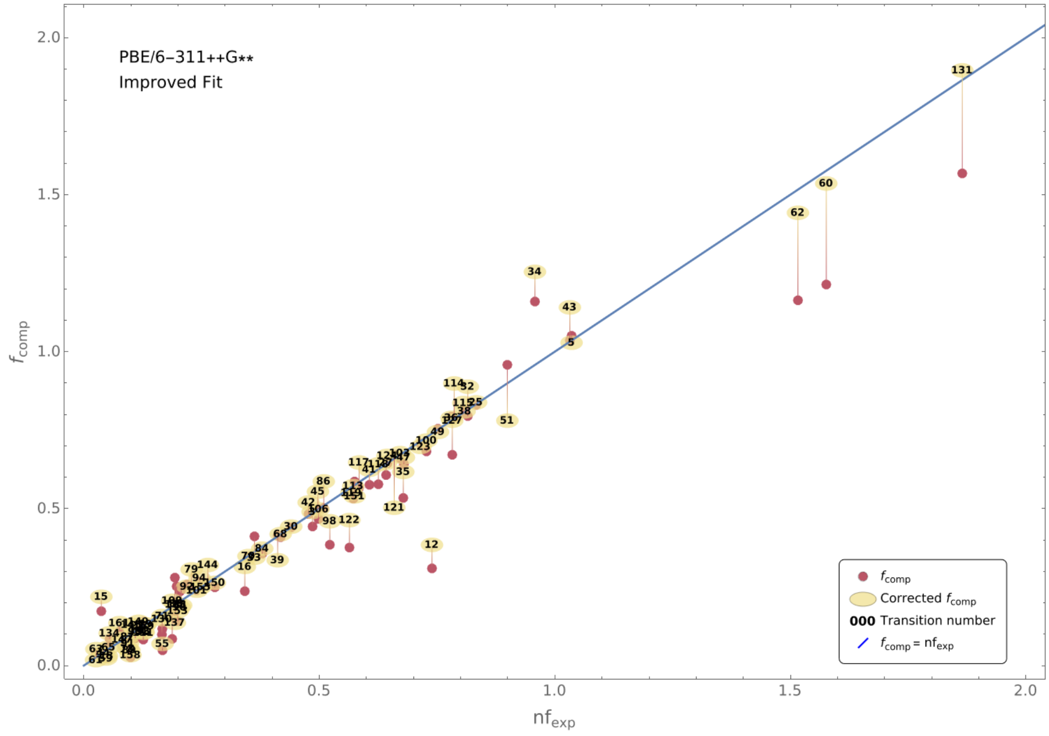

PBE/6–311++G**
Improved Fit
f_{comp}
Corrected f_{comp}
000 Transition number
$f_{comp} = nf_{exp}$
nf_{exp}

S3.2.5 SOGGA11

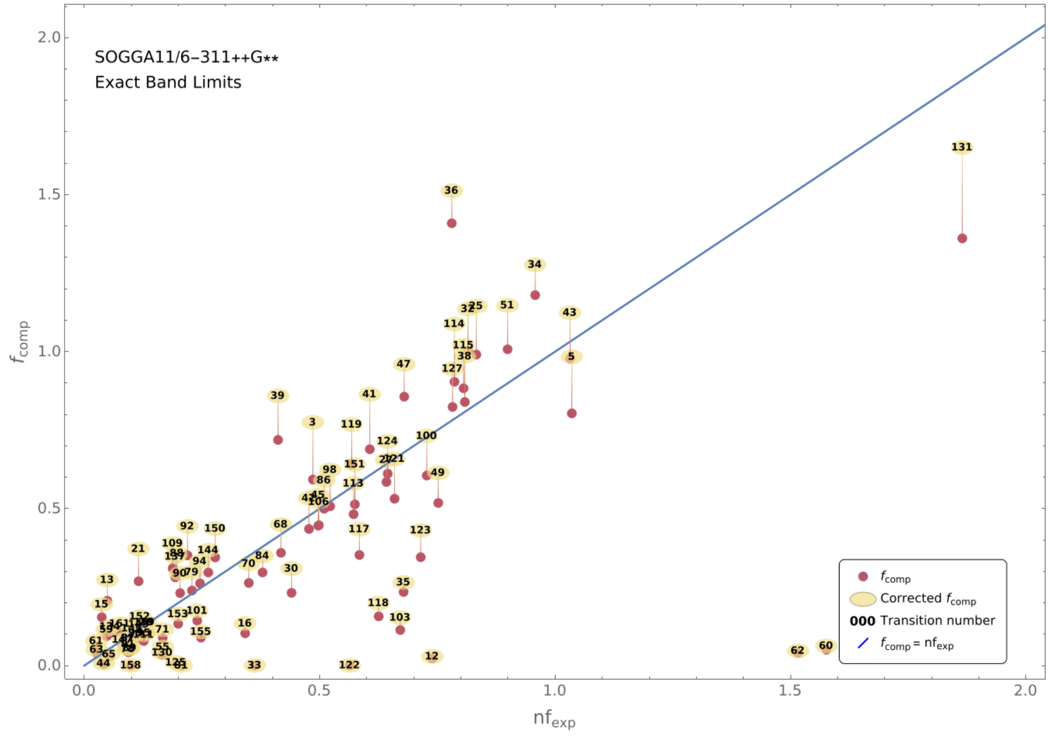

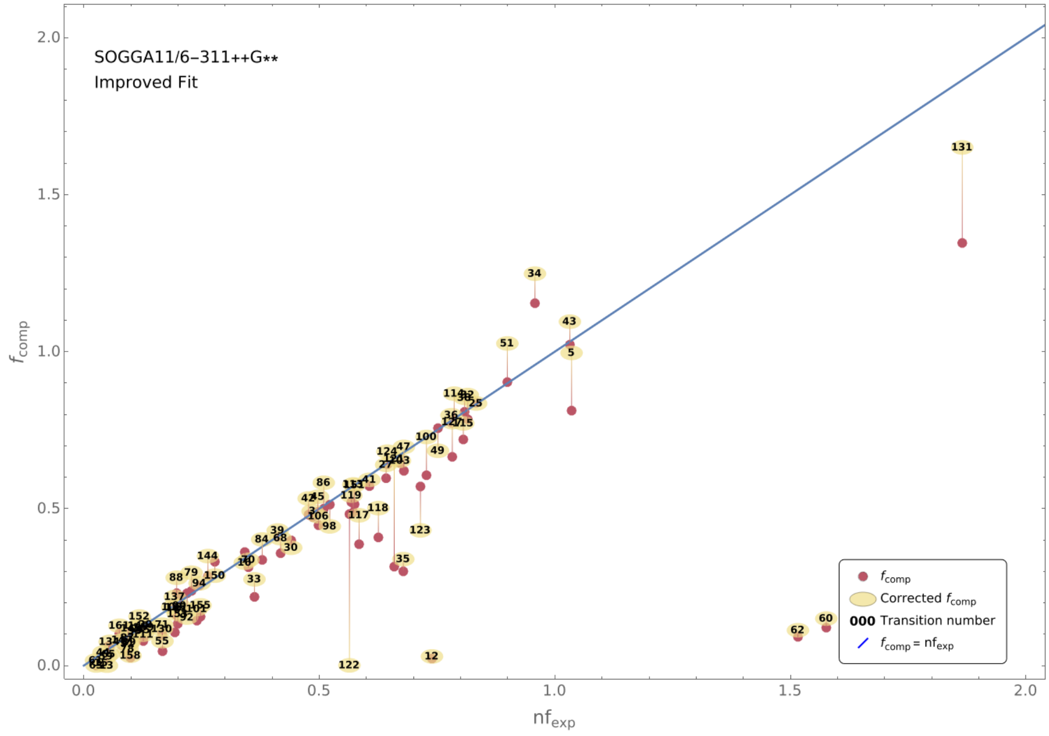

S3.2.6 SVWN

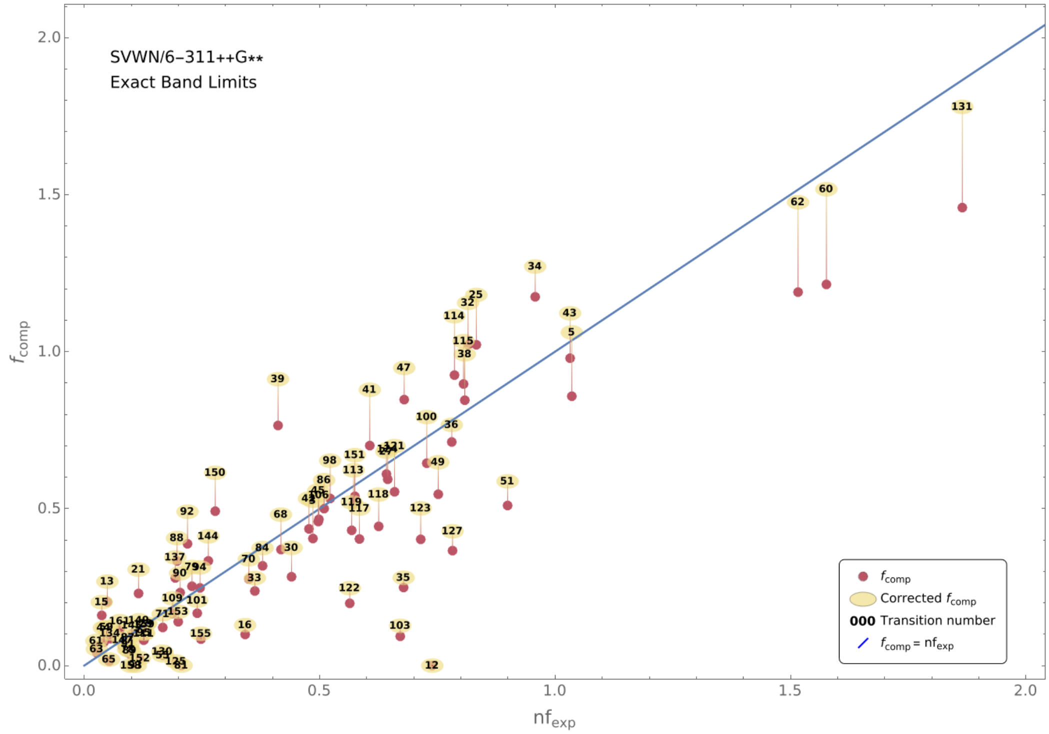

SVWN/6–311++G**
Exact Band Limits
f_{comp}
nf_{exp}
f_{comp}
Corrected f_{comp}
000 Transition number
$f_{comp} = nf_{exp}$

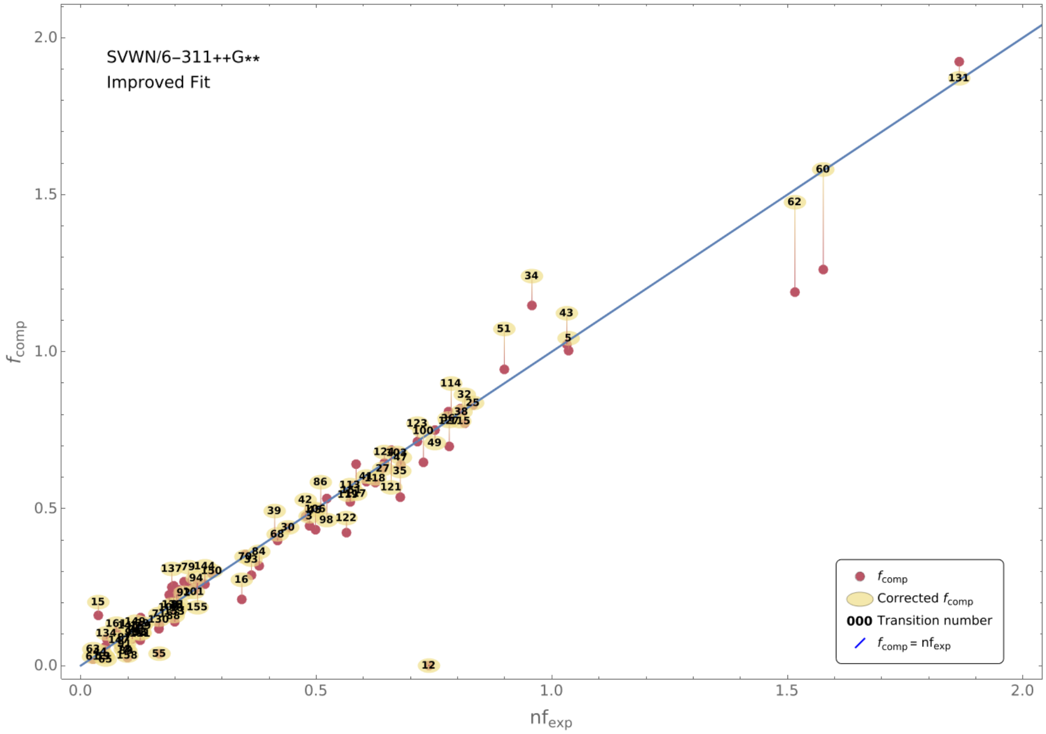

SVWN/6–311++G**
Improved Fit
f_{comp}
nf_{exp}
f_{comp}
Corrected f_{comp}
000 Transition number
$f_{comp} = nf_{exp}$

S3.2.7 SVWN5

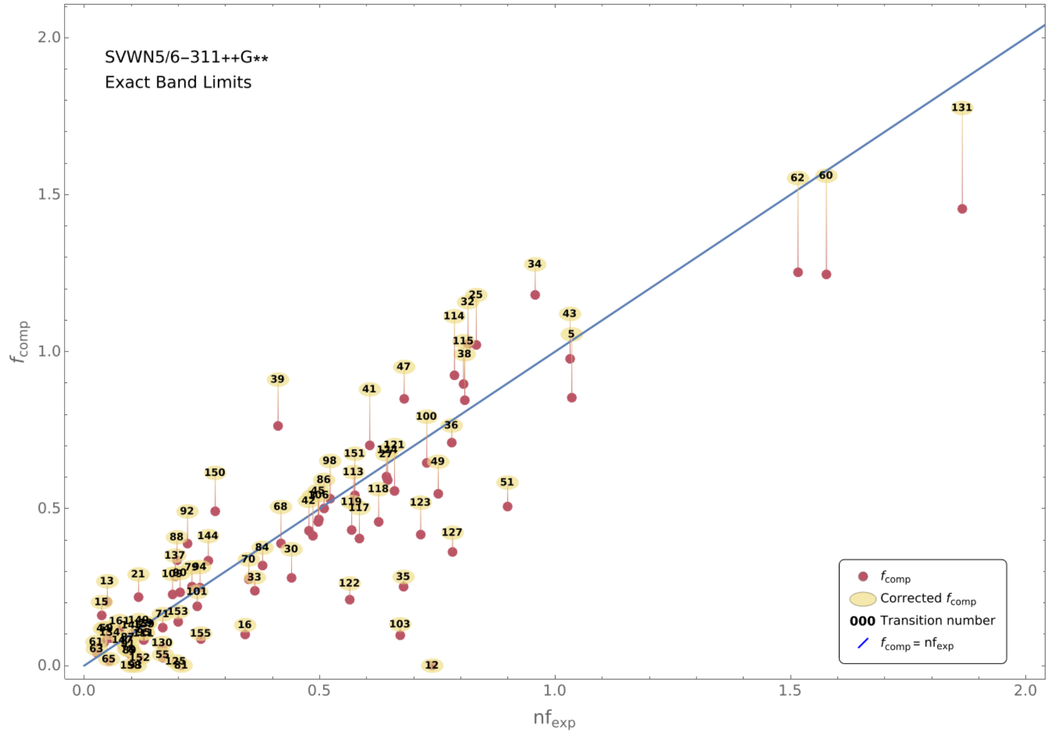

SVWN5/6–311++G**
Exact Band Limits
f_{comp}
nf_{exp}
f_{comp}
Corrected f_{comp}
000 Transition number
$f_{comp} = nf_{exp}$

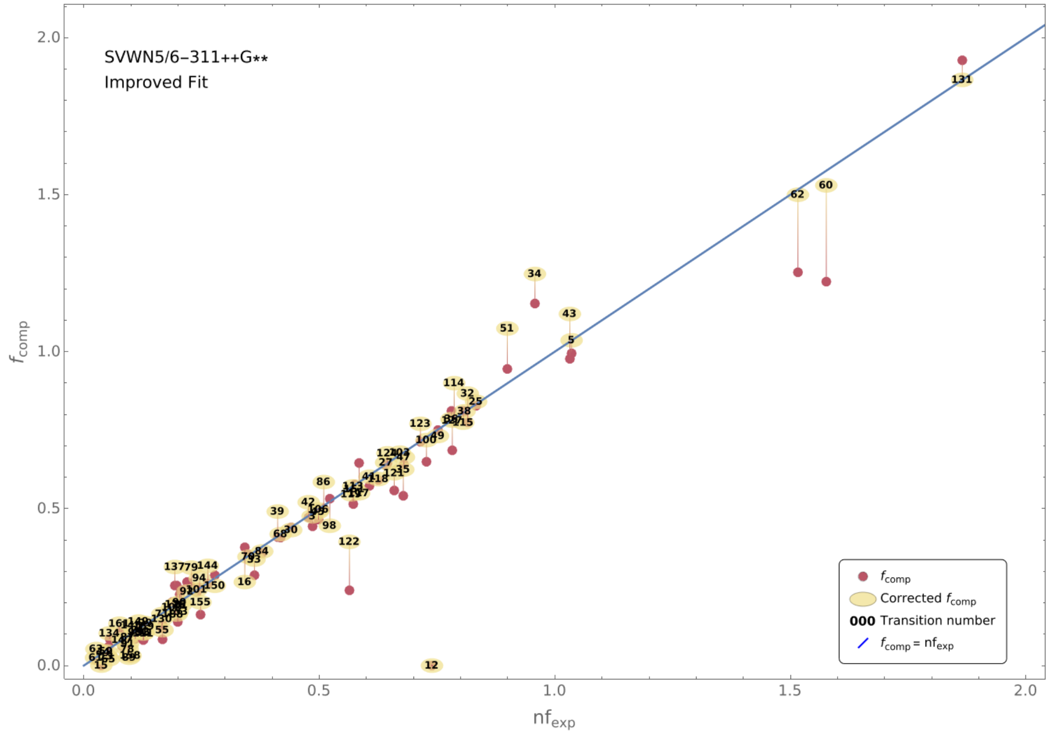

SVWN5/6–311++G**
Improved Fit
f_{comp}
nf_{exp}
f_{comp}
Corrected f_{comp}
000 Transition number
$f_{comp} = nf_{exp}$

S3.2.8 TPSS

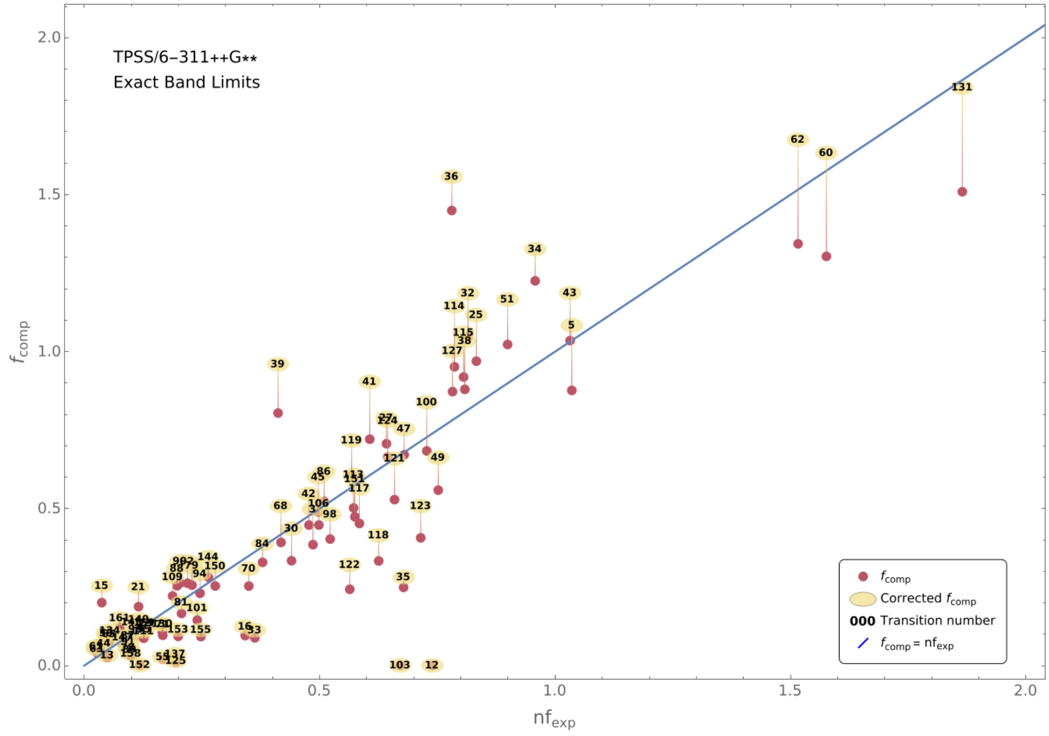
TPSS/6-311++G**
Exact Band Limits
f_{comp}
nf_{exp}
f_{comp}
Corrected f_{comp}
000 Transition number
$f_{comp} = nf_{exp}$

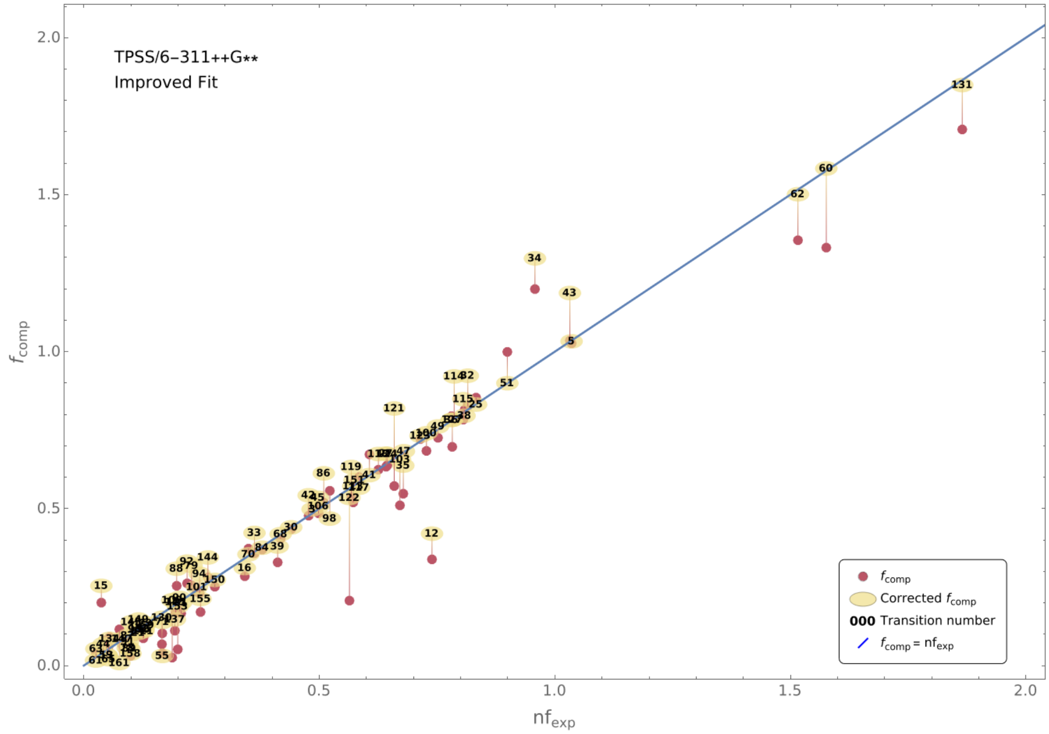
TPSS/6-311++G**
Improved Fit
f_{comp}
nf_{exp}
f_{comp}
Corrected f_{comp}
000 Transition number
$f_{comp} = nf_{exp}$

S3.3 TD-DFT/6-311++G**, hybrid functionals

S3.3.1 B3LYP

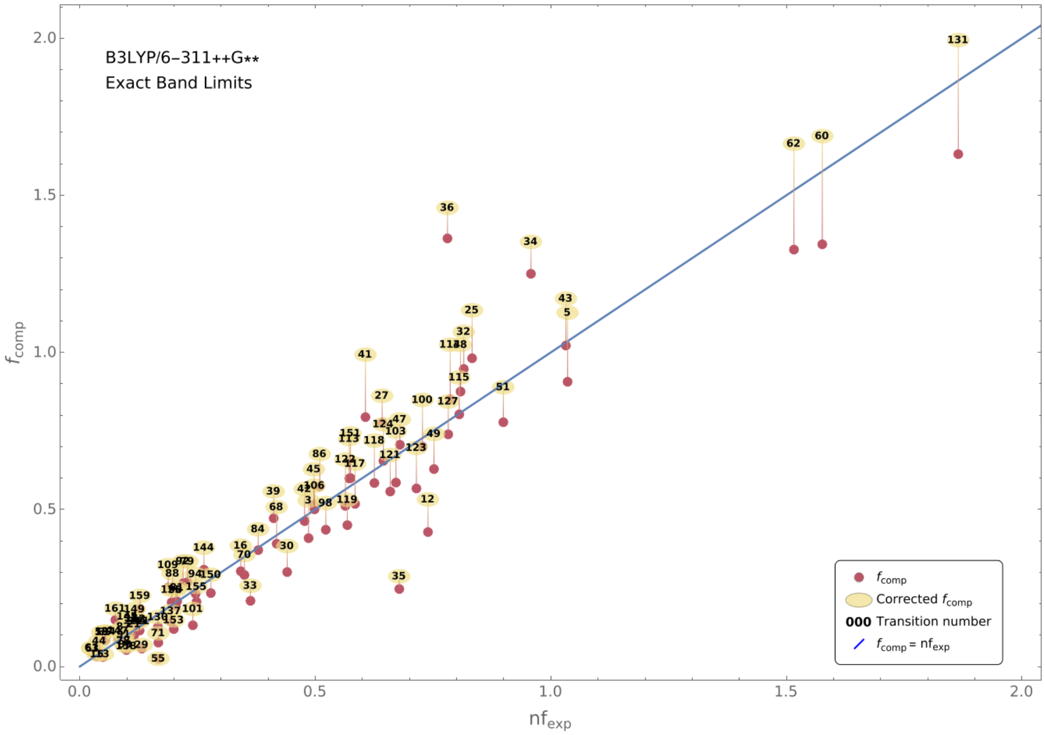

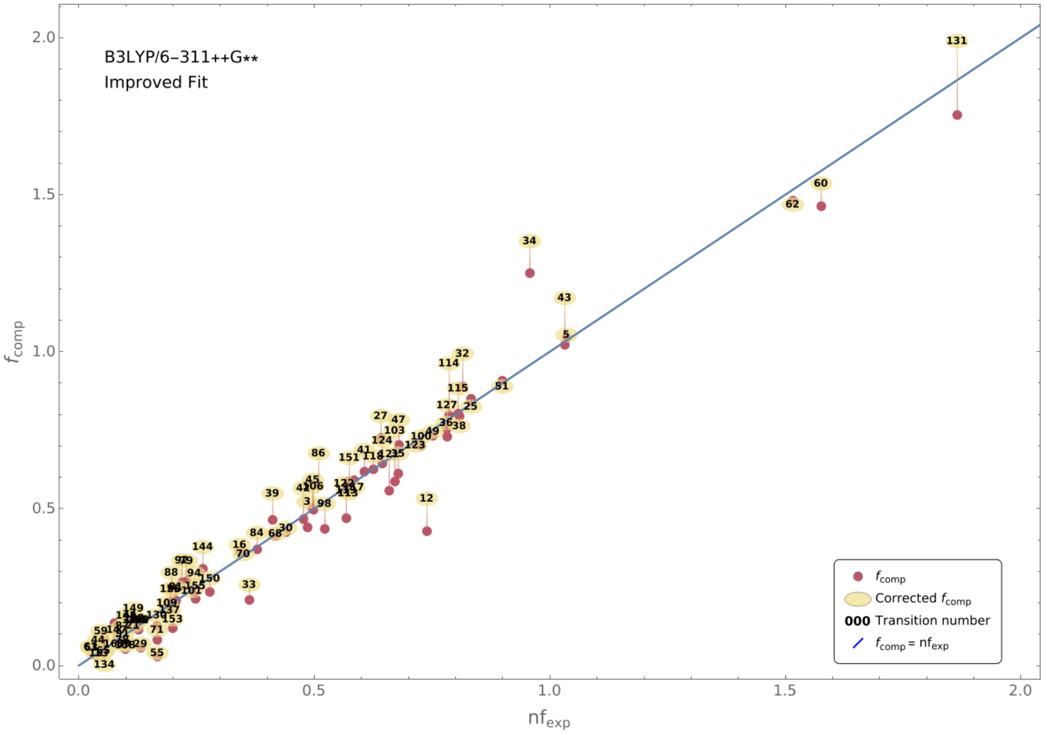

S3.3.2 B3P86

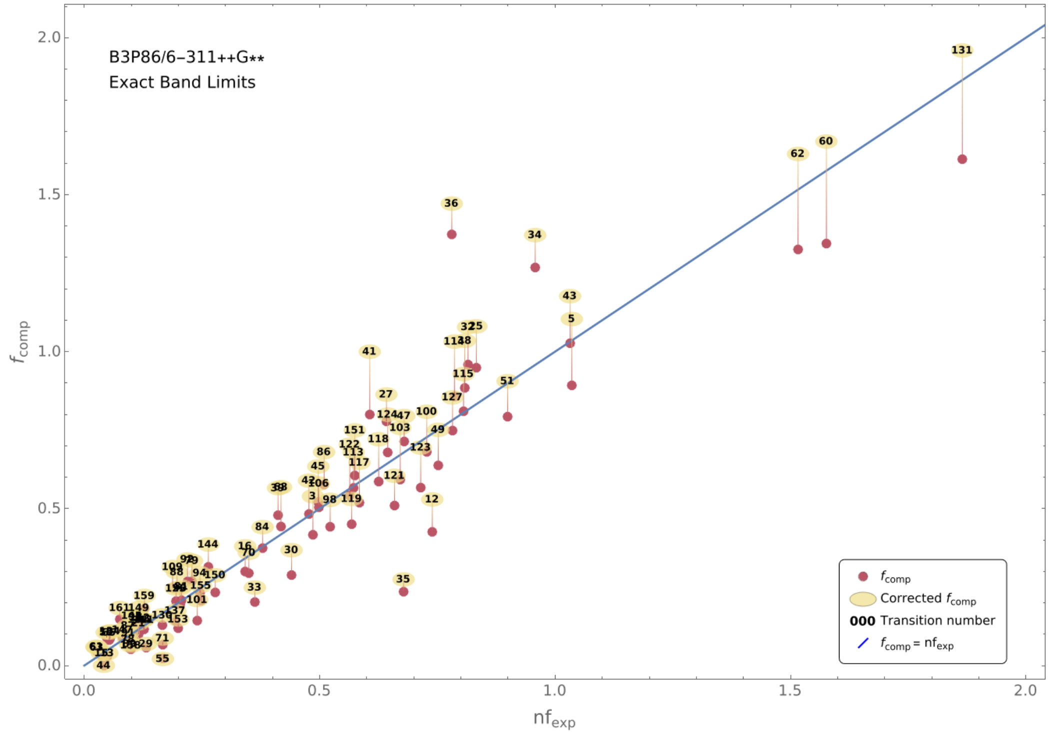
B3P86/6–311++G**
Exact Band Limits
f_{comp}
nf_{exp}
f_{comp}
Corrected f_{comp}
000 Transition number
$f_{comp} = nf_{exp}$

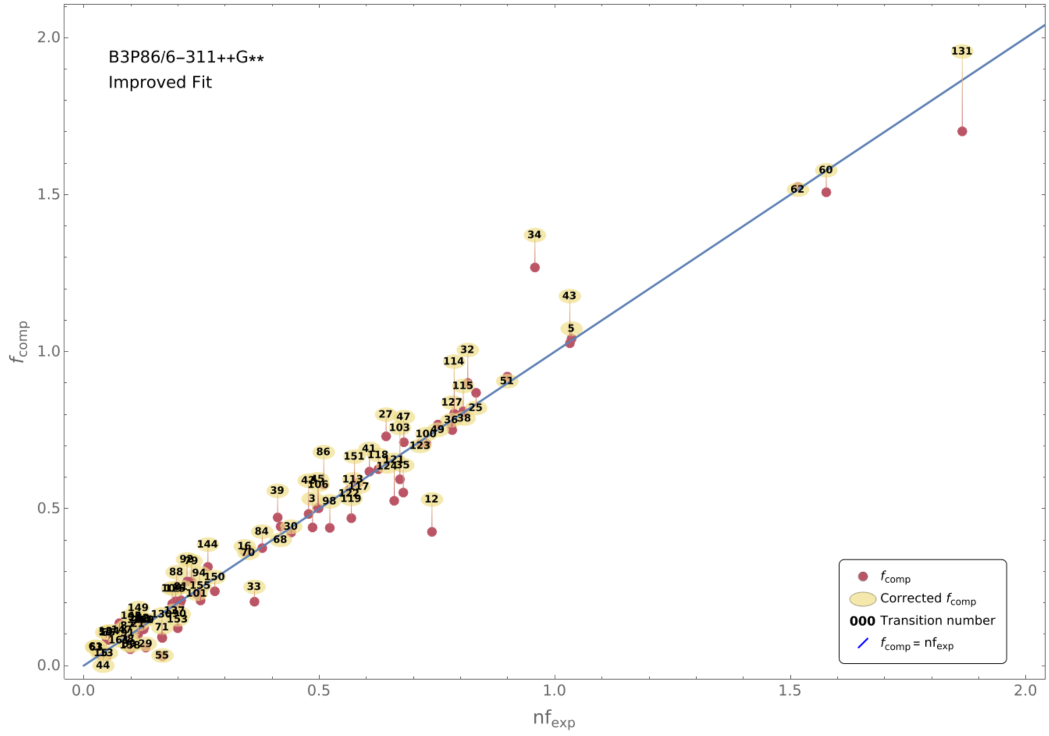
B3P86/6–311++G**
Improved Fit
f_{comp}
nf_{exp}
f_{comp}
Corrected f_{comp}
000 Transition number
$f_{comp} = nf_{exp}$

S3.3.3 BHandHLYP

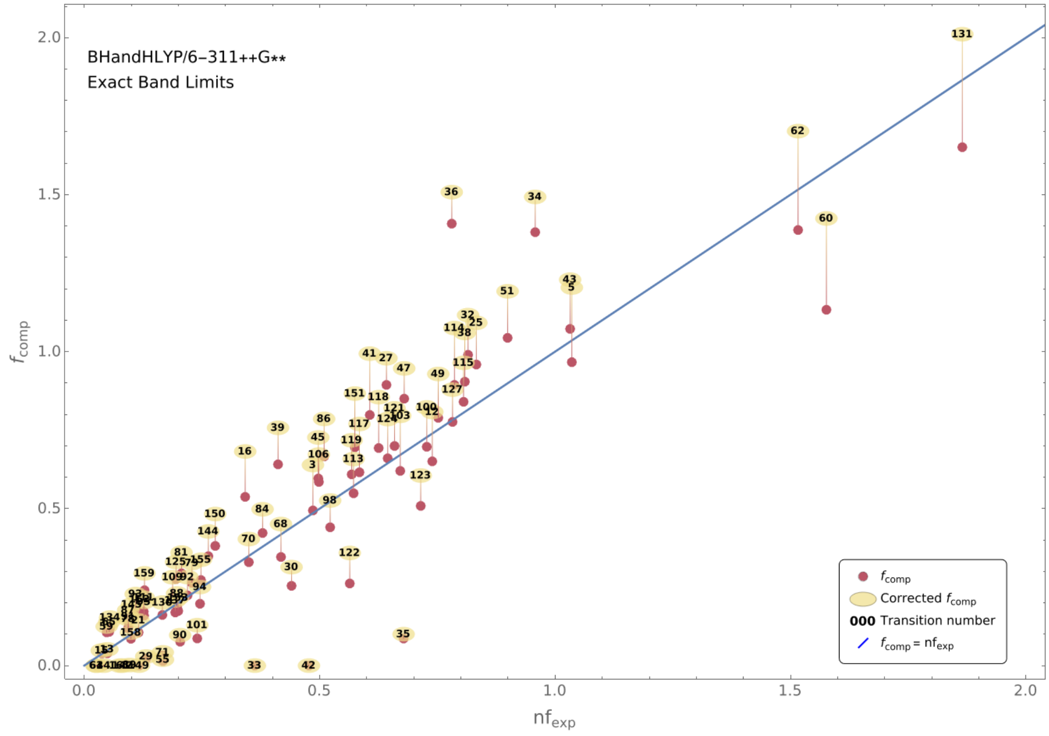

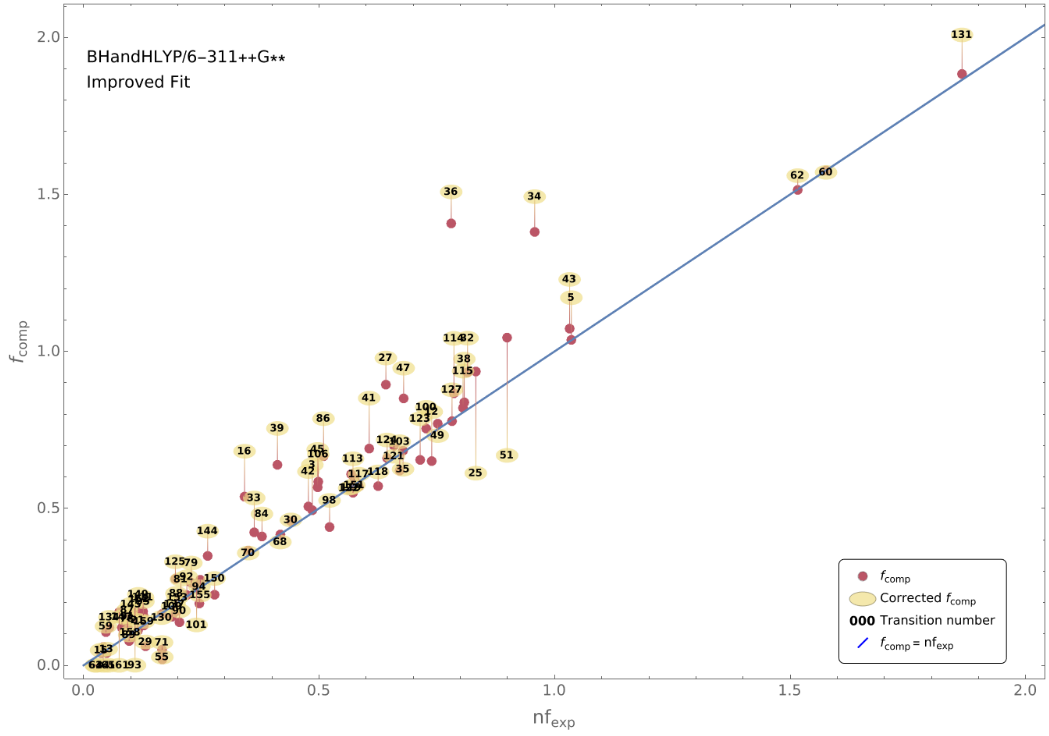

S3.3.4 M05

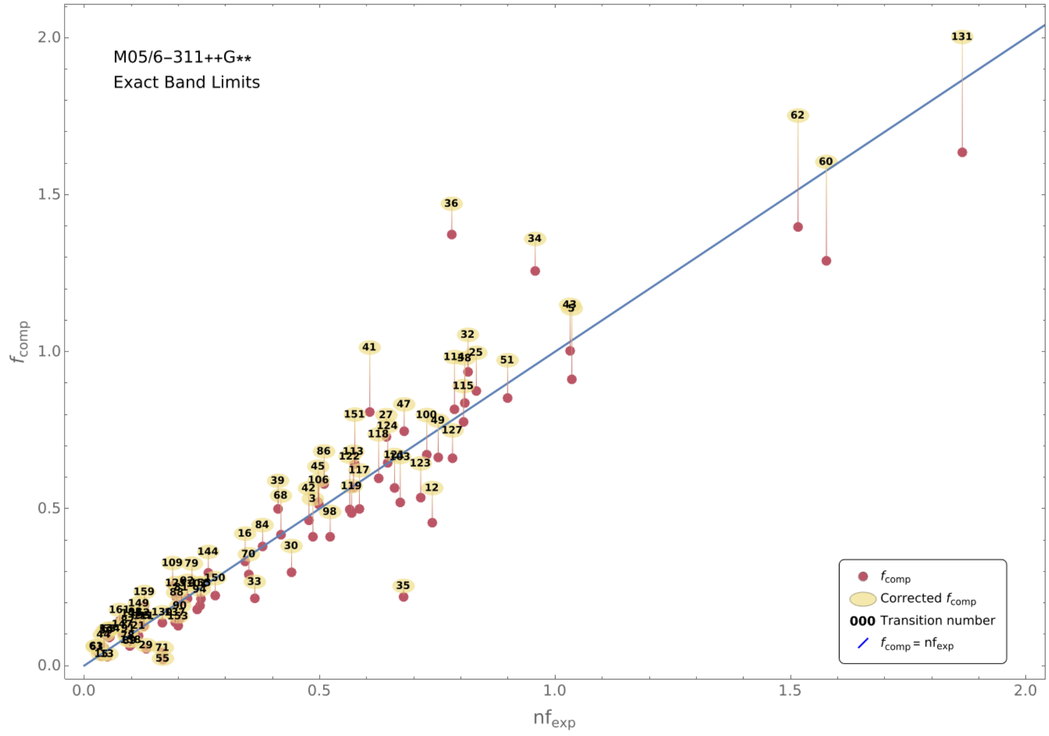

M05/6–311++G**
Exact Band Limits
f_{comp}
nf_{exp}
f_{comp}
Corrected f_{comp}
000 Transition number
$f_{comp} = nf_{exp}$

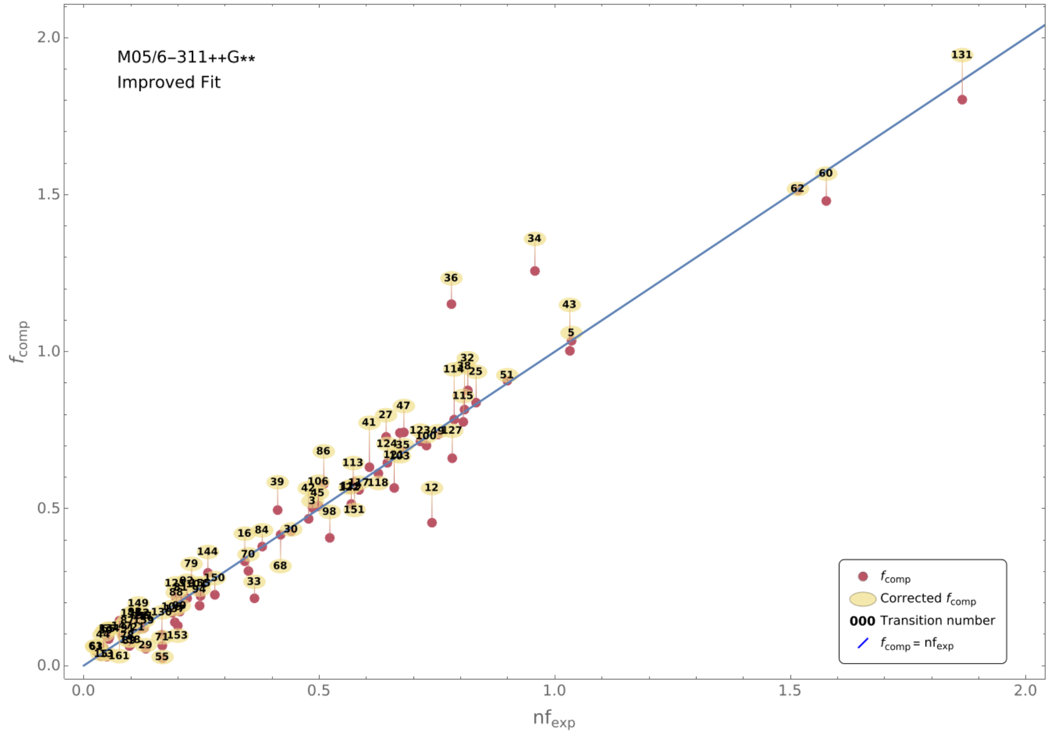

M05/6–311++G**
Improved Fit
f_{comp}
nf_{exp}
f_{comp}
Corrected f_{comp}
000 Transition number
$f_{comp} = nf_{exp}$

S3.3.5 mPW1PW91

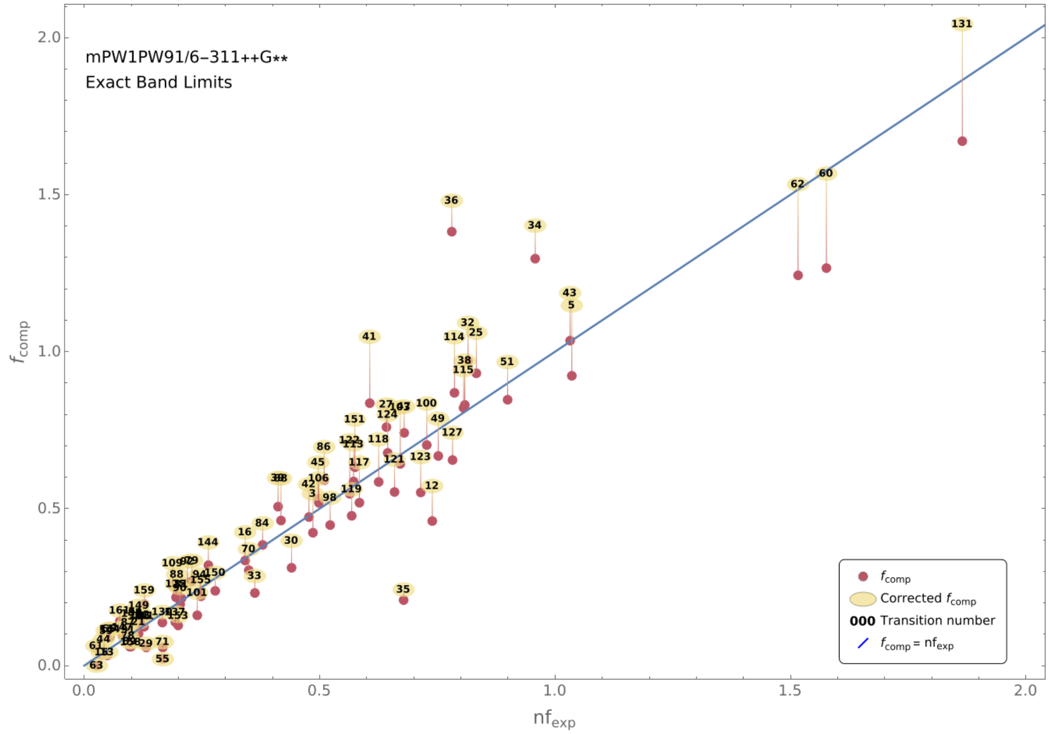

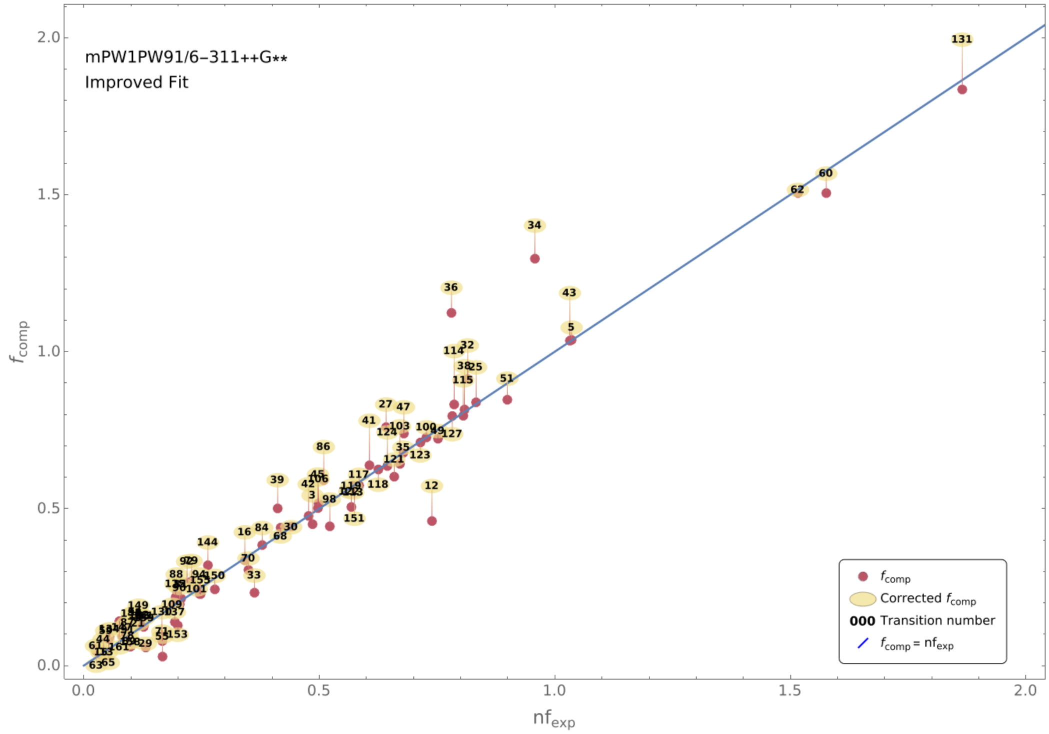

S3.3.6 O3LYP

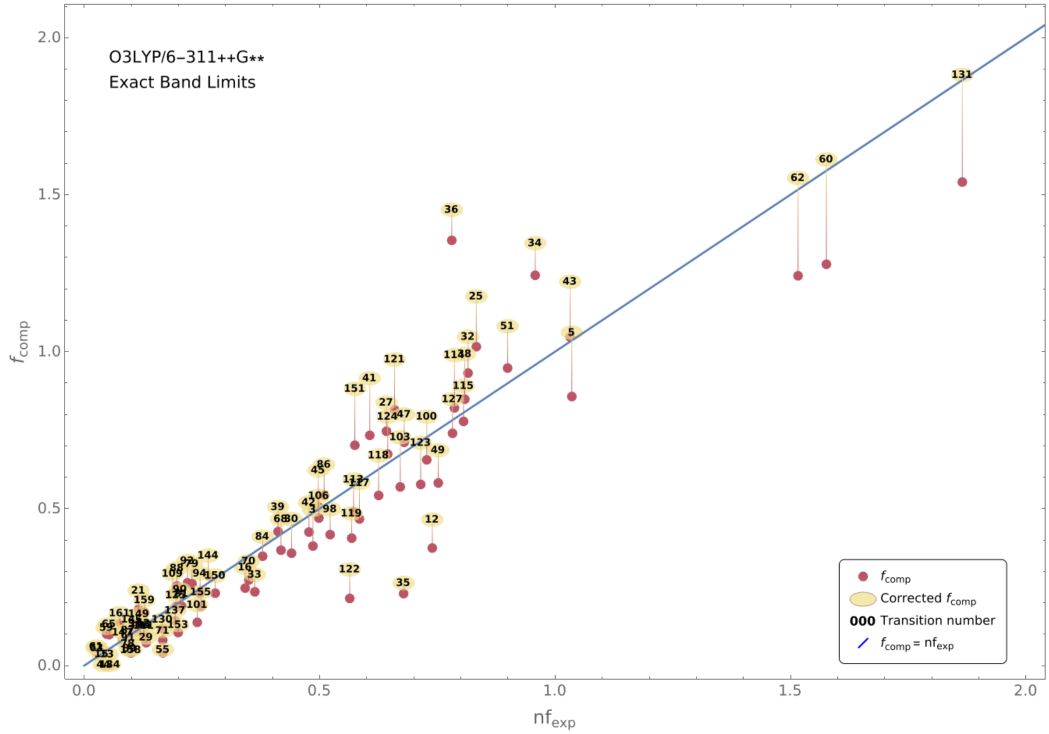
O3LYP/6–311++G**
Exact Band Limits
f_comp
Corrected f_comp
000 Transition number
f_comp = nf_exp
nf_exp
f_comp

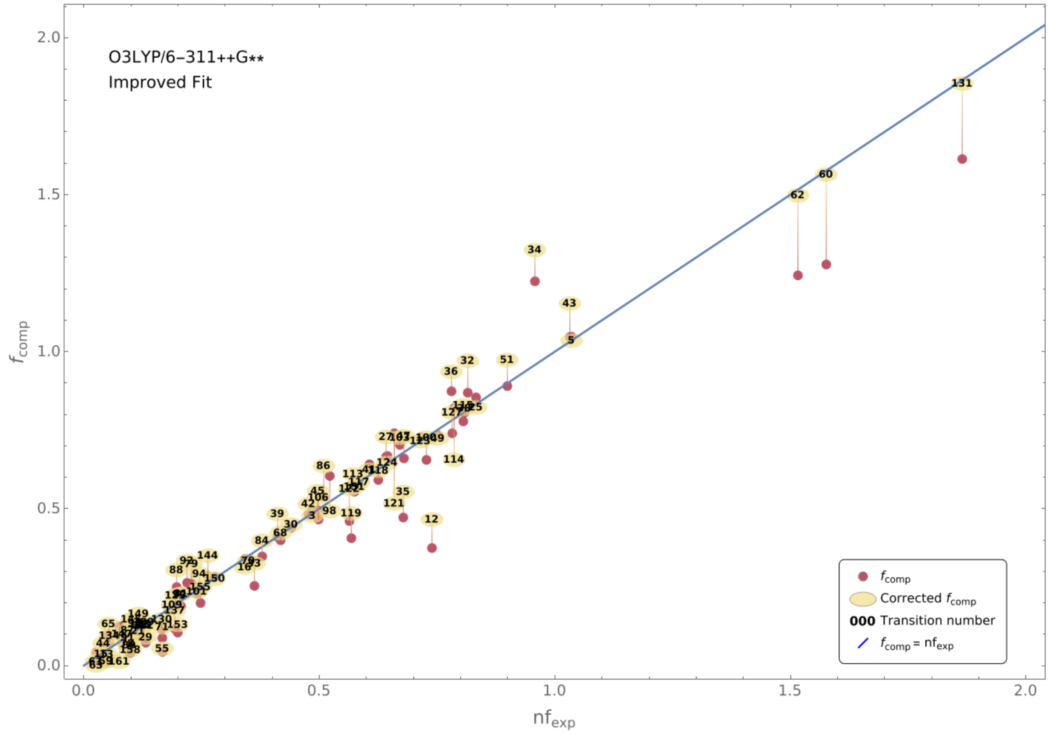
O3LYP/6–311++G**
Improved Fit
f_comp
Corrected f_comp
000 Transition number
f_comp = nf_exp
nf_exp
f_comp

S3.3.7 PBE0

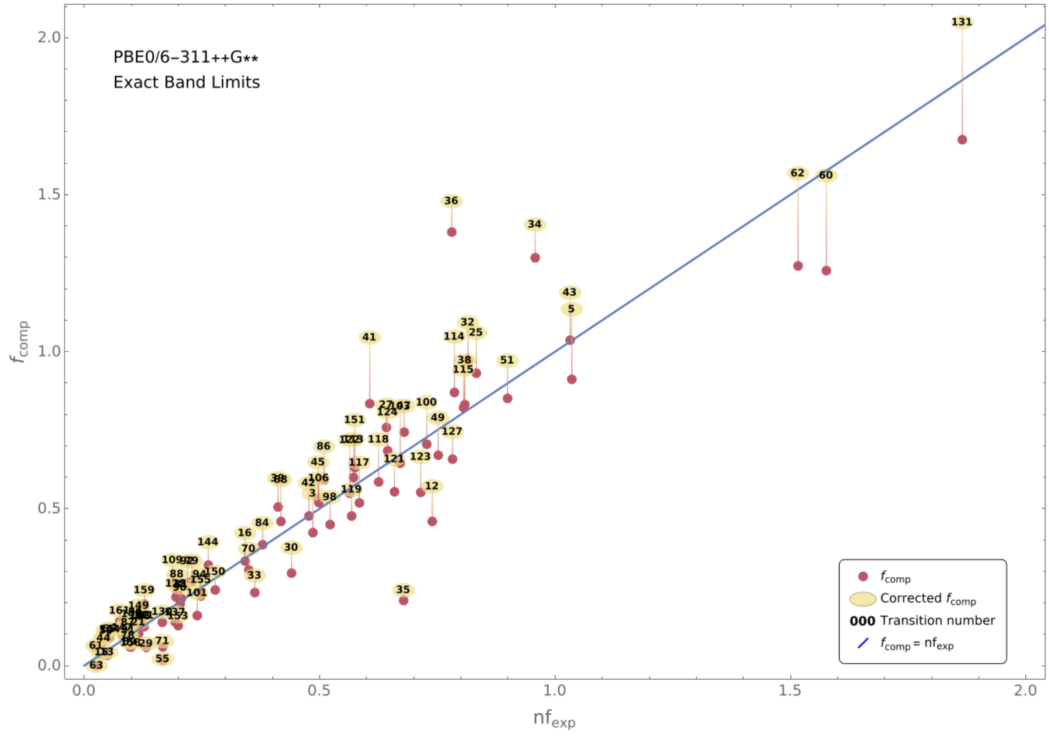
PBE0/6-311++G**
Exact Band Limits
f_{comp}
nf_{exp}
f_{comp}
Corrected f_{comp}
000 Transition number
$f_{comp} = nf_{exp}$

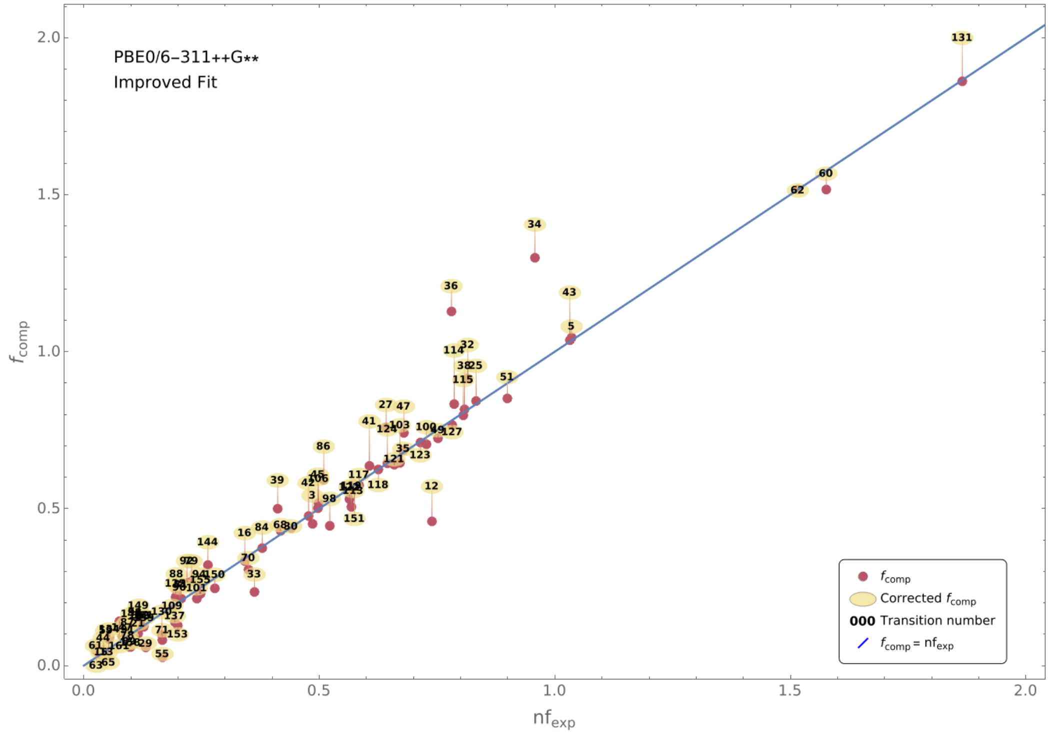
PBE0/6-311++G**
Improved Fit
f_{comp}
nf_{exp}
f_{comp}
Corrected f_{comp}
000 Transition number
$f_{comp} = nf_{exp}$

S3.3.8 SOGGA11-X

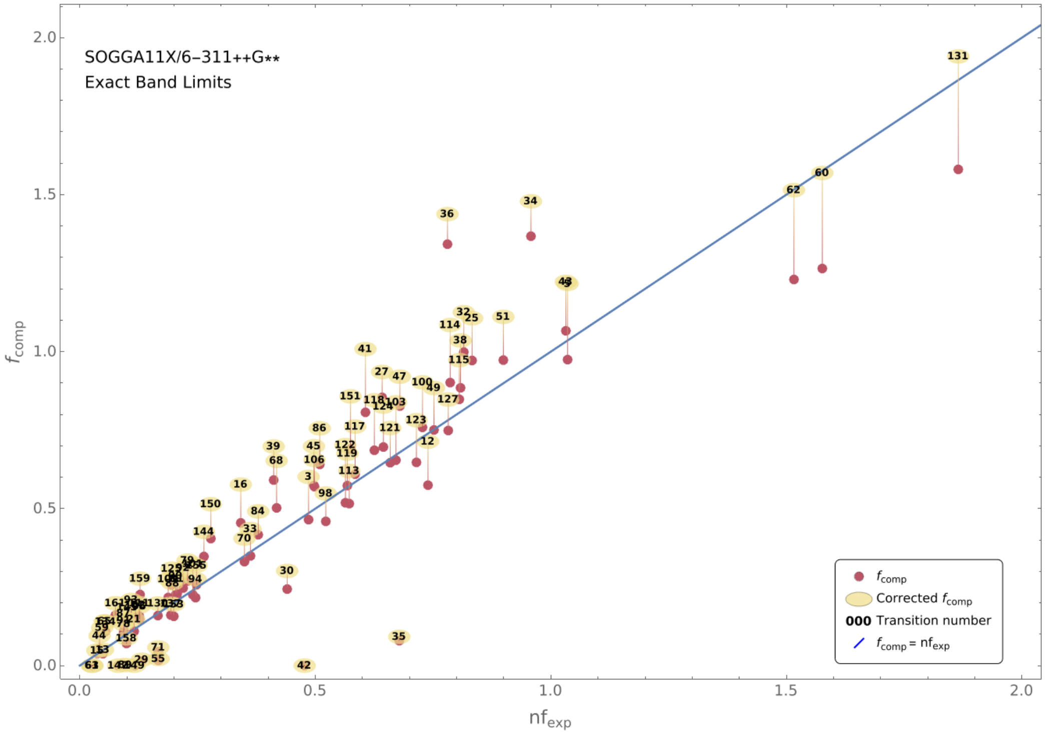

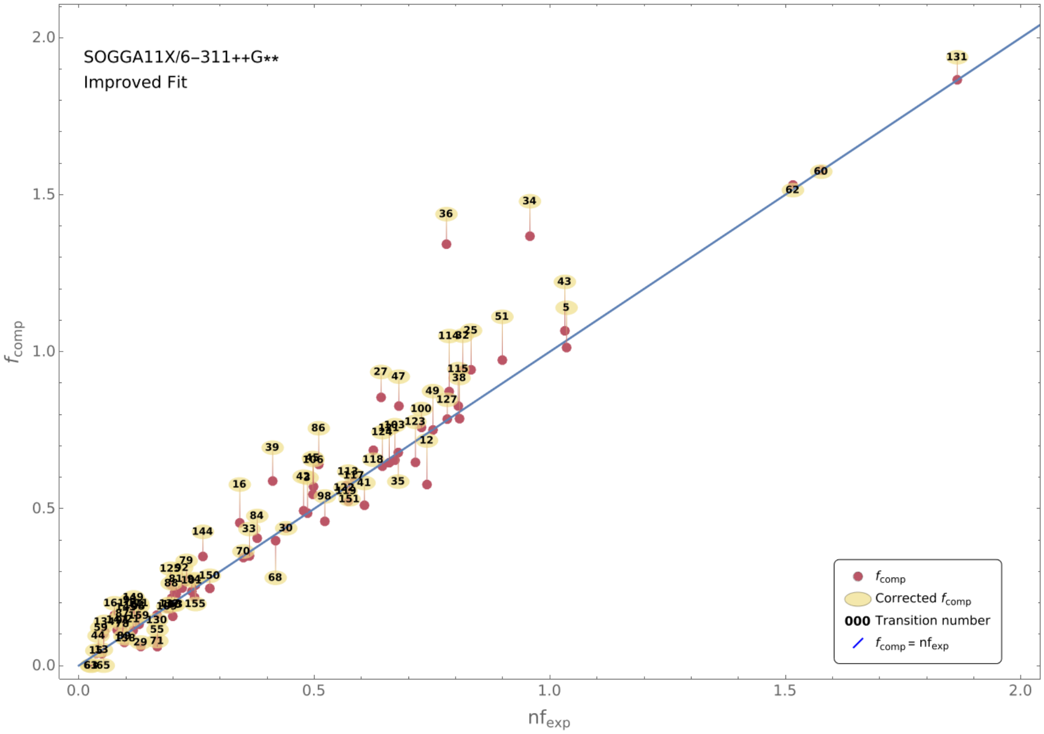

S3.4 TD-DFT/6-311++G**, long-range corrected hybrid functionals

S3.4.1 CAM-B3LYP

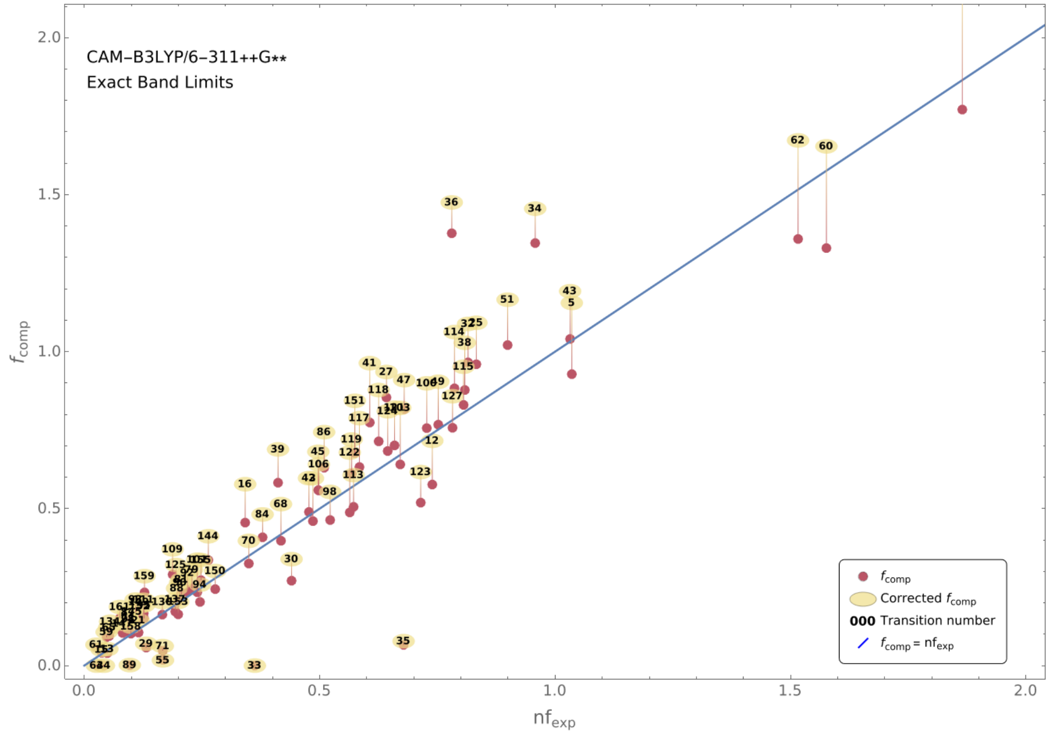

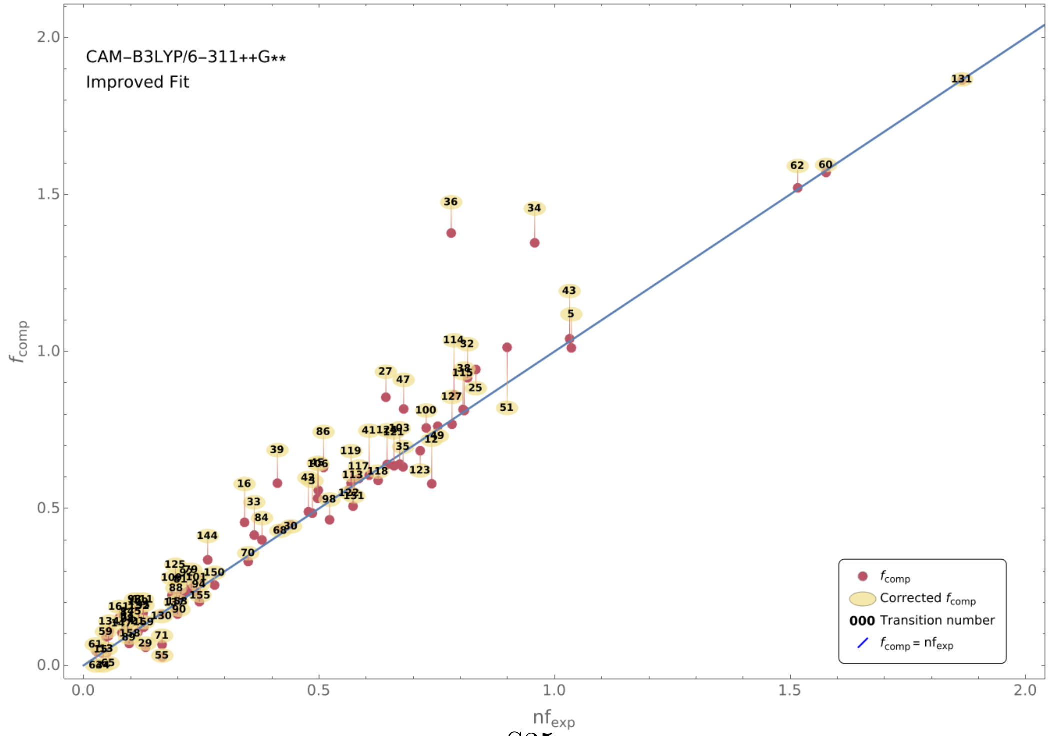

S3.4.2 LC-ωHPBE

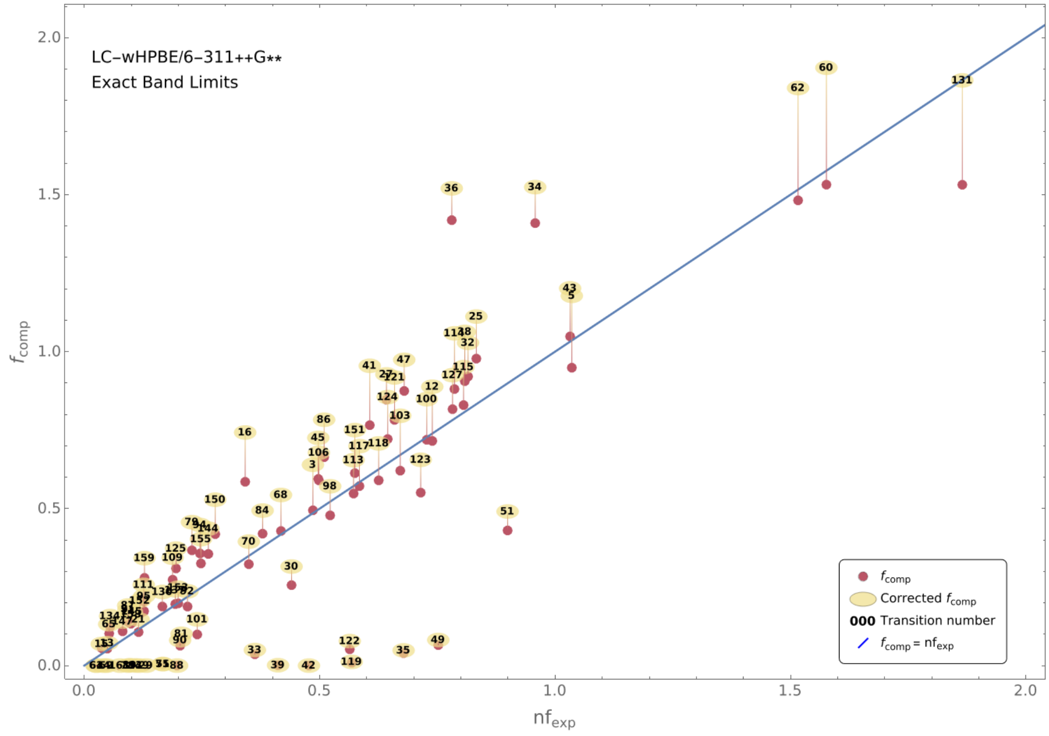

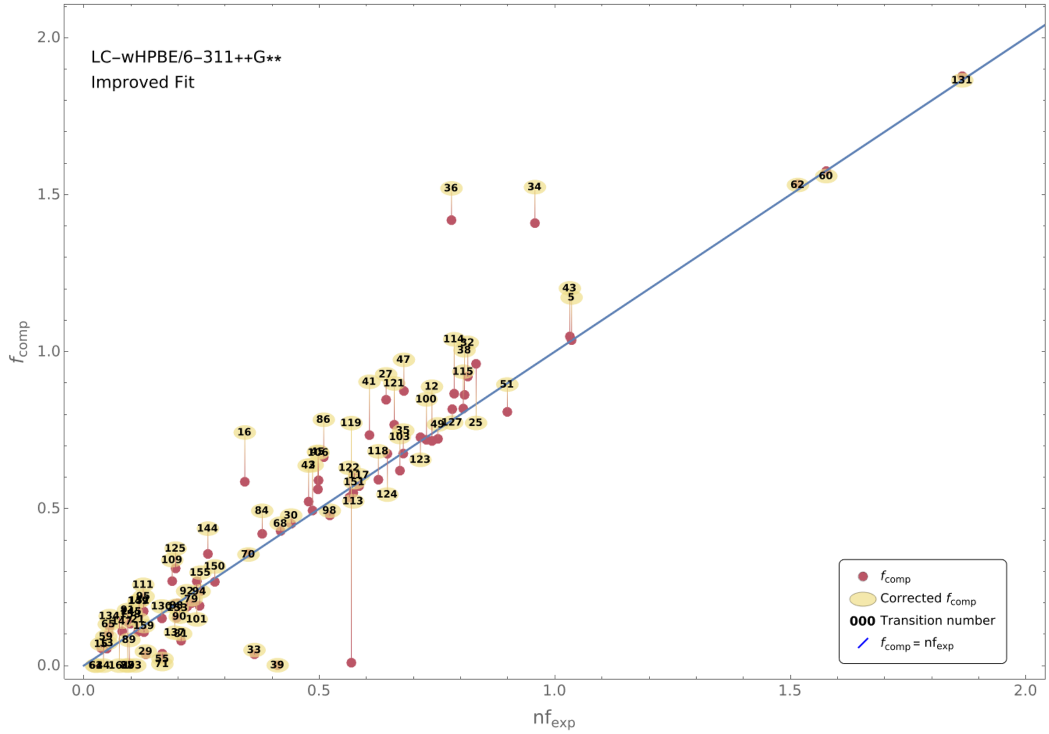

S3.4.3 ωB97X-D

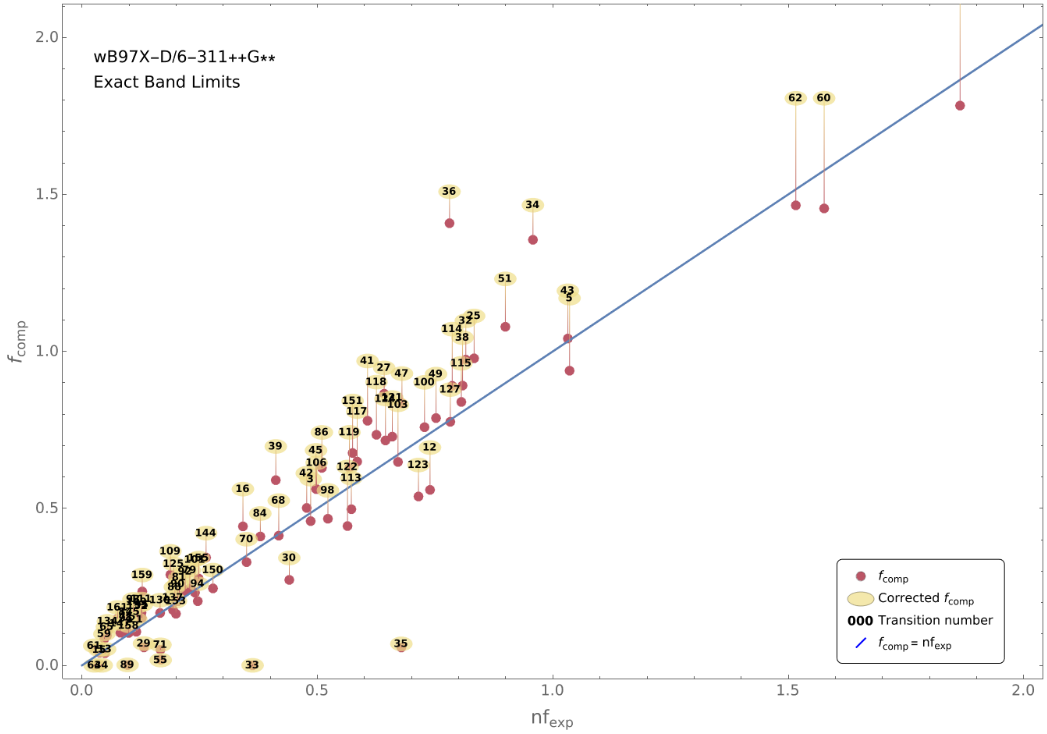
wB97X–D/6–311++G**
Exact Band Limits
f_{comp}
Corrected f_{comp}
000 Transition number
$f_{comp} = nf_{exp}$
nf_{exp}
f_{comp}

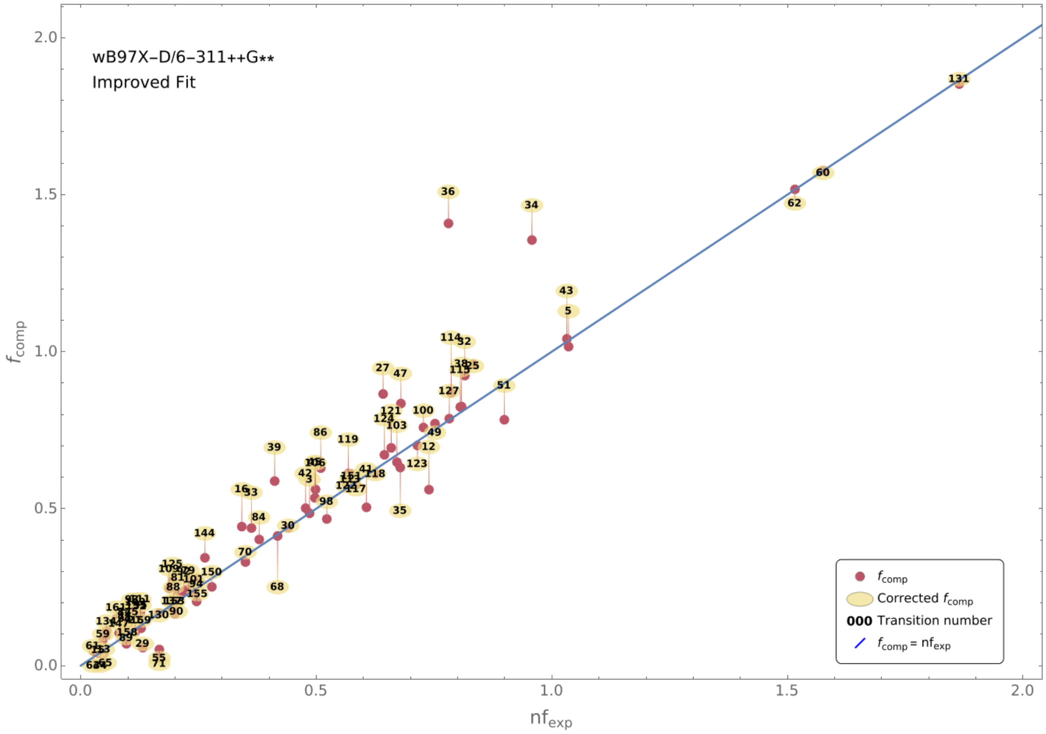
wB97X–D/6–311++G**
Improved Fit
f_{comp}
Corrected f_{comp}
000 Transition number
$f_{comp} = nf_{exp}$
nf_{exp}
f_{comp}

S4 Stats: $f_{\mathbf{comp}}$ and $f^{\mathbf{S}}_{\mathbf{comp}}$ compared to $n(\tilde{\nu})f_{\mathbf{exp}}$

S4.1 35 VHHM for wavefunction methods

S4.1.1 35 VHHM for wavefunction methods: $f_{\mathbf{comp}}$ vs. $n(\tilde{\nu})f_{\mathbf{exp}}$

Avg $nf_{\text{exp}} = 0.298506$

Table S2: Table: f_{comp} vs nf_{exp} /x-gauge/Exact Band Limits/

	CIS	HF	EOM-CCSD	LR-CCSD	TDA-B3LYP	B3LYP	GAS
MAE	0.20213	0.154428	0.107214	0.110339	0.0875343	0.035152	0.0722
Slope	0.475656	0.854469	0.524738	0.498576	1.25311	0.915508	0.771472
Intercept	-0.00674633	-0.0721272	0.0557168	0.0528919	-0.0045502	0.017598	-0.00232361
R^2	0.372771	0.73989	0.58322	0.573433	0.96476	0.979147	0.978438
$\|\Delta E\|$	2.29697	2.04943	0.987765	0.987842	0.366594	0.194237	0.215442
ΔE	-1.96982	-1.77584	-0.369483	-0.36936	-0.0226967	-0.0383761	0.102054
$E_{\text{comp}}/E_{\text{exp}}$	0.490587	0.541302	0.930338	0.930367	0.998425	0.989187	1.02387

Table S3: Table: f_{comp} vs nf_{exp} /p-gauge/Exact Band Limits/

	CIS	HF	EOM-CCSD	LR-CCSD	TDA-B3LYP	B3LYP
MAE	0.256201	0.157454	0.178395	0.157698	0.245841	0.0354469
Slope	0.179256	0.806478	0.340916	0.380948	0.234669	0.884281
Intercept	-0.0112033	-0.0651644	0.018345:q9	0.0270932	-0.0173843	0.018065
R^2	0.320922	0.735417	0.565044	0.569188	0.797069	0.982221
$\|\Delta E\|$	2.29919	2.04936	0.991367	0.990235	0.357805	0.193666
ΔE	-1.96759	-1.77598	-0.366188	-0.367438	-0.0293323	-0.0396048
$E_{\text{comp}}/E_{\text{exp}}$	0.491051	0.541269	0.931054	0.930782	0.997096	0.988917

Table S4: Table: f_{comp} vs nf_{exp} /xp-gauge/Exact Band Limits/

	CIS	HF	EOM-CCSD	LR-CCSD	TDA-B3LYP	B3LYP
MAE	0.226915	0.155899	0.140275	0.129864	0.177221	0.0352389
Slope	0.287601	0.830117	0.423194	0.437415	0.523942	0.899681
Intercept	-0.0142593	-0.068661	0.0319053	0.0386747	-0.0351143	0.0177396
R^2	0.360711	0.737892	0.580842	0.573456	0.90672	0.980853
$\|\Delta E\|$	2.29856	2.04941	0.989803	0.989014	0.361076	0.193965
ΔE	-1.96823	-1.77592	-0.367502	-0.368387	-0.0231371	-0.0390178
$E_{\text{comp}}/E_{\text{exp}}$	0.490931	0.541283	0.93077	0.930576	0.998372	0.989044

Improving fit:

Table S5: Table: f_{comp} vs nf_{exp} /x-gauge/Improved Fit/

	CIS	HF	EOM-CCSD	LR	TDA-B3LYP	B3LYP	GAS
MAE	0.129013	0.109366	0.0362503	0.0398846	0.0613286	0.0272994	0.0444949
Slope	1.17413	1.07467	0.988114	0.929664	1.06443	0.957696	0.805275
Intercept	-0.0767864	-0.0726422	-0.00400958	-0.00148763	0.0370552	0.00988454	0.0161003
R^2	0.848087	0.8633	0.981245	0.979275	0.949683	0.988671	0.963146
$\|\Delta E\|$	1.90394	1.74245	0.479557	0.478801	0.287564	0.176893	0.368558
ΔE	-1.05934	-1.1559	0.47306	0.47235	0.185994	-0.0174046	0.129636
$E_{\text{comp}}/E_{\text{exp}}$	0.725429	0.692744	1.10982	1.10972	1.04204	0.993299	1.02064

Table S6: Table: f_{comp} vs nf_{exp} /p-gauge/Improved Fit/

	CIS	HF	EOM-CCSD	LR-CCSD	TDA-B3LYP	B3LYP
MAE	0.0956406	0.119363	0.0893423	0.0734251	0.123598	0.0292554
Slope	0.524879	1.0227	0.629039	0.720725	0.313693	0.940066
Intercept	0.0708233	-0.0786023	0.0315107	0.0149362	0.0827093	0.013913
R^2	0.662673	0.828307	0.863544	0.915289	0.510134	0.9856
$\|\Delta E\|$	1.67797	1.73986	0.82887	0.66787	1.35296	0.18724
ΔE	1.67797	-1.16365	0.822066	0.660948	1.35176	-0.00882724
$E_{\text{comp}}/E_{\text{exp}}$	1.39869	0.691794	1.18969	1.15356	1.31962	0.995247

Table S7: Table: f_{comp} vs nf_{exp} /xp-gauge/Improved Fit/

	CIS	HF	EOM-CCSD	LR-CCSD	TDA-B3LYP	B3LYP
MAE	0.09862	0.10635	0.0610114	0.0541206	0.0901097	0.0270011
Slope	0.84272	1.05456	0.798407	0.85094	0.630628	0.938197
Intercept	-0.0406201	-0.0688048	0.00310759	-0.00288528	0.0285736	0.0106024
R^2	0.86647	0.863881	0.953888	0.958752	0.800771	0.989785
$\|\Delta E\|$	1.25044	1.7447	0.54626	0.53152	0.851003	0.176536
ΔE	0.829897	-1.15365	0.539706	0.52487	0.81993	-0.0180902
$E_{\text{comp}}/E_{\text{exp}}$	1.18972	0.693293	1.12583	1.12278	1.19621	0.993157

S4.1.2 35 VHHM for wavefunction methods: $f^{\mathbf{S}}_{\mathbf{comp}}$ vs. $n(\tilde{\nu})f_{\mathbf{exp}}$

Avg $nf_{\text{exp}} = 0.298506$

Table S8: Table: $f^{\text{S}}_{\text{comp}}$ vs nf_{exp} /x-gauge/Exact Band Limits/

	CIS	HF	EOM-CCSD	LR	TDA-B3LYP	B3LYP	GAS
MAE	0.218787	0.160635	0.107022	0.107288	0.16057	0.0619571	0.039464
Slope	0.565371	1.02076	0.59319	0.535279	1.50528	1.10073	0.927431
Intercept	-0.00462095	-0.0833168	0.0607433	0.0547959	-0.000478805	0.0255625	0.000715939
R^2	0.368241	0.735878	0.610525	0.590986	0.966738	0.981328	0.979795
$\|\Delta E\|$	2.29683	2.04891	0.987836	0.987957	0.366436	0.193936	0.216088
ΔE	-1.96996	-1.77619	-0.369123	-0.368852	-0.0229467	-0.0382819	0.102808
$E_{\text{comp}}/E_{\text{exp}}$	0.490556	0.541232	0.930394	0.930458	0.998367	0.989197	1.02404

Table S9: Table: $f^{\text{S}}_{\text{comp}}$ vs nf_{exp} /p-gauge/Exact Band Limits/

	CIS	HF	EOM-CCSD	LR	TDA-B3LYP	B3LYP	GAS
MAE	0.247789	0.159812	0.162501	0.11715	0.233983	0.0532154	0.042172
Slope	0.213112	0.963302	0.389179	0.484959	0.286107	1.06351	0.902416
Intercept	-0.0128982	-0.0748859	0.0198335	0.0434654	-0.0208819	0.0262007	0.00111177
R^2	0.325905	0.730229	0.584412	0.582049	0.808826	0.983856	0.980563
$\|\Delta E\|$	2.29933	2.0489	0.991249	0.989103	0.357304	0.193378	0.216075
ΔE	-1.96746	-1.77638	-0.366389	-0.368796	-0.0280885	-0.0395922	0.100887
$E_{\text{comp}}/E_{\text{exp}}$	0.491078	0.541188	0.930987	0.930461	0.997427	0.988905	1.02361

Table S10: Table: $f^{\text{S}}_{\text{comp}}$ vs nf_{exp} /xp-gauge/Exact Band Limits/

	CIS	HF	EOM-CCSD	LR	TDA-B3LYP	B3LYP	GAS
MAE	0.214535	0.159541	0.114345	0.109194	0.151526	0.0570069	0.0408154
Slope	0.341596	0.991611	0.505385	0.521933	0.632094	1.08202	0.914652
Intercept	-0.0158714	-0.0791193	0.0399423	0.0484541	-0.0417039	0.0257144	0.000756214
R^2	0.362979	0.733336	0.59321	0.58512	0.910655	0.982789	0.980519
$\|\Delta E\|$	2.2986	2.04888	0.989644	0.988839	0.360872	0.193719	0.21585
ΔE	-1.96819	-1.77626	-0.367413	-0.36832	-0.0229591	-0.0389401	0.101788
$E_{\text{comp}}/E_{\text{exp}}$	0.490938	0.541215	0.930792	0.930593	0.998433	0.989051	1.02381

Improving fit:

Table S11: Table: $f^{\mathrm{S}}_{\mathrm{comp}}$ vs nf_{exp} /x-gauge/Improved Fit/

	CIS	HF	EOM-CCSD	LR	TDA-B3LYP	B3LYP	GAS
MAE	0.157521	0.122651	0.0357691	0.0342526	0.122541	0.0517971	0.0259051
Slope	1.06192	1.08381	1.02988	0.989421	1.24977	1.07551	0.973243
Intercept	-0.0874625	-0.0483263	0.00135251	-0.00610838	0.0290738	0.0211398	-0.0047134
R^2	0.761665	0.817226	0.975897	0.981333	0.925054	0.982982	0.987818
$\lvert\Delta E\rvert$	2.02856	1.73884	0.450412	0.454682	0.391958	0.204075	0.306058
ΔE	-1.36171	-1.188	0.444204	0.448625	0.0364942	-0.0159125	0.0432988
$E_{\mathrm{comp}}/E_{\mathrm{exp}}$	0.644711	0.690456	1.1032	1.10422	1.00838	0.994264	1.00112

Table S12: Table: $f^{\mathrm{S}}_{\mathrm{comp}}$ vs nf_{exp} /p-gauge/Improved Fit/

	CIS	HF	EOM-CCSD	LR	TDA-B3LYP	B3LYP	GAS
MAE	0.105519	0.114168	0.0803583	0.0476657	0.102774	0.0429366	0.0287189
Slope	0.632966	1.05136	0.734626	0.876371	0.421233	1.04268	0.969966
Intercept	0.0228129	-0.0444247	0.00141823	-1.36493e-05	0.0743278	0.0218859	-0.00349522
R^2	0.783028	0.828349	0.920376	0.97239	0.546063	0.987096	0.983118
$\lvert\Delta E\rvert$	1.42635	1.65572	0.639535	0.49714	1.23739	0.205846	0.292788
ΔE	1.42635	-1.03799	0.632648	0.489993	1.23739	-0.0154314	0.0216589
$E_{\mathrm{comp}}/E_{\mathrm{exp}}$	1.33674	0.726892	1.14942	1.11375	1.29739	0.994329	0.995982

Table S13: Table: $f^{\mathrm{S}}_{\mathrm{comp}}$ vs nf_{exp} /xp-gauge/Improved Fit/

	CIS	HF	EOM-CCSD	LR	TDA-B3LYP	B3LYP	GAS
MAE	0.0894903	0.117257	0.0420737	0.0352611	0.0742406	0.0472211	0.0271331
Slope	0.990991	1.07946	0.94879	0.951749	0.75706	1.05698	0.960221
Intercept	-0.0651255	-0.0466903	-0.0191141	-0.00491453	0.00963292	0.0210227	-0.00296072
R^2	0.873913	0.82645	0.981988	0.98327	0.895145	0.984355	0.986454
$\lvert\Delta E\rvert$	1.61131	1.7481	0.474208	0.484051	0.657531	0.204899	0.293061
ΔE	-0.106072	-1.17874	0.467902	0.477643	0.633988	-0.0162331	0.0163208
$E_{\mathrm{comp}}/E_{\mathrm{exp}}$	0.942816	0.692364	1.10889	1.11092	1.15043	0.994185	0.994718

S4.2 Scaling factors for EOM-CCSD and LR-CCSD across the full 35 VHHM set of transitions

Table S14: Scaling factors C and C^S to minimize $\mathrm{MAE}(Cnf_{\mathrm{exp}}, f_{\mathrm{comp}})$ and $\mathrm{MAE}\big(C^S nf_{\mathrm{exp}}, f^{\mathrm{S}}_{\mathrm{comp}}\big)$ for EOM-CCSD and LR-CCSD. Data is presented for the length, momentum, and mixed gauges. The analysis was carried for the set of 35 VHHM transitions.

**Method**	C^S	C
EOM-CCSD length gauge	1.11804	0.971153
EOM-CCSD momentum gauge	0.630623	0.559464
EOM-CCSD mixed gauge	0.893272	0.72947
LR-CCSD length gauge	0.977419	0.914703
LR-CCSD momentum gauge	0.846154	0.644166
LR-CCSD mixed gauge	0.936828	0.791216

S4.3 Pure functionals / 6-311++G**

S4.3.1 Pure functionals / 6-311++G**: $f_{\mathbf{comp}}$ vs $n(\tilde{\nu})f_{\mathbf{exp}}$

Avg $nf_{\text{exp}} = 0.392273$

Table S15: Table: f_{comp} vs $n(\tilde{\nu})f_{\text{exp}}$ PCM/TD-DFT/6-311++G**/x-gauge/Exact Band Limits/

	BLYP	N12	OLYP	PBE	SOGGA11	SVWN	SVWN5	TPSS
MAE	0.109394	0.114069	0.102003	0.0987658	0.109929	0.110092	0.109231	0.0931479
Slope	0.843638	0.827895	0.905522	0.903298	0.86307	0.849246	0.846406	0.934337
Intercept	0.0181949	0.026998	0.00808558	0.00648146	0.0110236	0.0146464	0.0175078	-0.0125049
R^2	0.792926	0.771161	0.818136	0.830234	0.785363	0.793389	0.793629	0.833003
$\|\Delta E\|$	0.318773	0.292125	0.251107	0.313711	0.269951	0.322538	0.319029	0.262741
ΔE	-0.142651	-0.13066	-0.104081	-0.159816	-0.110822	-0.123119	-0.131637	-0.132478
$E_{\text{comp}}/E_{\text{exp}}$	0.967033	0.968746	0.976345	0.962542	0.975946	0.972218	0.970515	0.967978

Table S16: Table: f_{comp} vs $n(\tilde{\nu})f_{\text{exp}}$ PCM/TD-DFT/6-311++G**/p-gauge/Exact Band Limits/

	BLYP	N12	OLYP	PBE	SOGGA11	SVWN	SVWN5	TPSS
MAE	0.109135	0.113314	0.100367	0.0975116	0.108248	0.109699	0.108711	0.0938189
Slope	0.817384	0.800786	0.876699	0.873878	0.837242	0.820201	0.816992	0.867917
Intercept	0.0187475	0.0279522	0.00930113	0.00704207	0.0128159	0.0144323	0.0174777	-0.0105959
R^2	0.800062	0.777179	0.823971	0.836145	0.78862	0.801487	0.801327	0.837108
$\|\Delta E\|$	0.317859	0.248341	0.250615	0.313127	0.269591	0.363919	0.317394	0.263358
ΔE	-0.140659	-0.080924	-0.103013	-0.157679	-0.10605	-0.174286	-0.130143	-0.131349
$E_{\text{comp}}/E_{\text{exp}}$	0.967513	0.982895	0.976661	0.963072	0.977254	0.958029	0.970875	0.968233

Table S17: Table: f_{comp} vs $n(\tilde{\nu})f_{\text{exp}}$ PCM/TD-DFT/6-311++G**/xp-gauge/Exact Band Limits/

	BLYP	N12	OLYP	PBE	SOGGA11	SVWN	SVWN5	TPSS
MAE	0.109009	0.113644	0.101122	0.0980405	0.109008	0.109758	0.108825	0.0929466
Slope	0.830389	0.81423	0.890916	0.888358	0.850058	0.834554	0.831558	0.90046
Intercept	0.0183145	0.0273229	0.00859011	0.00664609	0.0116333	0.0143901	0.0173194	-0.0116275
R^2	0.796756	0.774427	0.821221	0.833355	0.787346	0.79771	0.797848	0.83526
$\|\Delta E\|$	0.318395	0.292132	0.250717	0.313519	0.270043	0.364566	0.31818	0.262834
ΔE	-0.141784	-0.130215	-0.1034	-0.15886	-0.109135	-0.175047	-0.130967	-0.132293
$E_{\text{comp}}/E_{\text{exp}}$	0.967238	0.968852	0.976527	0.962777	0.976426	0.957841	0.970674	0.968006

Improving fit:

Table S18: Table: f_{comp} vs $n(\tilde{\nu})f_{\text{exp}}$ PCM/TD-DFT/6-311++G**/x-gauge/Improved Fit/

	BLYP	N12	OLYP	PBE	SOGGA11	SVWN	SVWN5	TPSS
MAE	0.03495	0.0373816	0.0320579	0.0315718	0.0528708	0.0281255	0.0281887	0.0350929
Slope	0.945484	0.909869	0.9225	0.944599	0.852115	1.00766	1.00239	0.985708
Intercept	-0.00142239	0.00579609	0.00897798	0.00577367	0.0123698	-0.0137805	-0.0132755	-0.0133078
R^2	0.972068	0.967257	0.972522	0.9774	0.940925	0.987836	0.987943	0.977661
$\|\Delta E\|$	0.329047	0.408021	0.363056	0.317394	0.54864	0.326157	0.323567	0.304902
ΔE	-0.192444	-0.200766	-0.221274	-0.177198	-0.313788	-0.175185	-0.17572	-0.147103
$E_{\text{comp}}/E_{\text{exp}}$	0.952699	0.945545	0.940949	0.955185	0.917368	0.954099	0.955205	0.961602

Table S19: Table: f_{comp} vs $n(\tilde{\nu})f_{\text{exp}}$ PCM/TD-DFT/6-311++G**/p-gauge/Improved Fit/

	BLYP	N12	OLYP	PBE	SOGGA11	SVWN	SVWN5	TPSS
MAE	0.0320563	0.0403879	0.0358432	0.0328095	0.0554908	0.0293895	0.0296605	0.0372253
Slope	0.919583	0.89412	0.913288	0.946842	0.840591	0.993395	0.98452	0.927465
Intercept	0.0145314	0.00893696	0.0108431	0.00468574	0.0128496	-0.0113267	-0.0104811	0.00190136
R^2	0.974004	0.964602	0.965969	0.976969	0.937649	0.984774	0.986233	0.973025
$\|\Delta E\|$	0.316848	0.383454	0.356656	0.321416	0.469033	0.323879	0.321771	0.331648
ΔE	-0.15887	-0.203916	-0.20252	-0.153581	-0.28194	-0.165807	-0.19294	-0.130673
$E_{\text{comp}}/E_{\text{exp}}$	0.959628	0.943803	0.94569	0.959858	0.924351	0.956316	0.949782	0.964062

Table S20: Table: f_{comp} vs $n(\tilde{\nu})f_{\text{exp}}$ PCM/TD-DFT/6-311++G**/xp-gauge/Improved Fit/

	BLYP	N12	OLYP	PBE	SOGGA11	SVWN	SVWN5	TPSS
MAE	0.0378392	0.0348879	0.0332034	0.0321982	0.0533376	0.0297387	0.0293642	0.0358629
Slope	0.930762	0.907329	0.925801	0.935264	0.849312	0.99758	0.993529	0.959303
Intercept	0.00484354	0.00846731	0.00659889	0.00823796	0.0120851	-0.0136527	-0.0137901	-0.007239
R^2	0.971506	0.973811	0.971609	0.976656	0.93815	0.985535	0.987844	0.977555
$\|\Delta E\|$	0.322602	0.401474	0.366498	0.313258	0.541168	0.328383	0.331431	0.340354
ΔE	-0.200335	-0.198175	-0.208389	-0.161754	-0.325098	-0.178985	-0.206515	-0.151144
$E_{\text{comp}}/E_{\text{exp}}$	0.950974	0.945647	0.944053	0.958517	0.914399	0.953449	0.946912	0.960069

S4.3.2 Pure functionals / 6-311++G**: $f^{\text{S}}_{\text{comp}}$ vs $n(\tilde{\nu})f_{\text{exp}}$

Table S21: Table: $f^{\text{S}}_{\text{comp}}$ vs $n(\tilde{\nu})f_{\text{exp}}$ PCM/TD-DFT/6-311++G**/x-gauge/Exact Band Limits/

	BLYP	N12	OLYP	PBE	SOGGA11	SVWN	SVWN5	TPSS
MAE	0.119968	0.126347	0.120031	0.112769	0.125702	0.118644	0.118603	0.107272
Slope	0.999736	0.97814	1.06924	1.06448	1.01261	1.00302	0.999783	1.10197
Intercept	0.0311358	0.0439862	0.0211473	0.0183305	0.0243529	0.0273667	0.0309279	-0.005911
R^2	0.797885	0.773687	0.828233	0.839823	0.789362	0.797079	0.797166	0.841908
$\|\Delta E\|$	0.315878	0.289819	0.248985	0.311166	0.268275	0.320664	0.317228	0.259996
ΔE	-0.139577	-0.125471	-0.0986824	-0.156857	-0.107115	-0.119612	-0.128476	-0.129688
$E_{\text{comp}}/E_{\text{exp}}$	0.967754	0.969916	0.977563	0.963272	0.97687	0.973023	0.971252	0.968651

Table S22: Table: $f^{\text{S}}_{\text{comp}}$ vs $n(\tilde{\nu})f_{\text{exp}}$ PCM/TD-DFT/6-311++G**/p-gauge/Exact Band Limits/

	BLYP	N12	OLYP	PBE	SOGGA11	SVWN	SVWN5	TPSS
MAE	0.113584	0.119941	0.112319	0.105002	0.119626	0.110446	0.110639	0.093165
Slope	0.969786	0.947603	1.03669	1.0308	0.982555	0.96979	0.966184	1.02488
Intercept	0.0315317	0.0447624	0.0221025	0.0186156	0.0259926	0.026734	0.0304944	-0.00440767
R^2	0.805376	0.780034	0.834731	0.846086	0.793146	0.805461	0.805152	0.846169
$\|\Delta E\|$	0.370304	0.344844	0.377835	0.365498	0.393311	0.416944	0.413348	0.260724
ΔE	-0.192515	-0.17928	-0.22615	-0.20973	-0.229129	-0.225751	-0.234603	-0.128482
$E_{\text{comp}}/E_{\text{exp}}$	0.956074	0.958024	0.952832	0.951619	0.952628	0.946654	0.944886	0.968929

Table S23: Table: $f^{\text{S}}_{\text{comp}}$ vs $n(\tilde{\nu})f_{\text{exp}}$ PCM/TD-DFT/6-311++G**/xp-gauge/Exact Band Limits/

	BLYP	N12	OLYP	PBE	SOGGA11	SVWN	SVWN5	TPSS
MAE	0.116618	0.122955	0.116032	0.108552	0.122239	0.114222	0.114155	0.0989655
Slope	0.984589	0.962699	1.05261	1.0475	0.997593	0.986189	0.98284	1.06251
Intercept	0.0311195	0.0441656	0.0215517	0.018316	0.024929	0.026876	0.0304779	-0.00521803
R^2	0.80199	0.777139	0.831604	0.843244	0.791786	0.801593	0.801685	0.844269
$\|\Delta E\|$	0.315687	0.289893	0.248718	0.310956	0.268227	0.362575	0.316393	0.260247
ΔE	-0.138705	-0.124989	-0.0980258	-0.155935	-0.105687	-0.171513	-0.127844	-0.129389
$E_{\text{comp}}/E_{\text{exp}}$	0.967962	0.970037	0.977745	0.9635	0.977287	0.958647	0.9714	0.968714

Improving fit:

Table S24: Table: $f^{\mathrm{S}}_{\mathrm{comp}}$ vs $n(\tilde{\nu})f_{\mathrm{exp}}$ PCM/TD-DFT/6-311++G**/x-gauge/Improved Fit/

	BLYP	N12	OLYP	PBE	SOGGA11	SVWN	SVWN5	TPSS
MAE	0.0287866	0.0287879	0.0318495	0.0279505	0.0391947	0.0299313	0.0287408	0.0322961
Slope	1.01719	0.990217	0.999525	1.01613	0.967247	1.02581	1.02568	1.03611
Intercept	-0.00808012	-0.00517506	-0.00551296	-0.00533848	0.00170796	-0.00650997	-0.00640433	0.00056593
R^2	0.984849	0.983658	0.982347	0.98412	0.966345	0.983387	0.9838	0.982189
$\|\Delta E\|$	0.360351	0.435893	0.469485	0.360281	0.517344	0.362697	0.421744	0.382161
ΔE	-0.181545	-0.188228	-0.264696	-0.193726	-0.29707	-0.190535	-0.262918	-0.238438
$E_{\mathrm{comp}}/E_{\mathrm{exp}}$	0.95565	0.949398	0.931966	0.950287	0.918398	0.95223	0.936659	0.941619

Table S25: Table: $f^{\mathrm{S}}_{\mathrm{comp}}$ vs $n(\tilde{\nu})f_{\mathrm{exp}}$ PCM/TD-DFT/6-311++G**/p-gauge/Improved Fit/

	BLYP	N12	OLYP	PBE	SOGGA11	SVWN	SVWN5	TPSS
MAE	0.0292605	0.0302521	0.0307003	0.0259561	0.0429608	0.0286403	0.0288655	0.0269647
Slope	1.01351	0.99286	0.979139	1.0221	0.930074	1.0105	1.01369	0.999544
Intercept	-0.00850035	-0.00828685	-0.000382229	-0.0033011	0.0113386	-0.0025469	-0.00187497	-0.000410162
R^2	0.985412	0.98331	0.98347	0.988516	0.948752	0.985174	0.984833	0.98771
$\|\Delta E\|$	0.400944	0.476304	0.454248	0.352807	0.487368	0.359095	0.359576	0.36933
ΔE	-0.18584	-0.230871	-0.247923	-0.183381	-0.259987	-0.202854	-0.225275	-0.21454
$E_{\mathrm{comp}}/E_{\mathrm{exp}}$	0.953911	0.93711	0.935607	0.953006	0.928536	0.95012	0.945	0.947558

Table S26: Table: $f^{\mathrm{S}}_{\mathrm{comp}}$ vs $n(\tilde{\nu})f_{\mathrm{exp}}$ PCM/TD-DFT/6-311++G**/xp-gauge/Improved Fit/

	BLYP	N12	OLYP	PBE	SOGGA11	SVWN	SVWN5	TPSS
MAE	0.0280655	0.0313837	0.0306063	0.0263608	0.0385737	0.0303847	0.0288671	0.0279724
Slope	1.00828	0.98825	0.997801	1.01368	0.944977	1.01923	1.01173	1.01663
Intercept	-0.00822059	-0.00557321	-0.00503433	-0.00698901	0.00945421	-0.00499976	-0.00471716	-0.00162926
R^2	0.986242	0.980635	0.982992	0.987739	0.964908	0.983757	0.984627	0.986495
$\|\Delta E\|$	0.393669	0.429035	0.408577	0.394825	0.482226	0.363357	0.3535	0.382093
ΔE	-0.206619	-0.198738	-0.198131	-0.216186	-0.26993	-0.197471	-0.188239	-0.214554
$E_{\mathrm{comp}}/E_{\mathrm{exp}}$	0.948048	0.946038	0.946177	0.943754	0.926286	0.950607	0.953071	0.947761

S4.4 Hybrid functionals / 6-311++G**

S4.4.1 Hybrid functionals / 6-311++G**: f_{comp} vs $n(\tilde{\nu})f_{\mathrm{exp}}$

Avg $nf_{\mathrm{exp}} = 0.392273$

Table S27: Table: f_{comp} vs $n(\tilde{\nu})f_{\mathrm{exp}}$ PCM/TD-DFT/6-311++G**/x-gauge/Exact Band Limits/

	B3LYP	B3P86	M05	mPW1PW91	O3LYP	PBE0	SOGGA11-X
MAE	0.0550129	0.0564508	0.0544024	0.0535729	0.0631937	0.0540103	0.0704479
Slope	0.944034	0.943153	0.928872	0.956102	0.940889	0.95701	0.987277
Intercept	0.00866236	0.00921465	0.0119219	0.0125704	0.00198034	0.0127063	0.0207532
R^2	0.950269	0.947203	0.952433	0.952417	0.93814	0.951823	0.910074
$\|\Delta E\|$	0.204402	0.272664	0.198134	0.241566	0.23967	0.241994	0.519797
ΔE	-0.0830768	-0.127918	-0.0435347	-0.0523917	-0.157478	-0.0485372	-0.142725
$E_{\mathrm{comp}}/E_{\mathrm{exp}}$	0.979465	0.973132	0.989845	0.987977	0.962063	0.98879	0.976394

Table S28: Table: f_{comp} vs $n(\tilde{\nu})f_{\text{exp}}$ PCM/TD-DFT/6-311++G**/p-gauge/Exact Band Limits/

	B3LYP	B3P86	M05	mPW1PW91	O3LYP	PBE0	SOGGA11-X
MAE	0.0572311	0.058635	0.0555279	0.0547626	0.0638847	0.0551311	0.0665226
Slope	0.907321	0.904191	0.920768	0.916769	0.908339	0.917309	0.946584
Intercept	0.0115351	0.01241	0.0154023	0.0155065	0.0042184	0.0158418	0.0239291
R^2	0.951072	0.947836	0.952983	0.953578	0.938809	0.952611	0.911207
$\|\Delta E\|$	0.20566	0.273651	0.198471	0.241447	0.241221	0.241848	0.518327
ΔE	-0.0850515	-0.129797	-0.0451024	-0.0542829	-0.157247	-0.0504685	-0.143413
$E_{\text{comp}}/E_{\text{exp}}$	0.978958	0.972646	0.989428	0.987478	0.962103	0.988283	0.976205

Table S29: Table: f_{comp} vs $n(\tilde{\nu})f_{\text{exp}}$ PCM/TD-DFT/6-311++G**/xp-gauge/Exact Band Limits/

	B3LYP	B3P86	M05	mPW1PW91	O3LYP	PBE0	SOGGA11-X
MAE	0.0559579	0.05728	0.0548653	0.0534824	0.0630405	0.0538863	0.0678161
Slope	0.925514	0.923533	0.924878	0.936303	0.924476	0.936997	0.966792
Intercept	0.00996261	0.0106584	0.0134965	0.0138873	0.0029649	0.01414	0.0221719
R^2	0.950949	0.947844	0.953017	0.95333	0.938699	0.952536	0.911087
$\|\Delta E\|$	0.205086	0.273217	0.198201	0.241574	0.240272	0.242016	0.519083
ΔE	-0.0841052	-0.128913	-0.0443545	-0.0533368	-0.157354	-0.0495157	-0.143058
$E_{\text{comp}}/E_{\text{exp}}$	0.979199	0.972873	0.989626	0.987727	0.962086	0.988532	0.976302

Improving fit:

Table S30: Table: f_{comp} vs $n(\tilde{\nu})f_{\text{exp}}$ PCM/TD-DFT/6-311++G**/x-gauge/Improved Fit/

	B3LYP	B3P86	M05	mPW1PW91	O3LYP	PBE0	SOGGA11-X
MAE	0.0307337	0.0332003	0.0332421	0.0309595	0.0324832	0.0306105	0.0422374
Slope	0.975216	0.97307	0.985891	0.99718	0.969532	1.00385	1.01568
Intercept	0.0028792	0.00453403	-0.000948952	0.0015569	0.000682416	-0.00110395	0.0149587
R^2	0.982102	0.978167	0.980021	0.982828	0.977323	0.983589	0.971991
$\|\Delta E\|$	0.193616	0.192611	0.192999	0.19798	0.236344	0.190842	0.390208
ΔE	-0.0457117	-0.00965552	-0.00578501	0.0487981	-0.111956	0.047107	0.119581
$E_{\text{comp}}/E_{\text{exp}}$	0.987414	0.995417	0.997593	1.00975	0.970672	1.00945	1.03047

Table S31: Table: f_{comp} vs $n(\tilde{\nu})f_{\text{exp}}$ PCM/TD-DFT/6-311++G**/p-gauge/Improved Fit/

	B3LYP	B3P86	M05	mPW1PW91	O3LYP	PBE0	SOGGA11-X
MAE	0.0320187	0.0341632	0.0301929	0.0305421	0.03045	0.029135	0.0381032
Slope	0.960797	1.02793	0.97739	0.966877	0.947177	0.963397	1.00892
Intercept	0.00776688	-0.016819	0.00265274	0.00692391	0.00515841	0.0117325	0.00975322
R^2	0.979415	0.977109	0.983421	0.984289	0.978743	0.984927	0.977327
$\|\Delta E\|$	0.197106	0.200596	0.19792	0.196531	0.245384	0.197034	0.450831
ΔE	-0.0279561	0.0115091	0.0132847	0.0632514	-0.0867494	0.0791509	0.0673467
$E_{\text{comp}}/E_{\text{exp}}$	0.991018	0.999344	1.00272	1.01264	0.976161	1.01603	1.01828

Table S32: Table: f_{comp} vs $n(\tilde{\nu})f_{\text{exp}}$ PCM/TD-DFT/6-311++G**/xp-gauge/Improved Fit/

	B3LYP	B3P86	M05	mPW1PW91	O3LYP	PBE0	SOGGA11-X
MAE	0.0301871	0.0323013	0.030785	0.0304518	0.0336363	0.0302587	0.0397566
Slope	0.965823	0.954891	0.977621	0.98238	0.968617	0.986058	1.00007
Intercept	0.00546139	0.00714804	0.00355436	0.00447434	-0.00295186	0.00333788	0.0157469
R^2	0.981891	0.979292	0.982736	0.983918	0.974593	0.984115	0.973981
$\|\Delta E\|$	0.193252	0.200064	0.188267	0.200216	0.244374	0.200872	0.391226
ΔE	-0.0351608	-0.0023011	-0.000919651	0.0592285	-0.104613	0.0583384	0.12099
$E_{\text{comp}}/E_{\text{exp}}$	0.989369	0.996697	0.998906	1.01197	0.972356	1.01153	1.03066

S4.4.2 Hybrid functionals / 6-311++G**: $f^{\mathbf{S}}_{\mathbf{comp}}$ vs $n(\tilde{\nu})f_{\mathbf{exp}}$

Table S33: Table: $f^{\mathrm{S}}_{\mathrm{comp}}$ vs $n(\tilde{\nu})f_{\mathrm{exp}}$ PCM/TD-DFT/6-311++G**/x-gauge/Exact Band Limits/

	B3LYP	B3P86	M05	mPW1PW91	O3LYP	PBE0	SOGGA11-X
MAE	0.0801308	0.0813192	0.0771689	0.0882947	0.0764474	0.0893263	0.130666
Slope	1.11475	1.10978	1.09454	1.1265	1.11306	1.12758	1.16288
Intercept	0.0198149	0.0205692	0.0241947	0.0249215	0.0113847	0.0250694	0.0352065
R^2	0.956114	0.952655	0.95619	0.9551	0.945938	0.954716	0.91369
$\|\Delta E\|$	0.202672	0.271748	0.197114	0.240968	0.237094	0.24133	0.519417
ΔE	-0.0810563	-0.12692	-0.0425722	-0.051931	-0.154151	-0.04796	-0.142532
$E_{\mathrm{comp}}/E_{\mathrm{exp}}$	0.97992	0.973359	0.99004	0.988072	0.96284	0.988907	0.976439

Table S34: Table: $f^{\mathrm{S}}_{\mathrm{comp}}$ vs $n(\tilde{\nu})f_{\mathrm{exp}}$ PCM/TD-DFT/6-311++G**/p-gauge/Exact Band Limits/

	B3LYP	B3P86	M05	mPW1PW91	O3LYP	PBE0	SOGGA11-X
MAE	0.0705861	0.0714197	0.0783208	0.0772834	0.0677508	0.0783439	0.116381
Slope	1.07224	1.06449	1.08541	1.08092	1.07547	1.08155	1.11572
Intercept	0.0230604	0.024175	0.0285018	0.0282171	0.0139916	0.0285739	0.0388043
R^2	0.95638	0.952783	0.955745	0.955323	0.945921	0.9545	0.913574
$\|\Delta E\|$	0.203833	0.272686	0.197659	0.240758	0.238701	0.241138	0.5179
ΔE	-0.0829479	-0.128723	-0.0440884	-0.0538052	-0.153478	-0.0498602	-0.143178
$E_{\mathrm{comp}}/E_{\mathrm{exp}}$	0.979428	0.972891	0.989634	0.987576	0.963001	0.988405	0.97626

Table S35: Table: $f^{\mathrm{S}}_{\mathrm{comp}}$ vs $n(\tilde{\nu})f_{\mathrm{exp}}$ PCM/TD-DFT/6-311++G**/xp-gauge/Exact Band Limits/

	B3LYP	B3P86	M05	mPW1PW91	O3LYP	PBE0	SOGGA11-X
MAE	0.0747926	0.0757095	0.0775326	0.0822321	0.0716432	0.0832492	0.123118
Slope	1.09337	1.08706	1.09015	1.10362	1.09418	1.10432	1.13921
Intercept	0.0212208	0.0221739	0.0261068	0.0263397	0.0124878	0.0266961	0.036782
R^2	0.95657	0.953096	0.956324	0.955634	0.946225	0.95504	0.914141
$\|\Delta E\|$	0.203333	0.272231	0.19725	0.240929	0.237623	0.241323	0.518669
ΔE	-0.0820169	-0.127833	-0.043381	-0.0528449	-0.153839	-0.0488911	-0.142853
$E_{\mathrm{comp}}/E_{\mathrm{exp}}$	0.979671	0.97312	0.989824	0.987829	0.962913	0.988659	0.976349

Improving fit:

Table S36: Table: $f^{\mathrm{S}}_{\mathrm{comp}}$ vs $n(\tilde{\nu})f_{\mathrm{exp}}$ PCM/TD-DFT/6-311++G**/x-gauge/Improved Fit/

	B3LYP	B3P86	M05	mPW1PW91	O3LYP	PBE0	SOGGA11-X
MAE	0.0500639	0.0524766	0.0559474	0.0637297	0.0379479	0.0646621	0.0884161
Slope	1.05859	1.05398	1.05213	1.07734	1.0115	1.08031	1.10936
Intercept	0.0116571	0.0155656	0.0151556	0.0137363	0.00704377	0.0140072	0.030341
R^2	0.975061	0.972858	0.968469	0.96479	0.975485	0.965408	0.951394
$\|\Delta E\|$	0.235322	0.297357	0.216248	0.272788	0.274371	0.272436	0.438729
ΔE	-0.0436985	-0.0983501	-0.0362535	-0.0306787	-0.1351	-0.0254657	0.040868
$E_{\mathrm{comp}}/E_{\mathrm{exp}}$	0.989993	0.980866	0.992221	0.993844	0.967549	0.994978	1.01281

Table S37: Table: $f^{\mathrm{S}}_{\mathrm{comp}}$ vs $n(\tilde{\nu})f_{\mathrm{exp}}$ PCM/TD-DFT/6-311++G**/p-gauge/Improved Fit/

	B3LYP	B3P86	M05	mPW1PW91	O3LYP	PBE0	SOGGA11-X
MAE	0.0418703	0.0446858	0.0542979	0.052555	0.0338116	0.0523645	0.0727555
Slope	1.0286	1.01804	1.04289	1.05259	1.00849	1.04538	1.07647
Intercept	0.0131839	0.0165337	0.0191888	0.0187297	0.00675583	0.022164	0.0266747
R^2	0.980848	0.977084	0.971437	0.978053	0.978646	0.978025	0.959031
$\|\Delta E\|$	0.234998	0.304216	0.220068	0.267149	0.278756	0.26626	0.490472
ΔE	-0.041959	-0.0979775	-0.0158354	-0.0275063	-0.124732	-0.0234895	-0.0207755
$E_{\mathrm{comp}}/E_{\mathrm{exp}}$	0.990421	0.981073	0.996536	0.994608	0.969807	0.995473	0.997607

Table S38: Table: $f^{\mathrm{S}}_{\mathrm{comp}}$ vs $n(\tilde{\nu})f_{\mathrm{exp}}$ PCM/TD-DFT/6-311++G**/xp-gauge/Improved Fit/

	B3LYP	B3P86	M05	mPW1PW91	O3LYP	PBE0	SOGGA11-X
MAE	0.04565	0.048225	0.0538618	0.05802	0.0343437	0.0585084	0.0813179
Slope	1.04398	1.03241	1.04775	1.07464	1.00009	1.07128	1.0909
Intercept	0.012102	0.0161234	0.0181848	0.0143584	0.00723057	0.0171736	0.029853
R^2	0.978422	0.974247	0.972171	0.974679	0.977968	0.974907	0.95444
$\|\Delta E\|$	0.235063	0.3051	0.214821	0.270479	0.28693	0.268726	0.444065
ΔE	-0.0428835	-0.100003	-0.0170101	-0.0243195	-0.119485	-0.0194825	0.0362677
$E_{\mathrm{comp}}/E_{\mathrm{exp}}$	0.990195	0.98052	0.996365	0.995233	0.97107	0.996398	1.01215

S4.5 Long-range corrected functionals / 6-311++G**

S4.5.1 Long-range corrected functionals / 6-311++G**: f_{comp} vs $n(\tilde{\nu})f_{\mathrm{exp}}$

Avg $nf_{\mathrm{exp}} = 0.392273$

Table S39: Table: f_{comp} vs $n(\tilde{\nu})f_{\mathrm{exp}}$ PCM/TD-DFT/6-311++G**/x-gauge/Exact Band Limits/

	CAM-B3LYP	LC-ωHPBE	ωB97X-D
MAE	0.0634432	0.116687	0.066355
Slope	1.0079	0.906204	1.02455
Intercept	0.0156098	0.00957171	0.0144512
R^2	0.936271	0.750208	0.93576
$\|\Delta E\|$	0.420311	0.898364	0.440551
ΔE	-0.0291262	-0.36221	-0.0018308
$E_{\mathrm{comp}}/E_{\mathrm{exp}}$	1.00407	0.92963	1.01071

Table S40: Table: f_{comp} vs $n(\tilde{\nu})f_{\mathrm{exp}}$ PCM/TD-DFT/6-311++G**/p-gauge/Exact Band Limits/

	CAM-B3LYP	LC-ωHPBE	ωB97X-D
MAE	0.0601739	0.115773	0.0623837
Slope	0.964101	0.862688	0.979874
Intercept	0.0202882	0.0150391	0.0193321
R^2	0.935397	0.745888	0.934291
$\|\Delta E\|$	0.418271	0.898162	0.438643
ΔE	-0.0303612	-0.362783	-0.00308349
$E_{\mathrm{comp}}/E_{\mathrm{exp}}$	1.00373	0.929488	1.01037

Table S41: Table: f_{comp} vs $n(\tilde{\nu})f_{\mathrm{exp}}$ PCM/TD-DFT/6-311++G**/xp-gauge/Exact Band Limits/

	CAM-B3LYP	LC-ωHPBE	ωB97X-D
MAE	0.0610053	0.115582	0.0639526
Slope	0.985884	0.884291	1.00205
Intercept	0.0177944	0.0121703	0.0167317
R^2	0.936233	0.748514	0.935436
$\|\Delta E\|$	0.419288	0.898277	0.439583
ΔE	-0.0297564	-0.362479	-0.00246667
$E_{\mathrm{comp}}/E_{\mathrm{exp}}$	1.0039	0.929562	1.01054

Improving fit:

Table S42: Table: f_{comp} vs $n(\tilde{\nu})f_{\text{exp}}$ PCM/TD-DFT/6-311++G**/x-gauge/Improved Fit/

	CAM-B3LYP	LC-ωHPBE	ωB97X-D
MAE	0.0396876	0.07032	0.0430347
Slope	1.01061	1.01788	1.00349
Intercept	0.0181954	-0.000912432	0.0217659
R^2	0.975419	0.893035	0.971011
$\lvert\Delta E\rvert$	0.256841	0.493216	0.285782
ΔE	0.225406	0.327903	0.263577
$E_{\text{comp}}/E_{\text{exp}}$	1.05338	1.07976	1.0622

Table S43: Table: f_{comp} vs $n(\tilde{\nu})f_{\text{exp}}$ PCM/TD-DFT/6-311++G**/p-gauge/Improved Fit/

	CAM-B3LYP	LC-ωHPBE	ωB97X-D
MAE	0.0352982	0.0668874	0.0393153
Slope	0.989442	1.00503	1.00623
Intercept	0.0177723	-0.00326727	0.0166664
R^2	0.979484	0.898057	0.976092
$\lvert\Delta E\rvert$	0.267543	0.617371	0.288444
ΔE	0.239837	0.213837	0.266947
$E_{\text{comp}}/E_{\text{exp}}$	1.05587	1.05413	1.06229

Table S44: Table: f_{comp} vs $n(\tilde{\nu})f_{\text{exp}}$ PCM/TD-DFT/6-311++G**/xp-gauge/Improved Fit/

	CAM-B3LYP	LC-ωHPBE	ωB97X-D
MAE	0.0367355	0.0684411	0.0411113
Slope	1.00636	1.01536	1.00738
Intercept	0.0178426	-0.00324194	0.0170296
R^2	0.979374	0.895747	0.97335
$\lvert\Delta E\rvert$	0.269088	0.565341	0.276218
ΔE	0.241627	0.28188	0.249455
$E_{\text{comp}}/E_{\text{exp}}$	1.05637	1.06929	1.05827

S4.5.2 Long-range corrected functionals / 6-311++G**: $f^{\text{S}}_{\text{comp}}$ vs $n(\tilde{\nu})f_{\text{exp}}$

Table S45: Table: $f^{\text{S}}_{\text{comp}}$ vs $n(\tilde{\nu})f_{\text{exp}}$ PCM/TD-DFT/6-311++G**/x-gauge/Exact Band Limits/

	CAM-B3LYP	LC-ωHPBE	ωB97X-D
MAE	0.125189	0.163346	0.130606
Slope	1.1847	1.06347	1.20432
Intercept	0.0292712	0.0210413	0.0277227
R^2	0.939699	0.747983	0.94018
$\lvert\Delta E\rvert$	0.419651	0.897012	0.440078
ΔE	-0.0290231	-0.361248	-0.00174412
$E_{\text{comp}}/E_{\text{exp}}$	1.00409	0.929812	1.01072

Table S46: Table: $f^{\text{S}}_{\text{comp}}$ vs $n(\tilde{\nu})f_{\text{exp}}$ PCM/TD-DFT/6-311++G**/p-gauge/Exact Band Limits/

	CAM-B3LYP	LC-ωHPBE	ωB97X-D
MAE	0.111976	0.152416	0.117368
Slope	1.13345	1.01263	1.15218
Intercept	0.0347421	0.027433	0.0334254
R^2	0.937869	0.743088	0.937908
$\lvert\Delta E\rvert$	0.417695	0.8969	0.438347
ΔE	-0.0301466	-0.361773	-0.00289493
$E_{\text{comp}}/E_{\text{exp}}$	1.00377	0.92968	1.01041

Table S47: Table: $f^{\mathrm{S}}_{\mathrm{comp}}$ vs $n(\tilde{\nu})f_{\mathrm{exp}}$ PCM/TD-DFT/6-311++G**/xp-gauge/Exact Band Limits/

	CAM-B3LYP	LC-ωHPBE	ωB97X-D
MAE	0.118205	0.157462	0.123565
Slope	1.15908	1.0379	1.1781
Intercept	0.0317899	0.0240398	0.0303511
R^2	0.93928	0.746041	0.939525
$\lvert\Delta E\rvert$	0.41862	0.896972	0.439217
ΔE	-0.029629	-0.361493	-0.0023205
$E_{\mathrm{comp}}/E_{\mathrm{exp}}$	1.00392	0.929749	1.01056

Improving fit:

Table S48: Table: $f^{\mathrm{S}}_{\mathrm{comp}}$ vs $n(\tilde{\nu})f_{\mathrm{exp}}$ PCM/TD-DFT/6-311++G**/x-gauge/Improved Fit/

	CAM-B3LYP	LC-ωHPBE	ωB97X-D
MAE	0.0809658	0.104917	0.0864547
Slope	1.05509	1.10021	1.07143
Intercept	0.0447719	0.00916355	0.0401207
R^2	0.947506	0.884826	0.939993
$\lvert\Delta E\rvert$	0.387876	0.683057	0.410483
ΔE	0.0694121	0.0246259	0.0894385
$E_{\mathrm{comp}}/E_{\mathrm{exp}}$	1.02277	1.01142	1.02829

Table S49: Table: $f^{\mathrm{S}}_{\mathrm{comp}}$ vs $n(\tilde{\nu})f_{\mathrm{exp}}$ PCM/TD-DFT/6-311++G**/p-gauge/Improved Fit/

	CAM-B3LYP	LC-ωHPBE	ωB97X-D
MAE	0.0726703	0.0929453	0.0746324
Slope	1.04669	1.05353	1.04808
Intercept	0.0355473	0.0127135	0.0351926
R^2	0.955851	0.892289	0.952178
$\lvert\Delta E\rvert$	0.430323	0.735208	0.453168
ΔE	0.0144103	-0.0412358	0.0468392
$E_{\mathrm{comp}}/E_{\mathrm{exp}}$	1.0092	0.996774	1.0172

Table S50: Table: $f^{\mathrm{S}}_{\mathrm{comp}}$ vs $n(\tilde{\nu})f_{\mathrm{exp}}$ PCM/TD-DFT/6-311++G**/xp-gauge/Improved Fit/

	CAM-B3LYP	LC-ωHPBE	ωB97X-D
MAE	0.0757163	0.0985855	0.0805908
Slope	1.04235	1.07679	1.05019
Intercept	0.0441352	0.0117876	0.0411544
R^2	0.953832	0.889305	0.943076
$\lvert\Delta E\rvert$	0.39212	0.683785	0.414944
ΔE	0.0794601	0.0254053	0.0989407
$E_{\mathrm{comp}}/E_{\mathrm{exp}}$	1.02551	1.01162	1.03095

S4.6 Dividing the computed OSs/C^S: hybrid and long-range corrected functionals

S4.6.1 Hybrid functionals / 6-311++G**: $f^{\mathbf{S}}_{\mathbf{comp}}/C^S$ vs $n(\tilde{\nu})f_{\mathbf{exp}}$

Table S51: Table: $f^{\mathrm{S}}_{\mathrm{comp}}/C^S$ vs $n(\tilde{\nu})f_{\mathrm{exp}}$ PCM/TD-DFT/6-311++G**/x-gauge/Exact Band Limits/

	B3LYP	B3P86	M05	mPW1PW91	O3LYP	PBE0	SOGGA11-X
MAE	0.0511973	0.0551164	0.0523808	0.05187	0.0620038	0.052073	0.0633129
Slope	0.967858	0.986709	0.949535	0.958957	1.04055	0.952601	0.916311
Intercept	0.0172039	0.0182881	0.0209894	0.021215	0.010643	0.021179	0.0277416
R^2	0.956114	0.952655	0.956191	0.9551	0.945938	0.954716	0.91369
$\lvert\Delta E\rvert$	0.202672	0.271748	0.197114	0.240968	0.237095	0.24133	0.519417
ΔE	-0.0810564	-0.12692	-0.0425723	-0.0519309	-0.154152	-0.0479601	-0.142532
$E_{\mathrm{comp}}/E_{\mathrm{exp}}$	0.97992	0.973359	0.99004	0.988072	0.96284	0.988907	0.976439

Table S52: Table: $f^{\mathrm{S}}_{\mathrm{comp}}/C^S$ vs $n(\tilde{\nu})f_{\mathrm{exp}}$ PCM/TD-DFT/6-311++G**/p-gauge/Exact Band Limits/

	B3LYP	B3P86	M05	mPW1PW91	O3LYP	PBE0	SOGGA11-X
MAE	0.0529373	0.054512	0.0526024	0.0527496	0.0579909	0.0535852	0.0646953
Slope	0.930949	0.946441	0.941616	0.920159	1.00541	0.913715	0.879146
Intercept	0.0200217	0.0214941	0.0247259	0.0240205	0.0130801	0.0241399	0.0305764
R^2	0.95638	0.952783	0.955745	0.955323	0.945921	0.9545	0.913575
$\lvert\Delta E\rvert$	0.203832	0.272686	0.197659	0.240758	0.238701	0.241138	0.5179
ΔE	-0.0829481	-0.128723	-0.0440884	-0.0538052	-0.153478	-0.0498603	-0.143178
$E_{\mathrm{comp}}/E_{\mathrm{exp}}$	0.979428	0.972891	0.989634	0.987576	0.963001	0.988405	0.97626

Table S53: Table: $f^{\mathrm{S}}_{\mathrm{comp}}/C^S$ vs $n(\tilde{\nu})f_{\mathrm{exp}}$ PCM/TD-DFT/6-311++G**/xp-gauge/Exact Band Limits/

	B3LYP	B3P86	M05	mPW1PW91	O3LYP	PBE0	SOGGA11-X
MAE	0.0516887	0.0539019	0.0523911	0.0511861	0.0591905	0.0518371	0.063447
Slope	0.949299	0.966505	0.945728	0.939486	1.0229	0.932957	0.897656
Intercept	0.0184246	0.0197151	0.0226482	0.0224225	0.0116743	0.0225535	0.0289829
R^2	0.95657	0.953096	0.956324	0.955633	0.946225	0.95504	0.914141
$\lvert\Delta E\rvert$	0.203334	0.272231	0.19725	0.240929	0.237623	0.241323	0.518669
ΔE	-0.082017	-0.127833	-0.0433809	-0.0528449	-0.153839	-0.0488911	-0.142853
$E_{\mathrm{comp}}/E_{\mathrm{exp}}$	0.979671	0.97312	0.989824	0.987829	0.962913	0.988659	0.976349

Improving fit:

Table S54: Table: $f^{\mathrm{S}}_{\mathrm{comp}}/C^S$ vs $n(\tilde{\nu})f_{\mathrm{exp}}$ PCM/TD-DFT/6-311++G**/x-gauge/Improved Fit/

	B3LYP	B3P86	M05	mPW1PW91	O3LYP	PBE0	SOGGA11-X
MAE	0.0257267	0.0327175	0.030305	0.0297167	0.0326309	0.0292649	0.0338773
Slope	0.996557	0.983723	0.993284	0.984149	1.01387	0.984396	0.984731
Intercept	0.00423251	0.0133046	0.00414176	0.0121437	0.00423605	0.0113156	0.0145809
R^2	0.987404	0.982287	0.984411	0.985339	0.979736	0.985849	0.981327
$\lvert\Delta E\rvert$	0.218948	0.220034	0.206314	0.186741	0.263943	0.186077	0.409754
ΔE	-0.0432583	-0.0115264	-0.00525971	0.0434262	-0.126289	0.0507824	0.14256
$E_{\mathrm{comp}}/E_{\mathrm{exp}}$	0.989861	0.996472	0.999233	1.00905	0.969596	1.01051	1.03537

Table S55: Table: $f^{\mathrm{S}}_{\mathrm{comp}}/C^S$ vs $n(\tilde{\nu})f_{\mathrm{exp}}$ PCM/TD-DFT/6-311++G**/p-gauge/Improved Fit/

	B3LYP	B3P86	M05	mPW1PW91	O3LYP	PBE0	SOGGA11-X
MAE	0.0282138	0.0305918	0.0293298	0.0270414	0.0306499	0.0265534	0.0330076
Slope	0.973972	0.962473	0.977956	0.97802	0.98835	0.977769	0.978898
Intercept	0.00358458	0.0149432	0.00903217	0.00600393	0.00124977	0.00501388	0.0119951
R^2	0.9861	0.984648	0.984878	0.987435	0.979765	0.987885	0.980997
$\|\Delta E\|$	0.199497	0.223732	0.211407	0.266871	0.251442	0.270296	0.470428
ΔE	-0.0490072	0.000497587	-0.00717847	0.00378697	-0.126514	0.0123246	0.0901703
$E_{\mathrm{comp}}/E_{\mathrm{exp}}$	0.987305	0.998995	0.998842	1.00186	0.969078	1.00366	1.02304

Table S56: Table: $f^{\mathrm{S}}_{\mathrm{comp}}/C^S$ vs $n(\tilde{\nu})f_{\mathrm{exp}}$ PCM/TD-DFT/6-311++G**/xp-gauge/Improved Fit/

	B3LYP	B3P86	M05	mPW1PW91	O3LYP	PBE0	SOGGA11-X
MAE	0.0269847	0.0308204	0.0301772	0.028341	0.0302755	0.0315588	0.0328026
Slope	0.977648	0.978341	0.983723	0.983637	0.998964	0.976687	0.973477
Intercept	0.00446289	0.0122456	0.00702689	0.00575964	0.00288374	0.00518692	0.0136661
R^2	0.987155	0.983885	0.984692	0.986468	0.98072	0.981722	0.981441
$\|\Delta E\|$	0.21555	0.219836	0.209357	0.206222	0.26175	0.249904	0.463801
ΔE	-0.0458414	-0.0105587	-0.00741978	0.071128	-0.123979	-0.0162977	0.0816335
$E_{\mathrm{comp}}/E_{\mathrm{exp}}$	0.989027	0.996872	0.99875	1.01602	0.96993	0.99572	1.02151

S4.6.2 Long-range corrected functionals / 6-311++G**: $f^{\mathbf{S}}_{\mathbf{comp}}/C^S$ vs $n(\tilde{\nu})f_{\mathbf{exp}}$

Table S57: Table: $f^{\mathrm{S}}_{\mathrm{comp}}/C^S$ vs $n(\tilde{\nu})f_{\mathrm{exp}}$ PCM/TD-DFT/6-311++G**/x-gauge/Exact Band Limits/

	CAM-B3LYP	LC-ωHPBE	ωB97X-D
MAE	0.0558249	0.111298	0.0566864
Slope	0.952117	0.848736	0.958054
Intercept	0.0235246	0.0167928	0.0220538
R^2	0.9397	0.747983	0.94018
$\|\Delta E\|$	0.419651	0.897012	0.440078
ΔE	-0.0290231	-0.361248	-0.00174411
$E_{\mathrm{comp}}/E_{\mathrm{exp}}$	1.00409	0.929812	1.01072

Table S58: Table: $f^{\mathrm{S}}_{\mathrm{comp}}/C^S$ vs $n(\tilde{\nu})f_{\mathrm{exp}}$ PCM/TD-DFT/6-311++G**/p-gauge/Exact Band Limits/

	CAM-B3LYP	LC-ωHPBE	ωB97X-D
MAE	0.0570089	0.113087	0.0573576
Slope	0.910927	0.808167	0.916575
Intercept	0.0279213	0.0218937	0.0265903
R^2	0.937869	0.743088	0.937908
$\|\Delta E\|$	0.417695	0.896901	0.438347
ΔE	-0.0301467	-0.361773	-0.00289482
$E_{\mathrm{comp}}/E_{\mathrm{exp}}$	1.00377	0.92968	1.01041

Table S59: Table: $f^{\mathrm{S}}_{\mathrm{comp}}/C^S$ vs $n(\tilde{\nu})f_{\mathrm{exp}}$ PCM/TD-DFT/6-311++G**/xp-gauge/Exact Band Limits/

	CAM-B3LYP	LC-ωHPBE	ωB97X-D
MAE	0.0557034	0.111951	0.0563422
Slope	0.931529	0.828335	0.937194
Intercept	0.0255488	0.0191858	0.0241446
R^2	0.93928	0.746041	0.939525
$\|\Delta E\|$	0.41862	0.896972	0.439216
ΔE	-0.0296288	-0.361493	-0.00232066
$E_{\mathrm{comp}}/E_{\mathrm{exp}}$	1.00392	0.929749	1.01056

Improving fit:

Table S60: Table: $f^{\mathrm{S}}_{\mathrm{comp}}/C^{S}$ vs $n(\tilde{\nu})f_{\mathrm{exp}}$ PCM/TD-DFT/6-311++G**/x-gauge/Improved Fit/

	CAM-B3LYP	LC-ωHPBE	ωB97X-D
MAE	0.0326438	0.064221	0.0350898
Slope	0.971945	0.99673	0.978221
Intercept	0.0233405	-0.0050347	0.0183633
R^2	0.983136	0.900036	0.980067
$\lvert\Delta E\rvert$	0.26067	0.554488	0.355572
ΔE	0.233416	0.238851	0.183796
$E_{\mathrm{comp}}/E_{\mathrm{exp}}$	1.05491	1.06435	1.04818

Table S61: Table: $f^{\mathrm{S}}_{\mathrm{comp}}/C^{S}$ vs $n(\tilde{\nu})f_{\mathrm{exp}}$ PCM/TD-DFT/6-311++G**/p-gauge/Improved Fit/

	CAM-B3LYP	LC-ωHPBE	ωB97X-D
MAE	0.03458	0.0631906	0.0358249
Slope	0.957479	0.969053	0.965879
Intercept	0.0209792	0.0101119	0.0164367
R^2	0.981001	0.896773	0.978881
$\lvert\Delta E\rvert$	0.272478	0.634452	0.3623
ΔE	0.247559	0.213634	0.192776
$E_{\mathrm{comp}}/E_{\mathrm{exp}}$	1.05788	1.05733	1.05025

Table S62: Table: $f^{\mathrm{S}}_{\mathrm{comp}}/C^{S}$ vs $n(\tilde{\nu})f_{\mathrm{exp}}$ PCM/TD-DFT/6-311++G**/xp-gauge/Improved Fit/

	CAM-B3LYP	LC-ωHPBE	ωB97X-D
MAE	0.0327869	0.0631461	0.0349568
Slope	0.957016	0.976496	0.968275
Intercept	0.0232653	-0.00195736	0.0177055
R^2	0.983295	0.899494	0.979932
$\lvert\Delta E\rvert$	0.256832	0.598398	0.350884
ΔE	0.223924	0.164607	0.174388
$E_{\mathrm{comp}}/E_{\mathrm{exp}}$	1.05217	1.04737	1.0455

S5 Plots of the stats for pure, hybrids, and long-range corrected hybrid functionals

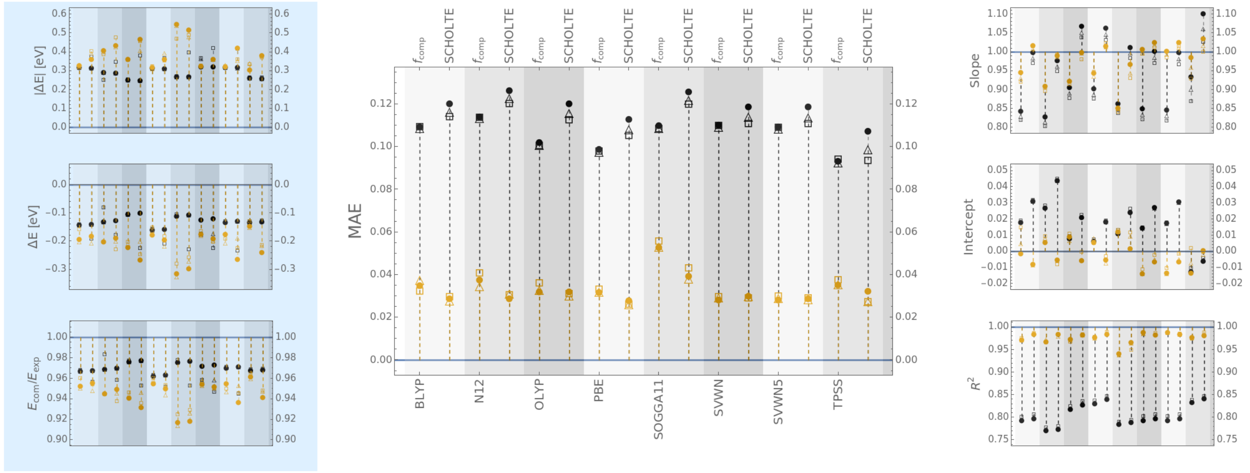

Figure S24: Comparison metrics for the OSs computed with eight pure density functionals. A subset of 76 experimental transitions from the VHHM set was considered. For each method, f_{comp} values (left) and $f^{\text{S}}_{\text{comp}}$ values corrected with Scholte's expression (right), are compared to $n \cdot f_{\text{exp}}$, as indicated by the labels on top of the central plot. Markers in black correspond to transitions assigned within the EBL, while markers in yellow correspond to transitions assigned using the IF mode. A full circle corresponds to the data obtained with the length gauge, an empty square corresponds to the velocity gauge, and an empty triangle corresponds to the mixed gauge. For reference, in the set of transitions considered, the average value of the experimental f-values is $\langle n \cdot f_{\text{exp}} \rangle = 0.392273$. The data displayed can be found in Tables S15 to S26 of the SI.

Table S63: Number of transitions that improve their agreement to experiment with the cavity field correction. (Transitions for which $\left|f^{\text{S}}_{\text{comp},k} - n f_{\text{exp},k}\right|$ is smaller than $|f_{\text{comp},k} - n f_{\text{exp},k}|$). The analysis was made for the length-gauge $f_{\text{comp},k}$ and $f^{\text{S}}_{\text{comp},k}$ values obtained within the IF mode. The results for the pure functionals are presented.

Correction	BLYP	N12	OLYP	PBE	SOGGA11	SVWN	SVWN5	TPSS
Improves	47	51	45	54	54	53	51	51
Does Not	38	34	40	31	31	32	34	34

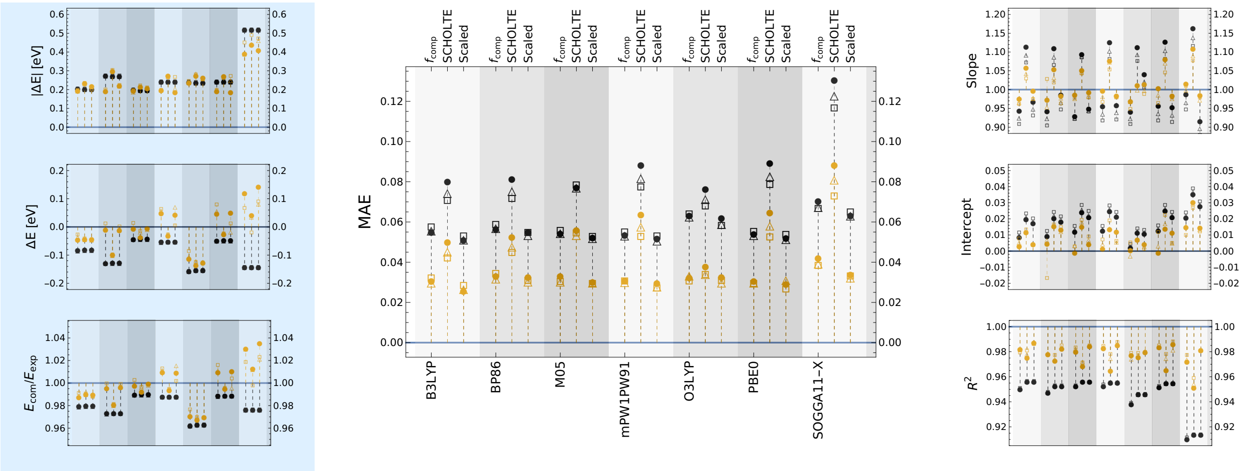

Figure S25: Comparison metrics for the OSs computed with seven hybrid functionals. A subset of 76 experimental transitions from the VHHM set was considered. For each method, f_{comp} values (left), $f^{\text{S}}_{\text{comp}}$ values corrected with Sholte's expression (center), and the scaled $f^{\text{S}}_{\text{comp}}/C^S$ values (right) are compared to $n \cdot f_{\text{exp}}$, as indicated by the labels on top of the central plot. Markers in black correspond to transitions assigned within the EBL, while markers in yellow correspond to transitions assigned using the IF mode. A full circle corresponds to the data obtained with the length gauge, an empty square corresponds to the velocity gauge, and an empty triangle corresponds to the mixed gauge. For reference, in the set of transitions considered, the average value of the experimental f-values is $\langle n \cdot f_{\text{exp}} \rangle = 0.392273$. The data displayed can be found in Tables S27 to S38 and S51 to S56 of the SI.

Table S64: Number of transitions that improve their agreement to experiment with the cavity field correction. The results for the hybrid functionals are presented.

Correction	B3LYP	B3P86	M05	mPW1PW91	O3LYP	PBE0	SOGGA11-X	BHandHLYP
Improves	34	33	30	24	43	23	19	26
Does Not	51	52	55	61	42	62	66	59

76 VHHM transitions

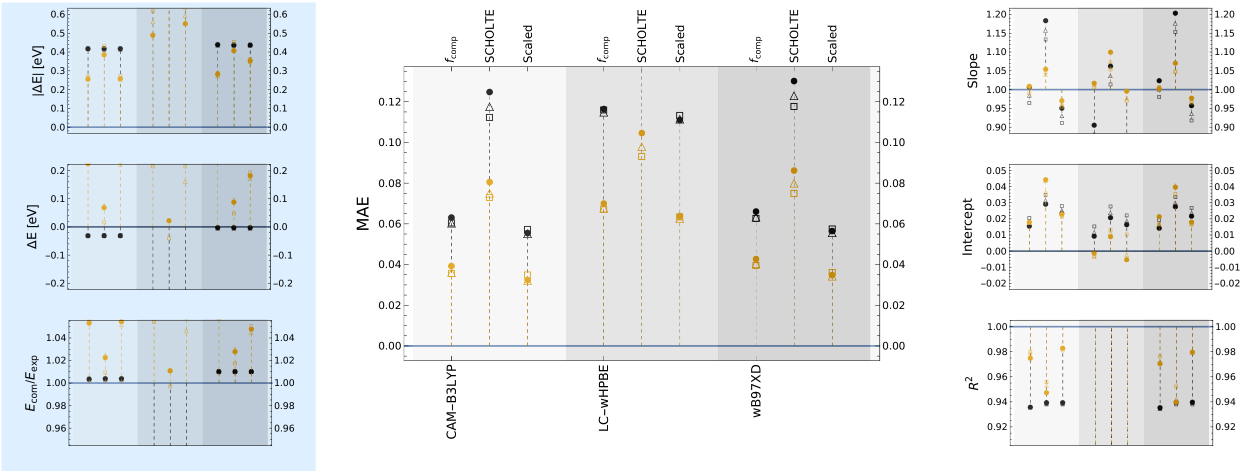

Figure S26: Comparison metrics for the OSs computed with three hybrid, long-range corrected functionals. A subset of 76 experimental transitions from the VHHM set was considered. For each method, f_{comp} values (left), $f^{\mathrm{S}}_{\mathrm{comp}}$ values corrected with Sholte's expression (center), and the scaled $f^{\mathrm{S}}_{\mathrm{comp}}/C^{S}$ values (right) are compared to $n \cdot f_{\mathrm{exp}}$, as indicated by the labels on top of the central plot. Markers in black correspond to transitions assigned within the EBL, while markers in yellow correspond to transitions assigned using the IF mode. A full circle corresponds to the data obtained with the length gauge, an empty square corresponds to the velocity gauge, and an empty triangle corresponds to the mixed gauge. For reference, in the set of transitions considered, the average value of the experimental f-values is $\langle n \cdot f_{\mathrm{exp}} \rangle = 0.392273$. The data displayed can be found in Tables S39 to S50 and S57 to S62 of the SI.

Table S65: Number of transitions that improve their agreement to experiment with the cavity field correction. The results for the long-range corrected functionals are presented.

Correction	CAM-B3LYP	LC-ωHPBE	ωB97X-D
Improves	20	23	18
Does Not	65	62	67
